# Simulating dynamic insecticide selection pressures for resistance management in mosquitoes assuming polygenic resistance

**DOI:** 10.1371/journal.pcbi.1012944

**Published:** 2025-04-28

**Authors:** Neil Philip Hobbs, Ian Hastings

**Affiliations:** 1 Department of Vector Biology, Liverpool School of Tropical Medicine, Liverpool, United Kingdom; 2 Department of Tropical Disease Biology, Liverpool School of Tropical Medicine, Liverpool, United Kingdom; University of Auckland, NEW ZEALAND

## Abstract

Insecticide resistance management (IRM) is critical to maintain the operational effectiveness of insecticides used in public health vector control. Evaluating IRM strategies rests primarily on computational models. Most models assume monogenic resistance, but polygenic resistance may be a more appropriate assumption. Conventionally, polygenic models assume selection differentials are constant over successive generations. We present a dynamic method for calculating the selection differentials accounting for the level of resistance and insecticide efficacy. This allows the inclusion of key parameters namely insecticide dosing, insecticide decay and cross resistance, increasing biological and operational realism. Two methods for calculating the insecticide selection differential were compared: truncation (only the most resistant individuals in the population survive) and probabilistic (individual survival depends on their level of resistance). The probabilistic calculation is extendable to multiple gonotrophic cycles, whereby mosquitoes may encounter different insecticides over their life span. A range of IRM strategies of direct policy relevance can be simulated, including the implication of reduced dose mixtures. We describe in detail the calculation and calibration of these models. We demonstrate the ability of the models to simulate a variety of IRM strategies and implications of including these features of the models. In simple IRM strategy evaluations, the truncation and probabilistic models give comparable results to each other and against published polygenic and monogenic models. Analysis of model simulations indicates there is often little difference between sequences or rotations of insecticides. Full-dose mixtures remain the best evaluated IRM strategy. Consistency between models increases confidence in their predictions especially when demonstrating model assumptions do not significantly impact key operational decisions. Using the multiple-gonotrophic cycle model we calculate the age distributions of mosquitoes which provides a framework to link resistance management with disease transmission. Future applications will investigate more scenario-specific evaluations of IRM strategies to inform public health policy.

## Author summary

Insecticide resistance poses a threat to the continued control of vector-borne diseases. The use of insecticide resistance management strategies is recommended to slow the spread of insecticide resistance and limit its impact on disease transmission. However, what insecticide resistance management strategies work in specific circumstances is under debate. Simulation modelling is frequently used to evaluate insecticide resistance management strategies, as evaluating strategies in the field is inhibited by the need for long trial durations, replicated across settings. Previous models have generally assumed resistance is encoded by a single gene (monogenic), and evaluated a limited set of strategies. Here we consider insecticide resistance when encoded by many genes (polygenic) and use a dynamic quantitative genetics approach. We present a novel mathematical methodology for the simulation of insecticide resistance management strategies, which includes the key parameters of insecticide dosing, insecticide decay and cross resistance. These are generally absent in previous models, despite being considered important for strategy evaluation. Extending our computational model to allow for multiple blood-feeding cycles (and hence multiple rounds of selection) allows for more complex strategies to be evaluated which include household level deployments of different insecticides. In simple scenarios evaluating rotations, sequences and mixtures we generate further support for the results from other recent modelling studies. We further demonstrate that this computational model will have value for evaluating more specific insecticide resistance management scenarios, which can help inform and guide insecticide deployments.

## 1. Introduction

Insecticides are required to control vectors of human diseases, especially malaria. Insecticide-treated nets (ITNs) and indoor residual spray (IRS) are effective at controlling disease transmission but are strong drivers of insecticide resistance (IR) [1]. Pyrethroid ITNs are estimated to be responsible for 68% of the 663 million averted malaria cases between 2000 and 2015 [2]. The inability to control malaria vectors, as may occur as a consequence of IR, could result in a resurgence of malaria transmission [3]. Insecticide resistance management (IRM) strategies (see Table 1 for definitions) have been identified which putatively slow the spread of IR and mitigate any impact of IR on transmission [4].

**Table 1:** Definition and description of terms and acronyms.

Insecticide Arsenal: The total number of insecticides in the simulation; and, for mixtures, which insecticides are available for use in mixtures.
**Cross Resistance:** Positive cross resistance occurs when resistance to one insecticide increases resistance to another insecticide. Negative cross resistance occurs when resistance to one insecticide increases susceptibility to another insecticide.
**Withdrawal Threshold:** The bioassay survival level which constitutes an individual insecticide’s “failure” threshold above which it must be withdrawn from use.
**Return Threshold:** The bioassay survival level which a previously “failed” insecticide much reach before the insecticide can be re-considered for deployment.
**Deployment Interval:** The frequency with which insecticide deployments are scheduled. Note, for combinations; the ITN and IRS can have different deployment intervals. For example, ITNs are scheduled to be re-distributed every 3 years, whereas IRS is scheduled for re-application every year.
**Insecticide Switching Strategy:** Defines the rules regarding how insecticide deployment decisions are made over time. They are generally based on the Withdrawal and Return thresholds defined above.
**Insecticide Deployment Strategy: Defines the way in which insecticides are deployed and is one of:** • **Monotherapy:** the deployment of a single insecticide at any time. • **Mixtures:** the deployment of two insecticides in a single formulation. Mosquitoes encountering the mixture inevitably contact both insecticides. • **Micro-mosaics:** spatial deployments at the household level, where different ITNs or IRS formulations are distributed to different households in the same village. • **Combinations:** simultaneous deployment of ITNs and IRS. A single house can receive both, just the ITN or just the IRS. Mosquitoes entering a house with both may contact both, just the ITN or just the IRS.
Acronyms: IR = Insecticide Resistance IRM = Insecticide Resistance Management PRS = Polygenic Resistance Score ITN = Insecticide-Treated Net IRS = Indoor Residual Spray

Evaluating IRM in field trials is impractical, as they require long study durations and replication over diverse ecological and epidemiological settings. Computational modelling is therefore frequently used for IRM evaluation [5], the value of which is improved if models: (i) cover a range of assumptions regarding the underlying biology of IR; (ii) accurately reflect the circumstances under which IR selection occurs; (iii) show consistency of results between different models or show how inconsistencies arise. Mosquitoes possess a diverse range of physiological resistance mechanisms including target site, metabolic [6] and cuticular resistance [7]. Genome wide association studies have identified IR in mosquitoes to be highly polygenic, because of copy number variation and a large variety of single nucleotide polymorphisms [8]. Most computational models simulating IRM assume resistance is a monogenic trait [9] encoded by mutations in single genes. In reality, insecticide resistance in mosquitoes is unlikely to be either a classically monogenic or polygenic trait (it is more likely to be a mixture of both, with some genes with a large effect segregating within a polygenic background of variable IR levels). Here, we model insecticide resistance as a classically polygenic trait as a counter-factual assumption from previous monogenic models. The key aim is to test whether predictions on optimal IRM strategy differ depending on the underlying genetic assumptions. A secondary aim is to develop a polygenic model that can ultimately be combined computationally with a single gene model to investigate more complex genetic systems of IR.

Our polygenic models of IR are based on the “Breeder’s”/Lush equation [10] as used in some previous studies for example [11–14]. For sexual insects (particularly mosquitoes where only the females forage for blood meals), the sex-specific Breeder’s equation is often more biologically appropriate, accounting for sex-specific insecticide selection:


$$R_{I}^{S}=\;\frac{h_{I}^{2}\left(S_{I}^{\mathrm{\backslash venus}}+S_{I}^{\mathrm{\backslash mars}}\right)}{2}$$ (1a)

The response ($R_{I}^{S}$) is the change in the mean trait value (IR) between generations, the S superscript indicating insecticide selection and the I subscript indicating it is selection for resistance to insecticide I. The selection differentials ($S_{I}^{\mathrm{\backslash venus}}$ and $S_{I}^{\mathrm{\backslash mars}}$) are the within generation change in the mean trait value (i.e., the difference in IR level in individuals at birth and in those surviving to breed). $h^{2}$ is the heritability of the trait.

We previously studied the development of polygenic IR using the “polyres” model [11]. In “polyres”, the selection differentials for each sex were calculated based on insecticide exposure (Equations 3a and 3b in [11]), such that the response to insecticide selection was calculated as follows (Equation 4 in [11]):


$$R_{I}^{S}=\;\beta \left(\frac{h_{I}^{2}x(1+m)}{2}\right)$$ (1b)

xis the level of insecticide exposure (proportion of mosquitoes encountering the insecticide) on female mosquitoes, replacing the explicit selection differential in Equation 1a. m is the level of exposure on male mosquitoes as a proportion of female exposure. Female mosquitoes are more likely to be exposed to ITNs or IRS due to their blood-feeding behaviour. $R_{I}^{S}$ in “polyres” was constant throughout a simulation (x and m were constant), regardless of the level of resistance in the population. The scaling factor (β) accounted for uncertainty in exposure and $h^{2}$, calibrating simulations so IR arises after a realistic timeframe [11].

In this paper we extend the “polyres” methodology to mechanistically calculate the selection differentials allowing inclusion of several key features for IRM strategy performance lacking from most previous models, notably: insecticide dosing at deployment; insecticide decay post-deployment [15]; individuals encountering only a single insecticide exposure per generation; cross resistance between insecticides; polygenic basis of resistance [16]. Including insecticide dosing is required for improved evaluation of mixtures.

Reduced dose mixtures are defined as mixtures where each constituent insecticide partner is deployed at less than their recommended monotherapy application rate; these most likely occur as a compromise designed to reduce the financial costs associated with mixtures [17], as seen with next-generation mixture ITNs. Insecticide decay is defined as the reduction in killing efficacy of the insecticides over time. It is an important consideration in public health because, unlike agriculture where insecticides are often replaced weekly, insecticides used in public health have long residual lifespans, replaced on a timescale of months (IRS) to years (ITNs). Insecticidal activity decays over time due to chemical and/or physical degradation (e.g., fabric damage for ITNs) [18]. Insecticide decay is widely recognised, but its operational impact on IR is unclear [17]. A previous study [15] quantified the effect of insecticide decay and argued, using a single-gene argument, that it could potentially rapidly increase selection for IR. Despite these concerns, to our knowledge, insecticide decay is absent from previous public health IRM models.

**Fig 1:**
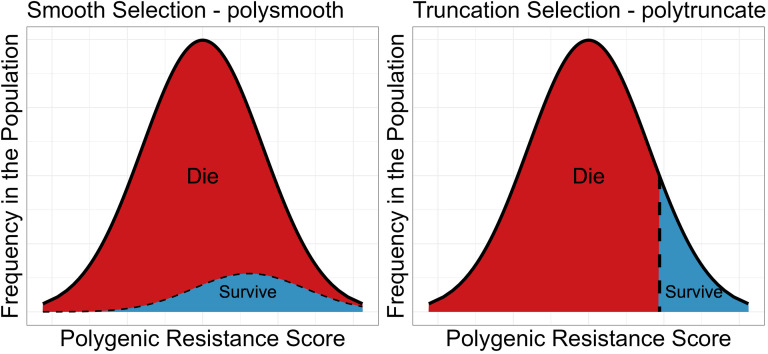
Diagrammatic Representation of Smooth and Truncation Selection. Left panel: Probabilistic Selection (implemented in the “polysmooth” model). Probabilistic Selection [14] defines the survival probability of an individual mosquito to be a function of its own polygenic resistance score (PRS). Note, while it looks like only a small proportion of more resistant individuals survives, this only appears to be the case because there were initially only a smaller proportion of higher resistance individuals. Right panel: Truncation Selection (implemented in the “polytruncate” model). Truncation selection occurs when only mosquitoes with a resistance level above a certain threshold survive insecticide contact. In our model this means that if the mean survival probability of the population was calculated as 10%, the surviving individuals constitute the top 10% of the population. This threshold value is dependent on insecticide efficacy and the level of resistance in the population. The black dashed line indicates the threshold for selection $K_{i}^{F}$ (calculated in Equation 2b(i)) and is therefore the proportion of individuals expected to survive based on the mean PRS of the population. The individuals in red section of the distribution therefore all die, and the individuals in the blue section of the distribution all survive.

Small-scale spatial insecticide deployments of different insecticides may allow foraging female mosquitoes to encounter different insecticides over her lifespan This requires extending standard modelling techniques to allow for multiple gonotrophic cycles [19] (hereby referred to as cycles). Additionally, there is a need to consider how insecticide mortality occurs in the field, so we model this as both probabilistic and truncation selection processes (Fig 1). This requires the development of two model strands (“polysmooth” and “polytruncate”) to ensure assumptions regarding the mechanism of selection do not qualitatively impact the optimal IRM strategy choice.

We present extensions to the previous “polyres” [11] model, using a dynamic quantitative genetics methodology. We present two models (“polytruncate” and “polysmooth”) which implement insecticide selection by truncation and probabilistically respectively. Given the number of added features, we focus this paper on the methodological development of the model and the biological/operational rationale. The methodology described is extensive but essential to incorporate these key features (dosing, decay, multiple gonotrophic cycles, polygenic resistance, and cross resistance) into a single framework. We present the technical capability of the model, focusing on how the model works and performs. This framework will allow scenario-specific IRM strategies to be evaluated under a much wider range of biological and operations assumptions. This will provide new and important insight into the conditions IRM strategies require for effective IRM.

## 2. Methods

### Model summary

The models (“polytruncate” and “polysmooth”) are based on a quantitative genetics framework. The model tracks a “polygenic resistance score” (PRS), which quantifies the “amount” of resistance in a mosquito population. The models use discrete non-overlapping generations, and mosquito populations consist of males and females. The model consists of two patches, an intervention site and a refugia. The events in a generation happen in this order: 1. insecticide selection, 2. mating, 3. dispersal, 4. egg laying. This is consistent with previous models [11,20,21]. Where our models differ from previous models is the level of insecticide selection in each generation depends on mosquito behaviour, IR level, insecticide efficacy and insecticide deployments over space. The intensity of insecticide selection can therefore vary over time depending on these factors. Insecticide selection can occur by truncation or probabilistically (Fig 1), generating two model branches: “polytruncate” and “polysmooth”. Insecticide deployments often involve two or more insecticides, so simulations must track resistance to several insecticides simultaneously. We use lowercase letters i, j, k etc to refer to insecticides and uppercase letters I, J, K etc to refer to the corresponding resistance trait. We describe the model by introducing concepts in a logical development over four methods sections:

- Methods Section 1: Quantification of Insecticide Resistance and Insecticide Efficacy.
- Methods Section 2: Biology of Insecticide Selection.
- Methods Section 3: Biology of Insecticide Selection over Multiple Gonotrophic Cycles.
- Methods Section 4: Model Calibration, and its Application to Evaluating IRM Strategies.

The model methodology requires considerable technical development and detail to allow an expanded range of IRM scenarios to be evaluated. Table 2 provides the key points and underlying assumptions. Table 3 provides a comparison summary between the three polygenic models (“polyres”, “polytruncate” and “polysmooth”). Tables of the parameter symbols used are provided in the S1 File.

**Table 2:** Summary of model methodology and key assumptions. This table summarises and highlights the key points and assumptions for each model development methodology section. Technical details of their implementation and evaluation is given in Methods section 4.

Headline Overview: The dynamic models are quantitative genetic models which track a “polygenic resistance score” (PRS). Selection differentials are calculated based on the PRS and the efficacy of the insecticide(s). Selection is either by truncation (“polytruncate) or probabilistic (“polysmooth”). The “polysmooth” model allows for multiple gonotrophic cycles.
**Methods Section 1: Quantification of Insecticide Resistance and Insecticide Efficacy**
**1.1 Describing and Defining the Quantification of Resistance - Polygenic Resistance Score:** Insecticide resistance level is quantified by the PRS, measured as $z_{I}$. The Hill-variant of the Michaelis-Menten equation converts PRS to bioassay survival. PRS can be accurately measured using bioassays without error. PRS is a Normally distributed trait, with a standard deviation ($\sigma_{I}$). Fitness costs are unable drive the mean PRS (${\overset{―}{z}}_{I}$) below 0.
**1.2 Defining Insecticide efficacy and insecticide decay post-deployment:** Insecticides decay, becoming less effective. Insecticide efficacy is defined as the proportion of fully susceptible mosquitoes killed by contact with the insecticide. Each unique insecticide in the same setting decays at the same rate. All ITNs with insecticide i have a set decay rate. And all IRS with insecticide j have the same decay rate.
**1.3 Converting bioassay survival to/from field survival as a function of insecticide efficacy:** Bioassay survival is correlated with field survival to insecticides. The relationship between bioassay survival and field survival is the same for all insecticides and is linear.
**1.4 Standard deviation of the Polygenic Resistance Score:** The standard deviation of the PRS ($\sigma_{I}$) can be fixed or increase with the mean PRS value (${\overset{―}{z}}_{I}$).
**Methods Section 2: Biology of Insecticide Selection**
**2.1 Calculating the Response to Insecticide Selection:** Response is based on insecticide selection and fitness costs. There are no sub-lethal effects of insecticide selection, a common assumption across IRM models,
**2.2 Calculating Insecticide Selection Differentials** PRS (${\overset{―}{z}}_{I}^{\text{\textbackslash{}mars}}$ and ${\overset{―}{z}}_{I}^{\text{\textbackslash{}venus}}$) are the same in both sexes at the start of each generation. Male and female mosquitoes have different levels of insecticide exposures and hence different selection pressures. Mixtures result in simultaneous contact of both insecticides (survival is multiplicative).
**2.3 Calculating Insecticide Selection Differentials by Truncation Selection (“polytruncate”):** Truncation selection occurs when only the most resistant survive. Truncation point depends on ${\overset{―}{z}}_{I},\;\sigma_{I}$ and the insecticide efficacy.
**2.4 Calculating Insecticide Selection Differentials by smooth (probabilistic) selection (“polysmooth”):** Smooth (probabilistic) selection occurs when survival is dependent on an individual’s PRS and insecticide efficacy.
**2.5 Calculating the Fitness Cost Selection Differential** Implemented as either fixed selection differential (when $\sigma_{I}$ is fixed) or a scaled selection differential (when $\sigma_{I}$ varies with ${\overset{―}{z}}_{I}$). Fitness costs occur within the Normal distribution.
**2.6 Between Generation Changes in Resistance:** Cross resistance (between insecticides) is incorporated using genetic correlations. Model tracks discrete non-overlapping generations. We assume the only form of mortality is induced by the deployment of insecticides (assumption relaxed when assuming multiple cycles) because the natural mortality rate would be a fixed rate for all insects irrespective of their PRS value.
**2.7 Mosquito Dispersal:** Model has two patches: intervention and refugia. Insecticides are only deployed in the intervention site. Mosquitoes can disperse between the two patches. Mating and selection occur before dispersal. All female mosquitoes successfully mate and mating is random.
**Methods Section 3 Biology of Insecticide Selection Over Multiple Gonotrophic Cycles**
**3.1 Defining Coverages, Encounter Probabilities and Natural Survival:** Insecticides can be deployed as monotherapies, mixtures, micro-mosaics or combinations. Micro-mosaics and combinations can have unequal coverages of each insecticide, e.g., for micro-mosaics $c_{i}$ = 0.4 and $c_{j}$ = 0.6. Mosquitoes entering a house with a combination (ITN + IRS) can encounter ITN, IRS or both. Natural survival (not due to insecticides) is implemented at the end of each cycle. Insecticide coverages and encounter probabilities are fixed throughout each simulation.
**3.2 Calculating the Response to Selection with multiple gonotrophic cycles:** Multiple cycles are only implemented in “polysmooth” (survival is probabilistic). Insecticide selection occurs in each cycle. Male mosquitoes are not tracked over multiple cycles, as all females successfully mate in their first cycle. Fitness cost selection differential only implemented in the first cycle and is carried forward to all subsequent cycles. Mosquito age does not impact survival to insecticides. Next generation consists of all eggs laid in the previous generation over all cycles.
**3.3 Calculating the insecticide selection differential for each gonotrophic cycle (“polysmooth”):** Selection differential and response calculated for each cycle. Final response is weighted by number of eggs laid in each cycle. Number of females is proportional to the number of eggs laid.
**S5 File: Calculating the Male Insecticide Selection Differential with Complex Insecticide Encounters.** Male mosquitoes have only one round of selection. Male mosquitoes encounter insecticides prior to mating.
**S6 File: Multiple Gonotrophic Cycles and Dispersal.** Female mosquitoes can disperse between intervention site and refugia in each cycle.

**Table 3:** Summary of the capabilities of the dynamic model “polysmooth” and “polytruncate” compared to the previously published “polyres” model.

	Polytruncate	Polysmooth	Polyres
**Insecticide Deployment Options**			
Monotherapies	Yes	Yes	Yes
Mixtures	Yes	Yes	Yes
Combinations	No	Yes	No
Micro-Mosaics	No	Yes	No
Number of Insecticides in Arsenal	Technically Unlimited	Technically Unlimited	Technically unlimited
**Parameter Variables**			
Cross Resistance	Yes, as correlated responses.	Yes, as correlated responses.	Yes, as correlated responses.
Insecticide Dosing	Variable (unique per insecticide)	Variable (unique per insecticide)	Fixed, full-concentration only.
Insecticide Decay	Yes (unique per insecticide)	Yes (unique per insecticide)	No
Mechanism of Selection	Truncation	Probabilistic	Not defined
Multiple gonotrophic cycles	No	Yes (with natural mortality between gonotrophic cycles)	No
Linkage disequilibrium.	Does not explicitly account for linkage disequilibrium	Does not explicitly account for linkage disequilibrium	Does not explicitly account for linkage disequilibrium

The computational models are written in R. Model code is available from the GitHub repositories: https://github.com/NeilHobbs/polytruncate and https://github.com/NeilHobbs/polysmooth.

### Methods Section 1: Quantification of Insecticide Resistance and Insecticide Efficacy

#### Methods Section 1.1: Describing and Defining the Quantification of Resistance - Polygenic Resistance Score.

**Fig 2:**
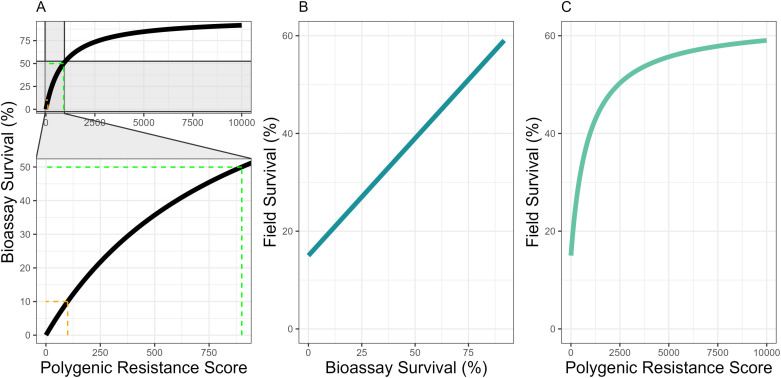
The Polygenic Resistance Score (PRS) relationship with Bioassay Survival and Field Survival. Panel A shows the Polygenic Resistance Score (PRS) on a scale of 0 to 10000 (Equation 2a), the zoomed inset shows the same data on a scale from 0 and 1000, values especially important in the evaluation of novel insecticides. The orange line indicates 10% bioassay survival (PRS = 100) (our default for insecticidal withdrawal), when $z_{50}$ = 900 (green line). Panel B is the relationship between bioassay survival and field survival (Equation 2b), and Panel C is the relationship between the polygenic resistance score and field survival. These are identical to the relationships used in [11].


We use the “Polygenic Resistance Score” (PRS), to quantify the level of IR as previously described in the “polyres” methodology [11]. The PRS is an arbitrary scale of IR [11] using the Hill-variant of the Michaelis-Menten equation (where n=1), to convert a “level of IR” to insecticide i ($z_{I}$) to the measured bioassay survival ($K_{i}^{B}$) (Fig 2).

$$K_{i}^{B}=\frac{K_{\max}*{z_{I}}^{n}}{z_{50}+{z_{I}}^{n}}$$ (2a)

$z_{50}$(user-defined) is the PRS giving 50% bioassay survival. $K_{\max}$ is the maximum proportion of mosquitoes surviving in a bioassay, which is, by definition, 1. All calibration and parameterization used $z_{50}$ = 900. This gives $z_{I}$=100 as having a 10% bioassay survival, a commonly suggested criterion for confirmed resistance [22]. A graphical representation of the relationship between PRS and bioassay survival is given in Fig 2.

The model tracks the mean PRS in a mosquito population over discrete non-overlapping generations, but is reported as bioassay survival for interpretability. However, bioassay survival is not field survival. Bioassays for measuring IR are highly standardised using 2–5 day old non-bloodfed females, with mortality assessed against a fixed concentration of insecticide for a fixed time-period [22]. Field exposures can vary in both duration and insecticide concentration for any mosquito-insecticide encounter. Mosquito mortality, for females only, in bioassays has been found to be correlated with survival in experimental huts [23]. We model the relationship between bioassay survival and experimental hut survival, thereby converting bioassay survival (given a PRS) into the corresponding experimental hut survival, an approximation for field survival.


$$K_{i}^{F}=\;\varphi_{1}K_{i}^{B}+\varphi_{2}$$ (2b)

$K_{i}^{F}$ is survival to insecticide i under field conditions. $\varphi_{1}$ and $\varphi_{2}$ are regression coefficients obtained from linear modelling, $\phi_{1}$ = 0.48 and $\phi_{2}$ = 0.15 [11]. The relationship between bioassay survival and field survival, and the relationship between PRS and field survival is presented in Fig 2. Equation 2b is identical to Equation 1b in [11].

#### Methods Section 1.2: Defining insecticide efficacy and insecticides decay post-deployment.

Insecticides decay over time, a worrying process in control programmes that results in reduced insecticide effectiveness and, in all probability, a change in the intensity of selection for resistance. Insecticide decay is routinely absent from models of IR evolution; see discussion with examples in [15].

Ideally insecticides are deployed at the manufacturers’ specified concentration after which insecticide concentrations are likely to decay exponentially after application:


$$\zeta_{\tau }=\zeta_{0}e^{k\tau }$$ (2c)

Insecticides are deployed at an initial concentration ($\zeta_{0}$) at time zero, decaying at rate k each mosquito generation (τ) to give the insecticide concentration ($\zeta_{\tau }$) at τ generations post deployment. Insecticide concentration must be converted to efficacy in terms of killing the key insect target species. We define insecticide efficacy as the ability of the insecticide to kill fully susceptible mosquitoes ($z_{I}\leq 0$). Therefore, insecticides deployed at the manufacturers’ specified concentrations such as a brand new single-insecticide ITN have an initial insecticide efficacy ($\omega_{0}^{i}$) of 1.

**Fig 3:**
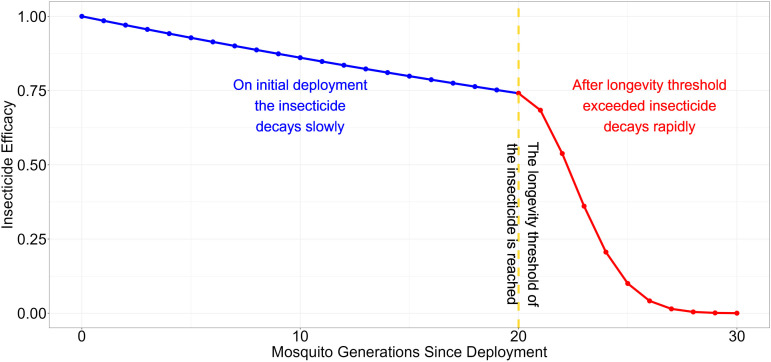
An example insecticide decay profile for an ITN. In this example the insecticide is deployed at its manufacturers recommended concentration with an initial deployed insecticide efficacy of $\omega_{0}^{i}$=1. It is a two-step function. Step 1 describes the first two years (~20 mosquito generations) as the insecticide decays slowly at its base decay rate (blue line). Step 2 occurs after the decay threshold ($\tau_{b}^{i}$) has been reached (vertical yellow line) and the insecticide efficacy decays rapidly (red line). In this example the base decay rate ${\delta_{b}}^{i}$ was 0.015, the rapid decay rate ${\delta_{r}}^{i}$ was 0.08 and the decay threshold $\tau_{b}^{i}$ was 20 generations (~2 years). These values are user-defined inputs and can be varied allowing insecticides to decay faster/slower or sooner/later. Setting ${\delta_{b}}^{i}$ and ${\delta_{r}}^{i}$ to 0 prevents insecticide decay from occurring (a key assumption in previous models). Insecticides in mixture can have different decay properties. Setting decay rates to be identical before and after the threshold eliminates the second phase and converts the decay profile to a single dynamic if required.


Insecticide decay depends on both insecticide chemistry and environmental conditions [24]. Decay may be slow initially, becoming rapid after the intended longevity of the product is exceeded. Fig 3 shows an example insecticide decay profile. In stage 1 (blue part of Fig 3) the insecticide is newly deployed, and the decay rate is low. Insecticide efficacy at time τ after deployment is calculated as follows:

$$\omega_{\tau }^{i}=\;\omega_{0}^{i}*\exp\left(-\tau^{2}*\;{\delta_{b}}^{i}\right),\;where\;0\leq \tau \leq \;\tau_{b}^{i}$$ (2d(i))

Where $\omega_{\tau }^{i}$ is the efficacy of insecticide i at time τ since deployment, in mosquito generations. ${\delta_{b}}^{i}$ is the basal decay rate of insecticide i. $\omega_{0}^{i}$ is efficacy of insecticide i at τ=0.

In stage 2 (red part of Fig 3), the insecticide has been deployed beyond a longevity threshold, $\tau_{b}^{i}$. After this threshold there is a change in the decay rate and insecticide efficacy is calculated:


$${\omega_{\tau }^{i}=\;(\omega }_{0}^{i}*\;exp(-{\tau_{b}^{i}}^{2}*\;{\delta_{b}}^{i}))*\;\exp{\left(-(\tau -\;\tau_{b}^{i}\right)}^{2}*\;{\delta_{r}}^{i}),\;where\;\tau_{b}^{i}<\;\tau$$ (2d(ii))

Where $\exp\left(-{\tau_{b}^{i}}^{2}*\;{\delta_{b}}^{i}\right)$ is the insecticide decay occurring before the rapid degradation, and $\exp{\left(-(\tau -\;\tau_{b}^{i}\right)}^{2}*\;{\delta_{r}}^{i})$ is the rapid decay occurring after the longevity threshold has been exceeded ($\tau_{b}^{i}$). ${\delta_{b}}^{i}$ is the decay rate during $\tau_{b}$ generations prior to the longevity threshold and ${\delta_{r}}^{i}$ the decay rate after the longevity threshold ($\tau_{b}^{i}$).

**Fig 4:**
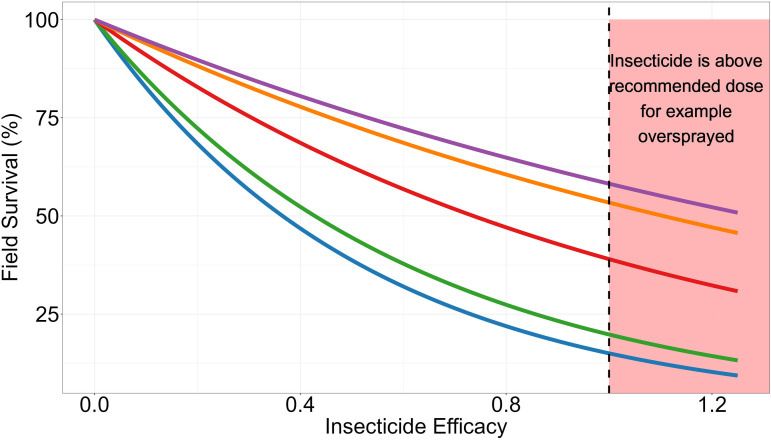
Impact of insecticide efficacy on field survival at given polygenic resistance scores. This plot displays the field survival of mosquitoes which encounter an insecticide of a particular efficacy, calculated using Equation 2b(i). The colour of the line corresponds to the polygenic resistance score. Blue = 0, Green = 10, Red = 100, Orange = 900, Purple = 8100; corresponding to 0, 2, 10, 50 and 80% bioassay survivals. The black vertical line indicates the insecticide is at its manufacturers recommended concentration for 100% efficacy, which is 1. The red background indicates the insecticide is above the manufactures recommended dosing/concentration as may occur, for example, with over-spraying when performing indoor residual spraying.

The initial deployed insecticide efficacy is set by specifying $\omega_{0}^{i}$. This therefore allows for over ($\omega_{0}^{i}$>1) and under ($\omega_{0}^{i}<1$) spraying which can occur during IRS implementation. $\omega_{0}^{i}<1$ can also be used for simulating reduced-dose mixtures i.e., where constituent insecticides in a mixture are present at reduced concentrations so are below the manufacturer’s recommended monotherapy dose. $\omega_{0}^{i}$ is defined as the insecticide’s ability to kill fully susceptible mosquitoes (i.e., mosquitoes with $z_{I}\leq 0$), Values of $\omega_{0}^{i}$ above 1 mean the insecticide can effectively kill mosquitoes at higher resistance levels ($z_{I}>0$) under field condition (see Fig 4).

Allowing a two-step decay process increases the possible decay profiles. This two-step decay profile is a composite of both insecticide concentration decay and physical net degradation. The insecticidal ability of ITNs measured in wire ball assays [25] does not account for damage to the net (and therefore the probability of reduced contact times). This two-step decay process captures the impact of reduced efficacy as a result of a decay in the efficacy of the insecticide and the physical decay of the net [26]. A single-step linear decay occurs if $\tau_{b}^{i}$ ≥ deployment interval or if ${\delta_{b}}^{i}$ = ${\delta_{r}}^{i}$.

#### Methods Section 1.3. Converting bioassay survival to/from field survival as a function of insecticide efficacy.

We previously modelled field survival to the insecticide ($K_{i}^{F}$) as a linear relationship with bioassay survival ($K_{i}^{B}$) (Equation 2b) [11]. This relationship has been updated to account for insecticide efficacy ($\omega_{\tau }^{i}$).


$$K_{i}^{F}=\;{{(\phi }_{1}K_{i}^{B}+\phi_{2})}^{\omega_{\tau }^{i}}$$ (2b(i))

Where $\varphi_{1}$ and $\varphi_{2}$ are the regression coefficient and intercept of the linear model respectively. $\omega_{\tau }^{i}$ is calculated in Equations 2d(i) and 2d(ii). When $\omega_{\tau }^{i}=0$, the insecticide has decayed such that survival is 100%. The relationship between PRS, insecticide efficacy and field survival is demonstrated in Fig 4.

#### Methods Section 1.4: Standard deviation of the Polygenic Resistance Score.

An important question for a Normally distributed trait, such as PRS, is its phenotypic standard deviation ($\sigma_{I}$). There are two distinct options. First, $\sigma_{I}$ remains constant over the course of IR selection. Second, $\sigma_{I}\;$changes with respect to the current value of ${\overset{―}{z}}_{I}$ (for example the coefficient of variation SD/mean may remain constant such that the arithmetic value of the standard deviation depends on the mean). Varying $\sigma_{I}$ with ${\overset{―}{z}}_{I}$ becomes necessary when considering high IR level scenarios.

#### Option 1: $\sigma_{I}$ is fixed.

Mathematical models of IRM have focused mainly on the deployment of insecticides to which there is initially little IR [11,17,20,21,27] to identify strategies which maintain low levels of IR. For the “polysmooth” and “polytruncate”, this would limit ${\overset{―}{z}}_{I}$ to 100 (10% bioassay survival), our default definition of a “failing insecticide” [11,22]. Keeping $\sigma_{I}\;$ fixed when ${\overset{―}{z}}_{I\;}$ remains low is intuitive as this additionally allows simple exploration of having a highly versus lowly variable starting populations versus a less variable population. Calibrating models (e.g., the exposure scaling factor (β), see S2 File) in Equation 3c to expected timescales (see [11]) is simpler using fixed $\sigma_{I}$.

#### Option 2: $\sigma_{I}$ varies with ${\overset{―}{z}}_{I\;}$.

Not all changes in PRS correspond to the same unit change in bioassay survival, as the relationship between PRS and bioassay survival is curved (Fig 2), suggesting $\sigma_{I}$ may need to increase with ${\overset{―}{z}}_{I}$. If $\sigma_{I}$ = 30, this is a large when ${\overset{―}{z}}_{I}\;$= 50, but small when ${\overset{―}{z}}_{I}$ is 3600. $\sigma_{I}$ needs to change with respect to ${\overset{―}{z}}_{I}$ to account for this. A linear model of $\sigma_{I}\sim {\overset{―}{z}}_{I}$ was used to estimate this, using WHO tube bioassay results from the field and the standard deviation of those results (S3 File).


$$\sigma_{I}=\;\phi_{3}{\overset{―}{z}}_{I}+\phi_{4}\;$$ (2e)

$\phi_{3}$ and $\phi_{4}$ are the regression coefficient and regression intercept respectively of a linear model. If the intervention site and refugia have different ${\overset{―}{z}}_{I}$, then site specific $\sigma_{I}$ are calculated:


$$\sigma_{I}^{Int}=\;\phi_{3}{\overset{―}{z}}_{I}^{Int}+\phi_{4}\;$$ (2e(Int))

$$\sigma_{I}^{Ref}=\;\phi_{3}{\overset{―}{z}}_{I}^{Ref}+\phi_{4}\;$$ (2e(Ref))

### Methods section 2: Biology of insecticide selection.

#### Methods Section 2.1: Calculating the Response to Insecticide Selection.

Deploying insecticides leads to selection which increases ${\overset{―}{z}}_{I}$ over successive generations. Fitness costs may reduce ${\overset{―}{z}}_{I\;}$ over successive generations [28]. The overall selection differential ($S_{I}^{S\varphi }$) therefore depends on both insecticide selection ($S_{I}^{S}$) and fitness costs $(S_{I}^{\varphi }$). The S superscript indicates insecticide selection and the φ superscript indicates fitness costs. Male (\mars) and female (\venus) mosquitoes have different behaviours (which alter their levels of exposure to insecticides) and physiologies so their selection differentials are calculated separately.


$$S_{I}^{S\varphi \text{\textbackslash{}venus}}=S_{I}^{S\text{\textbackslash{}venus}}+S_{I}^{\varphi \text{\textbackslash{}venus}}\;$$ (3b(♀))

$$S_{I}^{S\varphi \text{\textbackslash{}mars}}=\;S_{I}^{S\text{\textbackslash{}mars}}+S_{I}^{\varphi \text{\textbackslash{}mars}}\;$$ (3b(♂))

The overall selection differentials are used in the sex-specific Breeder’s equation [10] to calculate the response:


$$R_{I}^{S\varphi }=h_{I}^{2}\;\frac{\left(S_{I}^{S\varphi \text{\textbackslash{}venus}}+S_{I}^{S\varphi \text{\textbackslash{}mars}}\right)}{2}\beta \;$$ (3c)

$R_{I}^{S\varphi }$ is the response in trait I (i.e., resistance to insecticide i), $h_{I}^{2}$ is the heritability of trait I. β is the exposure scaling factor and is used to calibrate simulations to defined timescales. When the insecticide is not present (i.e., in refugia, or not currently deployed in the intervention site), the sex-specific Breeder’s equation includes only fitness cost selection differentials ($S_{I}^{\varphi \text{\textbackslash{}venus}}$ and $S_{I}^{\varphi \text{\textbackslash{}mars}}$), and the response is therefore $R_{I}^{\varphi }$.

#### Methods Section 2.2 Calculating Insecticide Selection Differentials.

**Fig 5:**
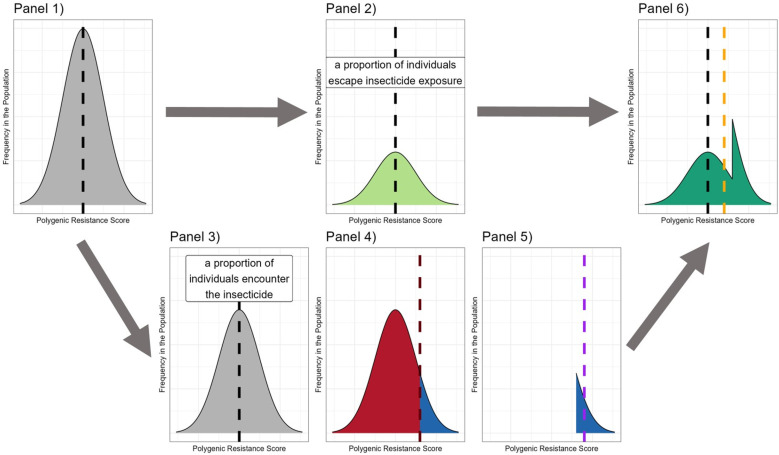
Compartmental diagram of truncation selection process for a single generation. The “polytruncate” model implements truncation selection, and determines which equations are used to calculate each stage of the selection process. Note this process is calculated separately for males and females. **Panel 1**. The mosquito population emerges, with a PRS that is Normally distributed with mean ${\overset{―}{z}}_{I}$ (black line) and standard deviation $\sigma_{i}$. At emergence there are a total of $N^{T}$ individuals. **Panel 2**. A proportion (males: 1−mx and females: 1−x) avoids the insecticide and insecticide selection; the mean is therefore unchanged at ${\overset{―}{z}}_{I}$, there will be $N^{u}$ of these individuals (Equation 4d). **Panel 3**. A proportion do encounter the insecticide (males: mx and females: x) (Equation 5d). **Panel 4**. These individuals are selected by truncation selection (Equation 5c). Individuals with a PRS less than the defined threshold (red line) are killed (red area). Individuals with a PRS above the threshold survive (blue area). **Panel 5**. Only the most resistant individuals in the population will have survived the insecticide encounter, these individuals have a mean of ${\overset{―}{z}}_{I}^{E}$ (purple line) (Equation 5c), and there are $N_{i}^{E}$ of these individuals (Equation 5d). **Panel 6.** The unexposed group (Panel 2) and the exposed survivors (Panel 5) form the final breeding parental population. They have a mean of ${\overset{―}{z}}_{I}^{S}$ (orange line) (Equation 4b), which would be expected to be higher than the original population mean (black line). The insecticide selection differential is the difference between the orange and black lines (Equation 3b).

**Fig 6:**
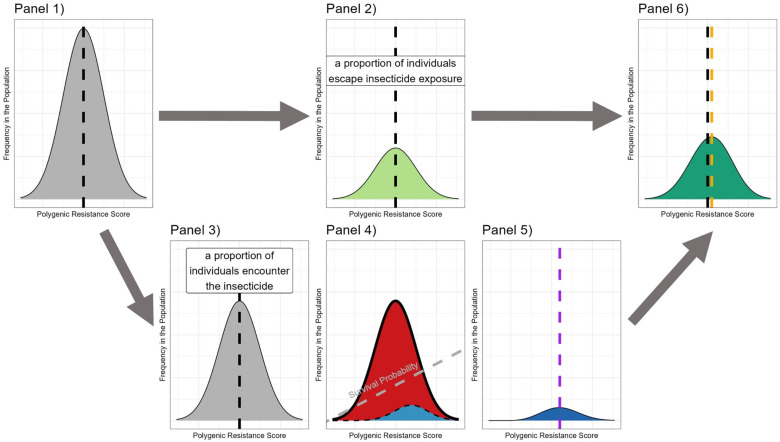
Compartmental diagram of a probabilistic selection process for a single generation. This is implemented in “polysmooth”. Panels 1-3 and 6 have same description as Fig 5. Where the two models diverge is the calculation of who survives/dies insecticide exposure. **Panel 4:** The probability of surviving insecticide encounter depends on an individual’s polygenic resistance score i.e., individuals with a higher polygenic resistance score have a higher probability **Panel 5.** The mosquitoes surviving exposure will have a higher mean polygenic resistance score (purple line (Equation 6c) and there are $N_{i}^{E}$ individuals (Equation 6b).


We calculate the insecticide selection differential ($S_{I}^{S}$) dynamically each generation based on both the current level of IR and insecticide efficacy (“polytruncate”: Fig 5 and “polysmooth”: Fig 6). This differs from our previous work which assumed selection pressure was constant [11].:

$$S_{I}^{S\text{\textbackslash{}venus}}=\;{\overset{―}{z}}_{I}^{P\text{\textbackslash{}venus}}-\;{\overset{―}{z}}_{I}^{\text{\textbackslash{}venus}}$$ (4a(♀))

$$S_{I}^{S\text{\textbackslash{}mars}}=\;{\overset{―}{z}}_{I}^{P\text{\textbackslash{}mars}}-\;{\overset{―}{z}}_{I}^{\text{\textbackslash{}mars}}$$ (4a(♂))

${\overset{―}{z}}_{I}^{\text{\textbackslash{}venus}}\;$and ${\overset{―}{z}}_{I}^{\text{\textbackslash{}mars}}$ are the mean PRS prior to insecticide selection assuming both sexes have the same level of IR at birth i.e., ${\overset{―}{z}}_{I}^{\text{\textbackslash{}venus}}$ = ${\overset{―}{z}}_{I}^{\text{\textbackslash{}mars}}$. The “P” superscript indicates the final breeding parental population. The level of IR for males is generally unknown, as bioassays are typically only performed on female mosquitoes. ${\overset{―}{z}}_{I}^{P\text{\textbackslash{}venus}}$ and ${\overset{―}{z}}_{I}^{P\text{\textbackslash{}mars}}$are the means of the final breeding parental population, calculated each generation.

${\overset{―}{z}}_{I}^{P\text{\textbackslash{}venus}}$and ${\overset{―}{z}}_{I}^{P\text{\textbackslash{}mars}}$ are calculated from those surviving insecticide exposure and those which avoided the insecticide:


$${\overset{―}{z}}_{I}^{P\text{\textbackslash{}venus}}=\;\frac{{N_{i}^{E\text{\textbackslash{}venus}}\overset{―}{z}}_{I}^{E\text{\textbackslash{}venus}}+\;N^{u\text{\textbackslash{}venus}}{\overset{―}{z}}_{I}^{\text{\textbackslash{}venus}}}{N^{P\text{\textbackslash{}venus}}}$$ (4b(♀))

$${\overset{―}{z}}_{I}^{P\text{\textbackslash{}mars}}=\;\frac{{N_{i}^{E\text{\textbackslash{}mars}}\overset{―}{z}}_{I}^{E\text{\textbackslash{}mars}}+\;N^{u\text{\textbackslash{}mars}}{\overset{―}{z}}_{I}^{\text{\textbackslash{}mars}}}{N^{P\text{\textbackslash{}mars}}}$$ (4b(♂))

The number surviving the insecticide exposure is $N_{i}^{E\text{\textbackslash{}venus}}$ and $N_{i}^{E\text{\textbackslash{}mars}}$, having a mean PRS of ${\overset{―}{z}}_{I}^{E\text{\textbackslash{}venus}}$ and ${\overset{―}{z}}_{I}^{E\text{\textbackslash{}mars}}$. The “E” superscript indicates they underwent insecticide exposure.

$N^{u\text{\textbackslash{}venus}}$and $N^{u\text{\textbackslash{}mars}}$ are the number which avoided the insecticide(s) and survive to become parents. The “u” superscript indicates they were not exposed to the insecticide(s). By not having insecticide selection these individuals have an unchanged mean PRS of ${\overset{―}{z}}_{I}^{\text{\textbackslash{}venus}}$ and ${\overset{―}{z}}_{I}^{\text{\textbackslash{}mars}}$.

$N^{P\text{\textbackslash{}venus}}$and $N^{P\text{\textbackslash{}mars}}$ are the total number of breeding parents consisting of those surviving exposure and those avoiding exposure:


$$N^{P\text{\textbackslash{}venus}}=\;N_{i}^{E\text{\textbackslash{}venus}}+\;N^{u\text{\textbackslash{}venus}}$$ (4c(♀))

$$N^{P\text{\textbackslash{}mars}}=\;N_{i}^{E\text{\textbackslash{}mars}}+\;N^{u\text{\textbackslash{}mars}}$$ (4c(♂))

$N^{u\text{\textbackslash{}venus}}$and $N^{u\text{\textbackslash{}mars}}$ is calculated as:


$$N^{u\text{\textbackslash{}venus}}\;=\;N^{T\text{\textbackslash{}venus}}(1-x)$$ (4d(♀))

$$N^{u\text{\textbackslash{}mars}}\;=\;N^{T\text{\textbackslash{}mars}}(1-mx)$$ (4d(♂))

*x* is the proportion of females exposed to the insecticide and m is the male exposure as a proportion of female exposure (generally m<1 as male behaviour make them less likely to encounter insecticides).

The question is how to calculate ${\overset{―}{z}}_{I}^{E\text{\textbackslash{}venus}}$ and ${\overset{―}{z}}_{I}^{E\text{\textbackslash{}mars}}$ in Equations 4b. This can be calculated directly for probabilistic selection. For truncation selection, ${\overset{―}{z}}_{I}^{E\text{\textbackslash{}venus}}$ and ${\overset{―}{z}}_{I}^{E\text{\textbackslash{}mars}}$ can be obtained by first calculating the selection differentials between the exposed survivors and the mean population ($S_{I}^{E\text{\textbackslash{}venus}}$ and $S_{I}^{E\text{\textbackslash{}mars}}$):


$${\overset{―}{z}}_{I}^{E\text{\textbackslash{}venus}}=\;{\overset{―}{z}}_{I}^{\text{\textbackslash{}venus}}+S_{I}^{E\text{\textbackslash{}venus}}$$ (4h(♀))

$${\overset{―}{z}}_{I}^{E\text{\textbackslash{}mars}}=\;{\overset{―}{z}}_{I}^{\text{\textbackslash{}mars}}+\;S_{I}^{E\text{\textbackslash{}mars}}$$ (4h(♂))

#### Methods Section 2.3: Calculating Insecticide Selection Differentials by truncation selection (“polytruncate”).

In truncation selection only the most resistant individuals survive insecticide exposure and is implemented using the equation for the intensity of selection [10]:


$$S_{I}^{E}=\;\sigma_{I\;}\frac{\phi \left({{\overset{―}{z}}_{I}}_{[1-{\overset{―}{K}}_{i}^{F}]}\right)}{{\overset{―}{K}}_{i}^{F}}$$ (5a)

The value ${z_{I}}_{[1-{\overset{―}{K}}_{i}^{F}]}$ is the PRS where ${\overset{―}{K}}_{I}^{F}$ is the mean survival of the population. $\varphi (z_{I})$ is the unit Normal density distribution function:


$$\phi \left(z_{I}\right)=\;\frac{1}{\sqrt{2\pi }}\exp^{-z_{I}^{2}/2}$$ (5b)

${\overset{―}{z}}_{I}^{E\text{\textbackslash{}venus}}$and ${\overset{―}{z}}_{I}^{E\text{\textbackslash{}mars}}$ are then calculated:


$${\overset{―}{z}}_{I}^{E\text{\textbackslash{}venus}}=\left(\sigma_{I\;}\frac{\phi \left({z_{I}}_{[1-{\overset{―}{K}}_{i}^{F}]}\right)}{{\overset{―}{K}}_{i}^{F}}\right)\;+\;{\overset{―}{z}}_{I}^{\text{\textbackslash{}venus}}$$ (5c(♀))

$${\overset{―}{z}}_{I}^{E\text{\textbackslash{}mars}}=\left(\sigma_{I\;}\frac{\phi \left({z_{I}}_{[1-{\overset{―}{K}}_{i}^{F}]}\right)}{{\overset{―}{K}}_{i}^{F}}\;\right)+\;{\overset{―}{z}}_{I}^{\text{\textbackslash{}mars}}$$ (5c(♂))

The number of females and males which survive is the number exposed ($xN^{T\text{\textbackslash{}venus}}$ for females, and $xmN^{T\text{\textbackslash{}mars}}$ for males) multiplied by proportion surviving (${\overset{―}{K}}_{i}^{F}$):


$$N_{i}^{E\text{\textbackslash{}venus}}=xN^{T\text{\textbackslash{}venus}}{\overset{―}{K}}_{i}^{F}$$ (5d(♀))

$$N_{i}^{E\text{\textbackslash{}mars}}=xmN^{T\text{\textbackslash{}mars}}{\overset{―}{K}}_{i}^{F}$$ (5d(♀))

Extending for mixtures, where mosquitoes must simultaneously survive the encounter to both insecticides (i and j):


$$N_{\text{ij}}^{E\text{\textbackslash{}venus}}=xN_{\text{\textbackslash{}venus}}^{T}{\overset{―}{K}}_{i}^{F}{\overset{―}{K}}_{j}^{F}$$ (5d(i)(♀))

$$N_{\text{ij}}^{E\text{\textbackslash{}mars}}=xmN_{\text{\textbackslash{}mars}}^{T}{\overset{―}{K}}_{i}^{F}{\overset{―}{K}}_{j}^{F}$$ (5d(i)(♂))

${\overset{―}{K}}_{j}^{F}$is the mean survival to insecticide j, calculated from Equation 2b(i) depending on ${\overset{―}{z}}_{J}$ and $\omega_{\tau }^{i}$.

${\overset{―}{z}}_{I}^{E\text{\textbackslash{}venus}}$, ${\overset{―}{z}}_{I}^{E\text{\textbackslash{}mars}}$, $N_{i}^{E\text{\textbackslash{}venus}}$and $N_{i}^{E\text{\textbackslash{}mars}}$ are returned to Equations 4b. If a mixture was deployed $N_{ij}^{E\text{\textbackslash{}venus}}$ and $N_{ij}^{E\text{\textbackslash{}mars}}\;$replaces $N_{i}^{E\text{\textbackslash{}venus}}$and $N_{i}^{E\text{\textbackslash{}mars}}$ in Equations 4b. Solving equations 4b gives ${\overset{―}{z}}_{I}^{P\text{\textbackslash{}venus}}$ and ${\overset{―}{z}}_{I}^{P\text{\textbackslash{}mars}}$, which is required to calculate $S_{I}^{S\text{\textbackslash{}venus}}$and $S_{I}^{S\text{\textbackslash{}mars}}$ and gives the overall selection differentials. These are then used in the sex-specific Breeder’s Equations (Equation 3c) to calculate the overall response which can be passed through to Equation 8 to obtain the mean PRS of the next generation.

#### Methods Section 2.4: Calculating Insecticide Selection Differentials by probabilistic selection (“polysmooth”).

In “polysmooth”, survival is modelled as a probabilistic process. The PRS is a continuous, quantitative trait, so the values of $z_{I}$ are “binned” into a large number of discrete classes with a corresponding frequency ($F_{z_{I}}$) (Fig A in S1 File). For each sex and resistance trait the model tracks two vectors, one which contain the discrete “binned” values of $z_{I}$ and a corresponding vector containing their frequencies ($F_{z_{I}}$). Tracking changes in the distributions of the “binned” values allows for simple computation.

$F_{z_{I}}$is the frequency of individuals with PRS $z_{I}$, calculated using the unit Normal density distribution function:


$$F_{z_{I}}=\;\frac{1}{\sigma_{I}\sqrt{2\pi }}e^{-\frac{1}{2}(\frac{z_{I}-\;{\overset{―}{z}}_{I}}{\sigma_{I}}{)}^{2}}$$ (4g)

Females ($F_{z_{I}^{\text{\textbackslash{}venus}}}$) and males ($F_{z_{I}^{\text{\textbackslash{}mars}}}$) are each half of $F_{z_{I}}$ at hatching.

The total population size prior to insecticide selection is the sum all $F_{z_{I}}$ calculated as:


$$N^{T}=\;\sum_{z_{I}=\;-\infty }^{\infty }F_{z_{I}}$$ (4f)

$N_{\text{\textbackslash{}venus}}^{T}$and $N_{\text{\textbackslash{}mars}}^{T}$ are calculated as half of the total population size before any selection has occurred, assuming the ratio at hatching of males to females is 1:1.

$F_{z_{I}^{E\text{\textbackslash{}venus}}}$ and $F_{z_{I}^{E\text{\textbackslash{}mars}}}$ are the frequencies after insecticide exposure and selection, with “E” indicating these mosquitoes survived insecticide exposure. $F_{z_{I}^{E\text{\textbackslash{}venus}}}$ is calculated as $F_{z_{I}^{\text{\textbackslash{}venus}}}$ multiplied by the probability of insecticide encounter (x) and the probability of surviving the insecticide exposure ($K_{i}^{F}$) given $z_{I}^{\text{\textbackslash{}venus}}$ and $\omega_{\tau }^{i}$:


$$F_{z_{I}^{E\text{\textbackslash{}venus}}}=\;F_{z_{I}^{\text{\textbackslash{}venus}}}\;K_{i}^{F}x$$ (6a(♀))

For males, $K_{i}^{F}$ is dependent on $z_{I}^{\text{\textbackslash{}mars}}$, and the probability of insecticide encounter is xm:


$$F_{z_{I}^{E\text{\textbackslash{}mars}}}=\;F_{z_{I}^{\text{\textbackslash{}mars}}}K_{i}^{F}xm$$ (6a(♂))

When investigating mixtures, mosquitoes simultaneously encounter both insecticides, and therefore the mosquitoes must also survive the encounter with insecticide j:


$$F_{z_{I}^{E\text{\textbackslash{}venus}}}=\;F_{z_{I}^{\text{\textbackslash{}venus}}}K_{i}^{F}{\overset{―}{K}}_{j}^{F}x$$ (6a(i)(♀))

$$F_{z_{I}^{E\text{\textbackslash{}mars}}}=\;F_{z_{I}^{\text{\textbackslash{}mars}}}K_{i}^{F}{\overset{―}{K}}_{j}^{F}mx$$ (6a(i)(♂))

${\overset{―}{K}}_{j}^{F}$is the mean population survival to insecticide j and is calculated from Equation 2b(i) and is dependent on the mean resistance to insecticide j (${\overset{―}{z}}_{J}$) and insecticide j efficacy ($\omega_{\tau }^{j}$). The model does not allow for individual level genetic correlation but includes population level cross resistance (see Equations 8b, 8c and 8d later).

The total number surviving insecticide exposure ($N_{i}^{E\text{\textbackslash{}venus}}$ and $N_{i}^{E\text{\textbackslash{}mars}}$), is therefore the sum of the frequencies of the exposed survivors ($F_{z_{I}^{E\text{\textbackslash{}venus}}}$ and $F_{z_{I}^{E\text{\textbackslash{}mars}}}$).


$$N_{i}^{E\text{\textbackslash{}venus}}=\;\sum_{z_{I}^{\text{\textbackslash{}venus}}=\;-\infty }^{\infty }F_{z_{I}^{E\text{\textbackslash{}venus}}}$$ (6b(♀))

$$N_{i}^{E\text{\textbackslash{}mars}}=\;\sum_{z_{I}^{\text{\textbackslash{}mars}}=\;-\infty }^{\infty }F_{z_{I}^{E\text{\textbackslash{}mars}}}$$ (6b(♂))

The mean PRS of the exposed survivors is then calculated:


$${\overset{―}{z}}_{I}^{E\text{\textbackslash{}venus}}=\left(\sum_{z_{I}^{E\text{\textbackslash{}venus}}=\;-\infty }^{\infty }F_{z_{I}^{E\text{\textbackslash{}venus}}}z_{I}^{\text{\textbackslash{}venus}}\right)/N_{i}^{E\text{\textbackslash{}venus}}$$ (6c(♀))

$${\overset{―}{z}}_{I}^{E\text{\textbackslash{}mars}}=\;\left(\sum_{z_{I}^{E\text{\textbackslash{}mars}}=\;-\infty }^{\infty }F_{z_{I}^{E\text{\textbackslash{}mars}}}z_{I}^{\text{\textbackslash{}mars}}\right)/N_{i}^{E\text{\textbackslash{}mars}}$$ (6c(♂))

For mixtures $N_{i}^{E\text{\textbackslash{}venus}}$ and $N_{i}^{E\text{\textbackslash{}mars}}$ are replaced by $N_{ij}^{E\text{\textbackslash{}venus}}$ and $N_{ij}^{E\text{\textbackslash{}mars}}$ in Equations 4b, 6b and 6c). ${\overset{―}{z}}_{I}^{E\text{\textbackslash{}venus}}$, ${\overset{―}{z}}_{I}^{E\text{\textbackslash{}mars}}$, $N_{i}^{E\text{\textbackslash{}venus}}$and $N_{i}^{E\text{\textbackslash{}mars}}$ are returned to Equations 4b. Solving equations 4b gives ${\overset{―}{z}}_{I}^{P\text{\textbackslash{}venus}}$ and ${\overset{―}{z}}_{I}^{P\text{\textbackslash{}mars}}$, which is required to calculate the insecticide selection differential ($S_{I}^{S\text{\textbackslash{}venus}}$and $S_{I}^{S\text{\textbackslash{}mars}}$). This can be combined with any fitness costs to give the overall selection differentials. These are then used in the sex-specific Breeder’s Equations (Equation 3c) to calculate the overall response.

#### Methods Section 2.5: Calculating the fitness cost selection differential.

Fitness costs can be associated with IR [29]. Fitness costs impact many aspects of mosquito life-history traits including “natural” survival, growth rates, mating and fecundity [28]. The model does not include the explicit mechanism of the fitness costs. We assume all fitness costs work to reduce the mean PRS over time in the population. We do this by including a fitness cost specific selection differential (Equation 3b). This captures of the effect of fitness costs without explicitly including the mechanism.

There are two intuitive ways to calculate the fitness cost selection differentials ($S_{I}^{\varphi \text{\textbackslash{}venus}}$and $S_{I}^{\varphi \text{\textbackslash{}mars}}$). Option 1 assumes the fitness cost selection differential remains a fixed constant over the course of selection, and is used when $\sigma_{I}$ is a fixed value. Option 2 assumes that $S_{I}^{\varphi \text{\textbackslash{}venus}}$and $S_{I}^{\varphi \text{\textbackslash{}mars}}$ vary with $\sigma_{I}$ and is used when $\sigma_{I}$ changes with ${\overset{―}{z}}_{I}$. Parameter value estimation for Option 1 and 2 is detailed in the S4 File.

#### Option 1: Fitness selection differentials remain fixed.

If $\sigma_{I}$ remains fixed, then it seems intuitive for $S_{I}^{\varphi }$ and $S_{I}^{\varphi \text{\textbackslash{}mars}}$ to remain fixed throughout the simulations. This is because the difference in PRS between the most resistant individuals and least resistant remains constant, and therefore the relative fitness difference between the most and least resistant individuals remains constant.

#### Option 2: Fitness selection differentials vary with $\sigma_{I}$ and ${\overset{―}{z}}_{I}$.

If $\sigma_{I}$ varies with ${\overset{―}{z}}_{I}$ the difference in PRS between the most and least resistant individuals in the population varies. We therefore allow the fitness costs to be a fixed proportion of the standard deviation, allowing the fitness costs selection differential to increase with ${\overset{―}{z}}_{I}$ and therefore increasing $\sigma_{I}$.


$$S_{I}^{\varphi }=\varphi \sigma_{I}\;$$ (7a)

Fitness costs may be different in males and females, so we separately calculate the sex-specific fitness cost selection differentials.


$$S_{I}^{\varphi \text{\textbackslash{}venus}}=\varphi^{\text{\textbackslash{}venus}}\sigma_{I}\;$$ 7b(♀)

$$\;S_{I}^{\varphi \text{\textbackslash{}mars}}=\varphi^{\text{\textbackslash{}mars}}\sigma_{I}\;$$ 7b(♂)

Manually inputting the fitness selection differentials (option 1 or option 2), may result in IR never spreading in some simulations i.e., if the fitness costs are greater than insecticide selection ($S_{I}^{\varphi \text{\textbackslash{}venus}}$ and $S_{I}^{\varphi \text{\textbackslash{}mars}}$ are greater than $S_{I}^{S\text{\textbackslash{}venus}}$ and $S_{I}^{S\text{\textbackslash{}mars}}$). This would occur in situations where insecticide selection is low (e.g., low coverage and/or low exposure) such that even in the absence of any fitness cost, IR would be very slow to build up. In situations where IR does not spread, or spreads slowly, there is little need for IRM.

#### Methods Section 2.6: Between Generation Changes in Resistance.

Simulations track two operational sites, the intervention and refugia. The refugia is the part of the landscape where insecticides are never deployed. For convenience we assume a single intervention site and a single refugia which can be viewed as an amalgamation of all intervention sites together and all refugia together with a global migration between the sites (see section 2.7 below). Insecticide deployments and decision-making are only made in the intervention site.

For each insecticide the corresponding PRS is tracked in the intervention site (${\overset{―}{z}}_{I\;}^{Int}$) and refugia (${\overset{―}{z}}_{I\;}^{Ref}$) for each generation. When insecticide i is deployed in monotherapy the response is a result of both insecticide selection and fitness costs.


$${\overset{―}{z}}_{I\;}^{Int^{\prime }}=\;{\overset{―}{z}}_{I\;}^{Int}+R_{I}^{S\varphi }$$ (8a)

If insecticide i is not deployed and insecticide γ is deployed, there may be indirect selection on trait *I* from insecticide γ from a genetic correlation between trait Γ and trait *I*, termed $\alpha_{\Gamma I}$ [11]. This effect is referred to as both “cross selection” or “cross resistance” and are often used interchangeably; here we use cross resistance.


$${\overset{―}{z}}_{I\;}^{Int^{\prime }}=\;{\overset{―}{z}}_{I\;}^{Int}+R_{I}^{\varphi }+\;\left(\alpha_{\Gamma I}\;R_{\Gamma }^{S\varphi }\right)$$ (8b)

Where γ∈{j,k,l…} is the set of insecticides that may be deployed other than insecticide i and Γ∈{J,K,L…} is the set of PRS values to the corresponding insecticides.

For insecticide mixture deployments when insecticide i is deployed with j there may be cross resistance between both insecticides:


$${\overset{―}{z}}_{I}^{{Int}^{\prime }}=\;{\overset{―}{z}}_{I}^{Int\;}+R_{I}^{S\varphi }+\left(R_{\Gamma }^{S\varphi }\alpha_{\Gamma I}\right)$$ (8c)

If insecticide i is not deployed, but a mixture of j and k is deployed, there may be indirect selection on trait I from both insecticide j and insecticide k.


$${{\overset{―}{z}}_{I}^{Int}}^{\prime }=\;{(\overset{―}{z}}_{I}^{Int}+\;R_{I}^{\varphi })+\;(R_{J}^{S\varphi }\alpha_{JI})\;+\;{(R}_{K}^{S\varphi }\alpha_{KI})\;$$ (8d)

In the refugia, where insecticides are not deployed, the response ($R_{I}^{\varphi })$ is only from fitness costs


$${\overset{―}{z}}_{I\;}^{Ref^{\prime }}=\;{\overset{―}{z}}_{I\;}^{Ref}+R_{I}^{\varphi }\;$$ (8e)

#### Methods Section 2.7 Mosquito Dispersal.

After insecticide selection and mating, mosquitoes can migrate between the intervention site and refugia. Dispersal results in gene flow that may alter the dynamics of how resistance evolves so must be an option in the modelling (see [30] for details). Dispersal is described as follows:


$$r_{Ref}=(1-Ctheta$$ (9a)

$$r_{Int}=\theta C$$ (9b)

Where C is the coverage of the insecticide and corresponds to the proportion of the mosquito population in the intervention site. θ is the proportion of females dispersing. $r_{Int}$ is the number of females migrating from the intervention site to the refugia. $r_{Ref}$ is the number of females migrating from the refugia to the intervention site. Note that mating occurs before migration so we do need to track male movement

The mean PRS of eggs laid by females in the intervention site is therefore:


$${\overset{―}{z}}_{I}^{Int^{\prime \prime }}=\;\left({\overset{―}{z}}_{I\;}^{Int^{\prime }}*\;(1-r_{Int})\right)+\;{(\overset{―}{z}}_{I\;}^{Ref^{\prime }}*\;r_{Ref})$$ (9c)

The mean PRS of the eggs laid by females in the refugia is:


$${\overset{―}{z}}_{I}^{Ref^{\prime \prime }}=\;\left({\overset{―}{z}}_{I\;}^{Ref^{\prime }}*\left(1-r_{Ref}\right)\right)+({\overset{―}{z}}_{I\;}^{Int^{\prime }}*\;r_{Int})$$ (9d)

Note the use of primes: single primes for PRS before dispersal, and double primes after dispersal: it is the double prime ${\overset{―}{z}}_{I\;}^{Int^{\prime \prime }}$ and ${\overset{―}{z}}_{I\;}^{Ref^{\prime \prime }}$ which become the mean PRS for the next generation in each site.

### Methods Section 3: Biology of Insecticide Selection over Multiple Gonotrophic Cycles

A key proposed benefit of micro-mosaic and combination deployments is they may constitute a “temporal mixture” where a mosquito surviving one insecticide is killed by a different insecticide in a subsequent gonotrophic cycle. Evaluation of micro-mosaics and combinations therefore requires incorporating insecticide selection over multiple cycles. This is technically complex but is justified by allowing the models to accurately simulate deployment of micro-mosaics and combinations. In addition it allows the estimation of female mosquito age profiles (a key factor in vector-borne disease transmission) and how they may change as a consequence of IRM.

Extending “polytruncate” to multiple cycles is problematic because it is unclear how to implement the truncation process after selection in the first cycle, when the PRS frequency distribution is no longer a Normal distribution. We therefore extend only the “polysmooth” model to include multiple cycles. This requires updating the various ways male mosquitoes could encounter the deployed insecticides (S5 File). We introduce the multi-cycle methodology first in the absence of dispersal, subsequently extending it to allow dispersal each cycle (S6 File).

**Fig 7:**
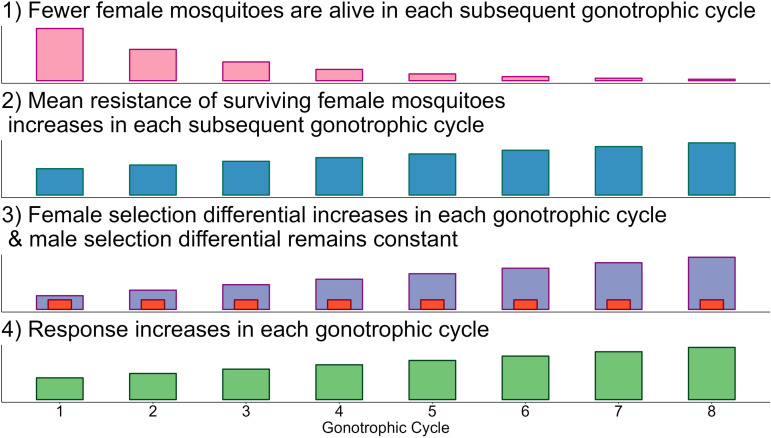
Conceptualisation of insecticide selection over multiple gonotrophic cycles. **1):** The number of females in each subsequent cycle decreases due to insecticide killing (e.g., $K_{i}^{F},\;K_{j}^{F}$) and natural survival (ρ). This results in fewer eggs being laid in subsequent cycles. **2):** As a result of insecticide selection, surviving females have a higher mean PRS than the previous cycle as only resistant individuals would be able to survive multiple insecticide encounters. **3):** An increasing mean PRS of females gives an increased female insecticide selection differential (height of blue bars). The male selection differential (height of red bars) remains constant as female mosquitoes mate only once. **4):** As the female selection differential increases, the response increases. Eggs laid by females in subsequent cycles produce more resistant individuals. The updated mean PRS for the next generation is the mean PRS of the eggs laid over all the cycles.

A conceptualisation of the selection process over multiple-cycles is shown in Fig 7. Importantly, the number of females laying eggs decreases each cycle due to both insecticide induced and “natural” mortality (Fig 7, Panel 1). After each round of insecticide selection in each cycle it is expected the mean level of resistance of the surviving females increases (Fig 7, Panel 2), increasing the female insecticide selection differential in each cycle. However, as females mate only once (in the first cycle), they carry the same male selection differential for all cycles (Fig 7, Panel 3). This results in increasing magnitudes of responses ($R_{I(G)}^{S\varphi }$, Equation 11b) such that in each subsequent cycle eggs will be laid from more resistant individuals (Fig 7, Panel 4). However fewer females are alive in later cycles so fewer of these eggs are laid (Fig 7, Panel 1). There is evidence that as mosquitoes age they become more suscepible to insecicides [31], including this effect would increase the importance of the first cycle so may be better approximated simply by assuming a single cycle as done previously. Note that insecticide decay occurs between generations and not between cycles.

#### Methods Section 3.1. Defining Coverages, Encounter Probabilities and Natural Survival.

**Fig 8:**
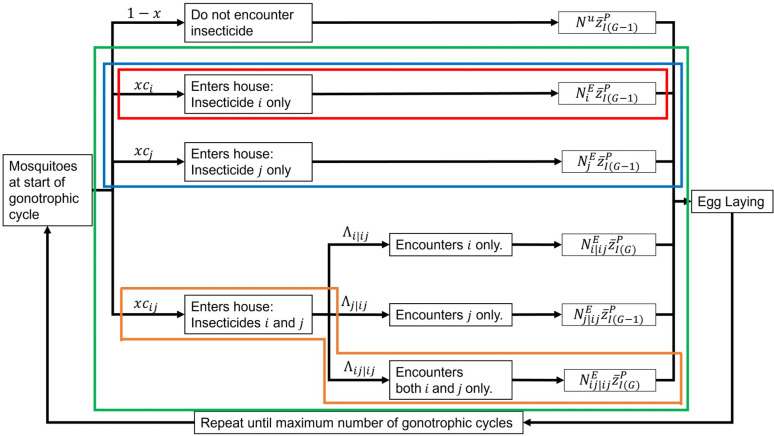
Compartmental diagram of the selection process over multiple gonotrophic cycles for IRM strategies. The framework of the multiple cycle model allows for multiple different strategies to be evaluated: Red indicates only a single insecticide is deployed, orange is a mixture, blue is a micro-mosaic, and green is a combination of ITN and IRS. These different strategies apply differing levels of selection producing different mean PRS values post insecticide selection and different number of females surviving each cycle.


Combinations and micro-mosaics change how mosquitoes encounter insecticides (Fig 8). Combinations have houses treated with two (or more) insecticides. For example, a house may receive both an ITN (containing insecticide i) and IRS (containing insecticide j). Or the household may receive an ITN with different insecticides on different panels (for example insecticide i on the top and insecticide j on the sides), so

$$c_{i}+\;c_{j}+\;c_{ij}=1$$ (10a)

Where $c_{i}$ is the proportion of treated houses with only insecticide i, $c_{j}$ is the proportion of treated houses with only insecticide j, $c_{ij}$ is the proportion of treated houses with both insecticide i and j. If $c_{ij}$ is set to 0, the strategy becomes is a micro-mosaic:


$$c_{i}+\;c_{j}=1$$ (10b)

If $c_{i}$ or $c_{j}$ is set to 1 then it is de-facto a monotherapy.

Mosquitoes entering a house with two insecticides i and j which are spatially separated, may encounter i only ($\Lambda_{i|ij}$), j only ($\Lambda_{j|ij}$) or both i and j ($\Lambda_{ij|ij}$). These parameters will be the result of mosquito behaviour (likely sex-specific), insecticidal properties and the distance between insecticides within the household. Mosquitoes encountering both insecticides ($\Lambda_{ij|ij}$) are treated as though encountering a mixture.


$$\Lambda_{i|ij}^{\text{\textbackslash{}mars}}+\Lambda_{j|ij}^{\text{\textbackslash{}mars}}\;+\;\Lambda_{ij|ij}^{\text{\textbackslash{}mars}}=1$$ (10c(♂))

$$\Lambda_{i|ij}^{\text{\textbackslash{}venus}}+\Lambda_{j|ij}^{\text{\textbackslash{}venus}}\;+\;\Lambda_{ij|ij}^{\text{\textbackslash{}venus}}=1$$ (10c(♀))

Note, if both $\Lambda_{ij|ij}^{\text{\textbackslash{}mars}}=1$ and $\Lambda_{ij|ij}^{\text{\textbackslash{}venus}}=1$, then the insecticides become a de-facto mixture. A compartmental description of the coverage-encounter process is given in Fig 8.

The multiple cycle model needs to account for natural (i.e., non-insecticidal) mortality and survival between cycles (ρ). We assume all females taking a bloodmeal lay eggs in that cycle (as assumed in single cycle models) and each individual female lays the same number of eggs in each cycle (although it would be possible to relax this assumption by adding a decay term, i.e., Equations 11c and 11d). The “natural” survival between cycles is:


$$\rho =d^{g}$$ (10d)

ρis the proportion of female mosquitoes at the end of the previous cycle starting the next cycle and is dependent on the “natural” daily survival probability (d) (i.e., mortality not caused by insecticides) and cycle length in days (g). “Natural” daily survival is assumed to be constant for all PRS values and for each cycle.

#### Methods Section 3.2. Calculating the Response to selection with multiple gonotrophic cycles.

The female insecticide selection differential for a cycle, ($S_{I\;(G)}^{S\text{\textbackslash{}venus}}$, where G=1,2,3 to $G_{\max}$) is the change in the mean PRS between parents in the current cycle (${\overset{―}{z}}_{I\;(G)}^{P\text{\textbackslash{}venus}}$) and at hatching (${\overset{―}{z}}_{I\;(G=0)}^{\text{\textbackslash{}venus}}$):


$$S_{I(G)}^{S\text{\textbackslash{}venus}}=\;{\overset{―}{z}}_{I\;(G)}^{P\text{\textbackslash{}venus}}-\;{\overset{―}{z}}_{I\;(G=0)}^{\text{\textbackslash{}venus}}$$ (11a(i))

The overall selection differential ($S_{I(G)}^{S\varphi \text{\textbackslash{}venus}}$) is from insecticide selection (subscript “S”) and fitness costs (subscript “φ”). We may expect most of the fitness costs to occur during larval development [32] and/or mating [33], therefore the fitness cost selection differential remains constant between cycles:


$$S_{I(G)}^{S\varphi \text{\textbackslash{}venus}}=S_{I(G)}^{S\text{\textbackslash{}venus}}+S_{I}^{\varphi \text{\textbackslash{}venus}}$$ (11a(ii))

There is evidence that as mosquitoes age they become less resistant [31], and may also produce fewer eggs. The fitness costs selection differential accounts for all aspects of life-history, for example larval growth rates, pupation rates and mating success, most of which will occur prior to, or shortly after, insecticide selection. However, the model is not explicit in how the fitness costs are implemented (doing so would require an additional population dynamics structure).

The response ($R_{I(G)}^{S\varphi }$) is calculated by updating the sex-specific Breeder’s equation (Equation 3c) for the current cycle:


$$R_{I(G)}^{S\varphi }=\;h_{I}^{2}\;\left(\frac{S_{I(G)}^{S\varphi \text{\textbackslash{}venus}}+S_{I}^{S\varphi \text{\textbackslash{}mars}}}{2}\;\right)\beta$$ (11b)

Surviving female mosquitoes progress through a maximum of $G_{\max}$ gonotrophic cycles. $G_{\max}$ is user-defined and we use $G_{\max}$= 5 unless otherwise stated. When $G_{\max}$ is reached the total number of oviposition events ($N_{o}$) is calculated:


$$N_{o}=\;\sum_{G=1}^{G_{\max}}N_{\left(G\right)}^{P\text{\textbackslash{}venus}}$$ (11c)

$N_{o}$ is used below to weight the responses for each cycle ($R_{I(G)}^{S\varphi }$) to calculate the overall response ($R_{I\;}^{T}$). Cross resistance can be incorporated using correlated responses (see Equations 8c and 8d) such that each correlated response each cycle is also weighted by the number of oviposition events:


$$R_{I\;}^{T}=\;\sum_{G=1}^{G_{\max}}\left(R_{I(G)}^{S\varphi }+{{(\alpha }_{JI}R}_{J(G)}^{S\varphi })\right)\frac{N_{\left(G\right)}^{P\text{\textbackslash{}venus}}}{N_{o}}$$ (11d)

The mean PRS of the next generation is then calculated as:


$${\overset{―}{z}}_{I}^{Int^{\prime }}=\;{\overset{―}{z}}_{I}^{Int}+\;R_{I\;}^{T}$$ (11e)

In the absence of refugia and dispersal, ${\overset{―}{z}}_{I}^{Int^{\prime }}\;$becomes the mean PRS of the next generation.

#### Methods Section 3.3: Calculating the insecticide selection differential for each gonotrophic cycle (“polysmooth”).

We must account for mosquitoes encountering complex insecticide deployments which impacts the level of selection and therefore the insecticide selection differential.

This involves updating Equation 4b(♂), to allow male mosquitoes to encounter just insecticide i, just insecticide j or both insecticide i and j in their single round of selection (S5 File, Equations 12a(♂) to Equation 12h(♂). Equation 4b(♀) is similarly updated to allow female mosquitoes to encounter just insecticide i, just insecticide j or both insecticide i and j in each cycle.

The mean PRS of the females laying eggs in each cycle (${\overset{―}{z}}_{I(G)}^{P\text{\textbackslash{}venus}}$) is calculated from all insecticide encounters, where the superscript “P” indicates the parental population. The superscript “E” indicates the mosquitoes survived their encounter with the insecticide(s) and the superscript “u” indicates those females did not encounter the insecticide in the current cycle. The mean PRS for females in a cycle (${\overset{―}{z}}_{I(G)}^{P\text{\textbackslash{}venus}}$) is therefore:


$${\overset{―}{z}}_{I(G)}^{P\text{\textbackslash{}venus}}=\;\frac{\left(N_{i(G)}^{E\text{\textbackslash{}venus}}{\overset{―}{z}}_{I(G)}^{E\text{\textbackslash{}venus}}\right)+\left(N_{j(G)}^{E\text{\textbackslash{}venus}}{\overset{―}{z}}_{I(G-1)}^{P\text{\textbackslash{}venus}}\right)+\left(N_{ij(G)}^{E\text{\textbackslash{}venus}}{\overset{―}{z}}_{I(G)}^{E\text{\textbackslash{}venus}}\right)+\;\left(N_{\left(G\right)}^{u\text{\textbackslash{}venus}}{\overset{―}{z}}_{I(G-1)}^{P\text{\textbackslash{}venus}}\right)\;}{N_{(G)\;}^{P\text{\textbackslash{}venus}}}$$ (13a(♀))

$N_{\left(G\right)}^{P\text{\textbackslash{}venus}}$is the total number of females in cycle G laying eggs and is the sum of all females laying eggs in the current cycle:


$$N_{\left(G\right)}^{P\text{\textbackslash{}venus}}=\;N_{i(G)}^{E\text{\textbackslash{}venus}}+\;N_{j(G)}^{E\text{\textbackslash{}venus}}+N_{ij(G)}^{E\text{\textbackslash{}venus}}+\;N_{\left(G\right)}^{u\text{\textbackslash{}venus }}$$ (13b(♀))

$N_{\left(G\right)}^{u\text{\textbackslash{}venus}}$is the number of females not encountering any insecticides in cycle G. For the first cycle $N_{\left(G\right)}^{u\text{\textbackslash{}venus}}\;$is calculated as initial number of females ($N^{T\text{\textbackslash{}venus}}$, calculated from Equation 5f(♀)) multiplied by the probability of not encountering insecticides:


$$N_{\left(G=1\right)}^{u\text{\textbackslash{}venus}}=N^{T\text{\textbackslash{}venus}}\;(1-x)$$ (13c(i)(♀))

For subsequent cycles, $N_{\left(G\right)}^{u\text{\textbackslash{}venus}}$ is calculated:


$$N_{\left(G\right)}^{u\text{\textbackslash{}venus}}=\;N_{(G-1)\;}^{P\text{\textbackslash{}venus}}(1-x)\rho$$ (13c(ii)(♀))

$N_{(G-1}^{P\text{\textbackslash{}venus}}$is the number of female mosquitoes at the end of the previous cycle and ρ is the between cycle natural survival probability (calculated from Equation 10d). These individuals ($N_{\left(G\right)}^{u\text{\textbackslash{}venus}}$) have the same mean PRS as the individuals at the end of the previous cycle, i.e., ${\overset{―}{z}}_{I(G-1)}^{P\text{\textbackslash{}venus}}$.

The number surviving the encounter with insecticide i only is, for the first cycle:


$$N_{i(G=1)}^{E\text{\textbackslash{}venus}}=\left(c_{i}x{\overset{―}{K}}_{i}^{F}N^{T\text{\textbackslash{}venus}}\right)+\;\left(c_{ij}\Lambda_{i|ij}^{\text{\textbackslash{}venus}}x{\overset{―}{K}}_{i}^{F}N^{T\text{\textbackslash{}venus}}\right)$$ (13d(i)(♀))

For subsequent cycles:


$$N_{i(G)}^{E\text{\textbackslash{}venus}}=\left(c_{i}x{\overset{―}{K}}_{i}^{F}N_{(G-1)\;}^{P\text{\textbackslash{}venus}}\rho \right)+\;\left(c_{ij}\Lambda_{i|ij}^{\text{\textbackslash{}venus}}x{\overset{―}{K}}_{i}^{F}N_{(G-1)\;}^{P\text{\textbackslash{}venus}}\rho \right)$$ (13d(ii)(♀))

The number surviving the encounter with insecticide j only is, for the first cycle:


$$N_{j(G=1)}^{E\text{\textbackslash{}venus}}=\left(c_{j}x{\overset{―}{K}}_{j}^{F}N^{T\text{\textbackslash{}venus}}\right)+\;\left(c_{ij}\Lambda_{j|ij}^{\text{\textbackslash{}venus}}x{\overset{―}{K}}_{j}^{F}N^{T\text{\textbackslash{}venus}}\right)$$ (13e(i)(♀))

And for subsequent cycles:


$$N_{j(G)}^{E\text{\textbackslash{}venus}}=\left(c_{j}x{\overset{―}{K}}_{j}^{F}N_{(G-1)\;}^{P\text{\textbackslash{}venus}}\rho \right)+\;\left(c_{ij}\Lambda_{j|ij}^{\text{\textbackslash{}venus}}x{\overset{―}{K}}_{j}^{F}N_{(G-1)\;}^{P\text{\textbackslash{}venus}}\rho \right)$$ (13e(ii)(♀))

The number surviving the encounter with insecticide i and j is, for the first cycle:


$$N_{ij(G=1)}^{E\text{\textbackslash{}venus}}=c_{ij}\Lambda_{ij|ij}^{\text{\textbackslash{}venus}}x{\overset{―}{K}}_{i}^{F}{\overset{―}{K}}_{j}^{F}N^{T\text{\textbackslash{}venus}}$$ (13f(i)(♀))

And for subsequent cycles:


$$N_{ij(G)}^{E\text{\textbackslash{}venus}}=c_{ij}\Lambda_{ij|ij}^{\text{\textbackslash{}venus}}x{\overset{―}{K}}_{i}^{F}{\overset{―}{K}}_{j}^{F}N_{(G-1)\;}^{P\text{\textbackslash{}venus}}\rho$$ (13f(ii)(♀))

The frequency of the binned values of $z_{I}^{\text{\textbackslash{}venus}}$ after insecticide selection for the first cycle is:


$$F_{z_{I(G=1)}^{E\text{\textbackslash{}venus}}}=\;\left(F_{z_{I\;(G=0)}^{\text{\textbackslash{}venus}}}K_{i}^{F}xc_{i}\right)+\;\left({F_{z_{I\;(G=0)}^{\text{\textbackslash{}venus}}}c}_{ij}\Lambda_{i|ij}^{\text{\textbackslash{}venus}}xK_{i}^{F}\right)+\;\left(F_{z_{I\;(G=0)}^{\text{\textbackslash{}venus}}}c_{ij}\Lambda_{ij|ij}^{\text{\textbackslash{}venus}}xK_{i}^{F}{\overset{―}{K}}_{j}^{F}\right)$$ (13g(i)(♀))

For all subsequent cycles:


$$F_{z_{I(G)}^{E\text{\textbackslash{}venus}}}=\;\left(\left(F_{z_{I(G-1)}^{\text{\textbackslash{}venus}}}K_{i}^{F}xc_{i}\right)\;+\;\left({F_{z_{I(G-1)}^{\text{\textbackslash{}venus}}}c}_{ij}\Lambda_{i|ij}^{\text{\textbackslash{}venus}}xK_{i}^{F}\right)+\left(F_{z_{I(G-1)}^{\text{\textbackslash{}venus}}}c_{ij}\Lambda_{ij|ij}^{\text{\textbackslash{}venus}}xK_{i}^{F}{\overset{―}{K}}_{i}^{F}\right)\right)*\rho$$ (13g(ii)(♀))

$F_{z_{I\;(G=0)}^{\text{\textbackslash{}venus}}}$is the initial frequency of $z_{I}^{\text{\textbackslash{}venus}}$ (see Equation 4g(♀)) and $F_{z_{I(G-1)}^{\text{\textbackslash{}venus}}}$ is the frequency at the end of the previous cycle. $F_{z_{I(G)}^{E\text{\textbackslash{}venus}}}$is transferred to equation 13i(♀). The frequency at the end of the first cycle is:


$$\begin{array}{cc} F_{z_{I(G=1)}^{\text{\textbackslash{}venus}}} & =\;\left(F_{z_{I\;(G=0)}^{\text{\textbackslash{}venus}}}K_{i}^{F}xc_{i}\right)+\;\left({F_{z_{I\;(G=0)}^{\text{\textbackslash{}venus}}}\overset{―}{K}}_{j}^{F}xc_{j}\right)\\ & \;+\left({F_{z_{I\;(G=0)}^{\text{\textbackslash{}venus}}}c}_{ij}\Lambda_{ij|ij}^{\text{\textbackslash{}venus}}xK_{i}^{F}{\overset{―}{K}}_{j}^{F}\right)+\;\left({\;F_{z_{I\;(G=0)}^{\text{\textbackslash{}venus}}}c}_{ij}\Lambda_{j|ij}^{\text{\textbackslash{}venus}}x{\overset{―}{K}}_{j}^{F}\right)+\;\left({\;F_{z_{I\;(G=0)}^{\text{\textbackslash{}venus}}}c}_{ij}\Lambda_{i|ij}^{\text{\textbackslash{}venus}}xK_{i}^{F}\right)+\left(F_{z_{I\;(G=0)}^{\text{\textbackslash{}venus}}}(1-x)\right) \end{array}$$ (13h(i)(♀))

And for all subsequent cycles:


$$\begin{array}{cc} F_{z_{I(G)}^{\text{\textbackslash{}venus}}} & =(\;\left(F_{z_{I(G-1)}^{\text{\textbackslash{}venus}}}K_{i}^{F}xc_{i}\right)+\;\left(F_{z_{I(G-1)}^{\text{\textbackslash{}venus}}}{\overset{―}{K}}_{j}^{F}xc_{j}\right)+\left({F_{z_{I(G-1)}^{\text{\textbackslash{}venus}}}c}_{ij}\Lambda_{ij|ij}^{\text{\textbackslash{}venus}}xK_{i}^{F}{\overset{―}{K}}_{i}^{F}\right)\\ & \;+\;\left({F_{z_{I(G-1)}^{\text{\textbackslash{}venus}}}c}_{ij}\Lambda_{j|ij}^{\text{\textbackslash{}venus}}x{\overset{―}{K}}_{j}^{F}\right)+\;\left({F_{z_{I(G-1)}^{\text{\textbackslash{}venus}}}c}_{ij}\Lambda_{i|ij}^{\text{\textbackslash{}venus}}xK_{i}^{F}\right)+\left(F_{z_{I(G-1)}^{\text{\textbackslash{}venus}}}(1-x)\right))*\rho \end{array}$$ (13h(ii)(♀))

The mean PRS value of the exposed survivors (${\overset{―}{z}}_{I(G)}^{E\text{\textbackslash{}venus}}$) for each is calculated:


$${\overset{―}{z}}_{I(G)}^{E\text{\textbackslash{}venus}}=\;\left(\sum_{z_{I}^{\text{\textbackslash{}venus}}=-\infty }^{\infty }F_{z_{I(G)}^{E\text{\textbackslash{}venus}}}z_{I}^{\text{\textbackslash{}venus}}\right)/\left(N_{i(G)}^{E\text{\textbackslash{}venus}}+N_{ij(G)}^{E\text{\textbackslash{}venus}}\right)$$ (13i(♀))

The value of ${\overset{―}{z}}_{I(G)}^{E\text{\textbackslash{}venus}}$ is then returned to Equation 13a(♀) to calculate ${\overset{―}{z}}_{I(G)}^{P\text{\textbackslash{}venus}}$, which is used to calculate the selection differentials (Equation 11a(i), Equation 11a(ii)) and response (Equation 11b).

Implementing the multiple cycle model allowing for dispersal between the intervention site and refugia in each cycle is given in the S6 File.

### Methods Section 4: Model Calibration, and its Application to Evaluating IRM Strategies

#### Methods Section 4.1. Parameter Estimation/Model Calibration.

Details of parameter estimation and model calibration are found in the S3, S2 and S4 Files. The S3 File details the estimation of the standard deviation ($\sigma_{I}$) and the estimation of how $\sigma_{I}$ changes with ${\overset{―}{z}}_{I}$. The S2 File details model calibration assuming a “novel” insecticide would be expected to have an average of 10 years continuous deployment until 10% bioassay is reached; this was previously used in the “polyres” model [11] using a fixed $\sigma_{I}$. The S4 File details the estimation of fitness cost selection differentials.

#### Methods Section 4.2. Simulation Capability of the Models and Importance of Parameters.

The first simulation showcase (in S7 File) demonstrates the breadth of IRM strategies that “polytruncate” and “polysmooth” can simulate. This considers both the deployment strategy and the switching strategy for monotherapies (Fig A in S7 File), mixtures (Fig B and C in S7 File), micro-mosaics (Fig D in S7 File) and combinations (Fig E in S7 File), and how these simulations can be extended to include more than two insecticides.

**Fig 9:**
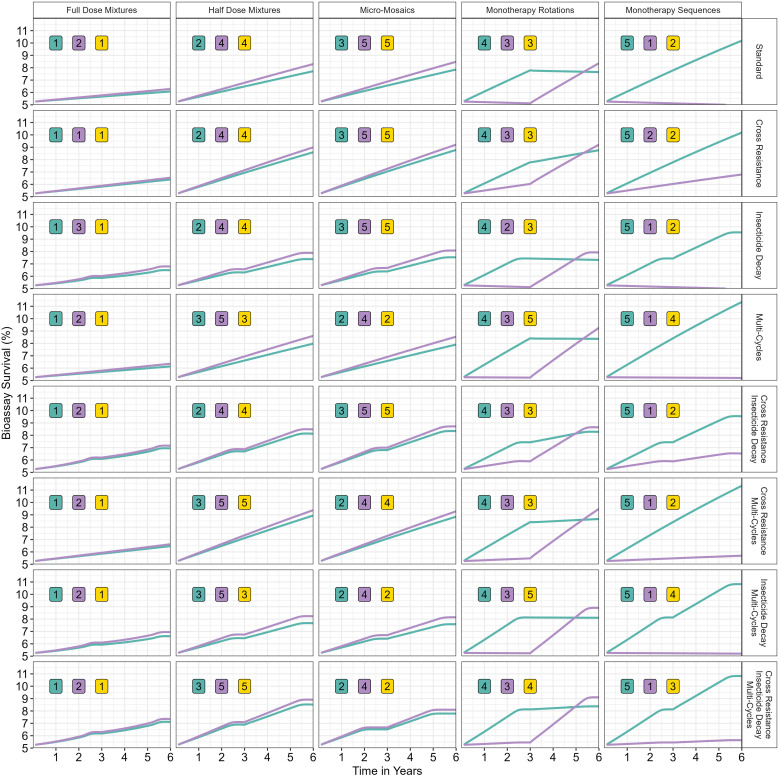
Demonstration of the “polysmooth” model: assessing IRM strategies in the presence/absence of cross resistance, insecticide and multiple gonotrophic cycles. Plots are stratified by insecticide resistance management strategy (columns), and model features (rows). The green line is insecticide i and the purple line insecticide j, with their ranking (shown in the numbered boxes: 1 is best and 5 is worst) based on mean bioassay survival across the five IRMs in each row. The yellow boxes contain rankings based on the mean of both insecticides.

The second simulation showcase (Fig 9) demonstrates how the inclusion of insecticide decay, cross resistance and multiple cycles impacts the comparative performance of IRM strategies. This is showcased by running simulations including/excluding each of these features. This was conducted using an example set of simulations using the IRM strategies of: monotherapy sequences, monotherapy rotations, micro-mosaics, full-dose mixtures and half-dose mixtures. Simulations were run for 6 years (two 30-generation deployment intervals), to demonstrate differences in the comparative performance of the IRM strategies

These example simulations use the parameters: $h_{I}^{2}$ = 0.2; $h_{J}^{2}$=0.25; x = 0.7; m = 0.7; $S_{I}^{\varphi \text{\textbackslash{}venus}}$= 0.2; $S_{I}^{\varphi \text{\textbackslash{}mars}}$ = 0.2; C = 0.7; θ= 0.3. The starting mean PRS was 50. The following parameters were used when required: cross resistance ${(\alpha }_{JI}$ = 0.3), multiple cycles ($G_{\max}$= 5), insecticide decay (${\delta_{b}}^{i}$= 0.015, ${\delta_{r}}^{i}$= 0.08, $\tau_{b}^{i}$= 20). The insecticides had the same decay profiles.

#### Methods Section 4.3. Simulations: Comparing polytruncate versus polysmooth.

**Fig 10:**
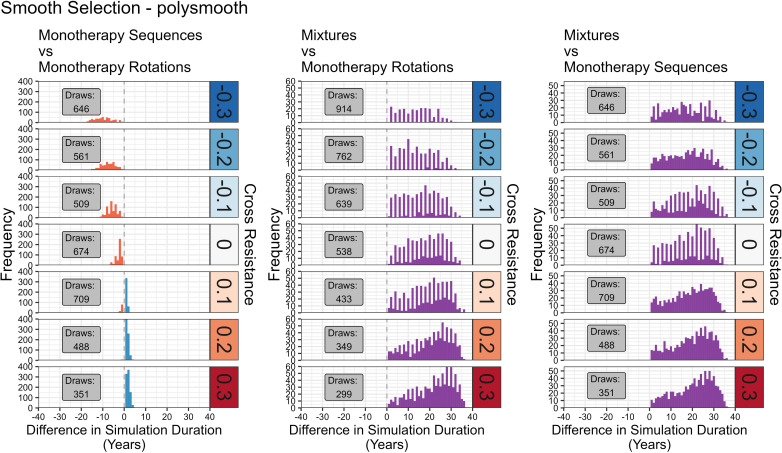
Comparison of Sequences, Rotations and Mixtures for Polysmooth. Left column: Sequences versus Rotations; Centre column: Mixtures vs Rotations; Right column: Mixtures vs Sequences. Colours indicate which strategy had the longer operational lifespan in the direct comparison. Red = rotations longer. Blue = Sequences longer. Purple = Full-Dose Mixtures longer. Draws were excluded from the histogram, but reported in the grey boxes. The number of draws is that obtained from the 5000 parameter sets summarised in the panel. The rows indicate the amount of cross resistance between the two insecticides. Simulations were terminated when no insecticides were available for deployment (because resistance had evolved to them all), or the 500-generation (~50 years) cap was reached. Simulation duration (in years) therefore measures operational lifespan of the IRM strategy. Alternating high and low bars are because simulations with two insecticides are more likely to terminate in even years, thus a difference as a multiple of two years is more likely than as a multiple of 1 year.

**Fig 11:**
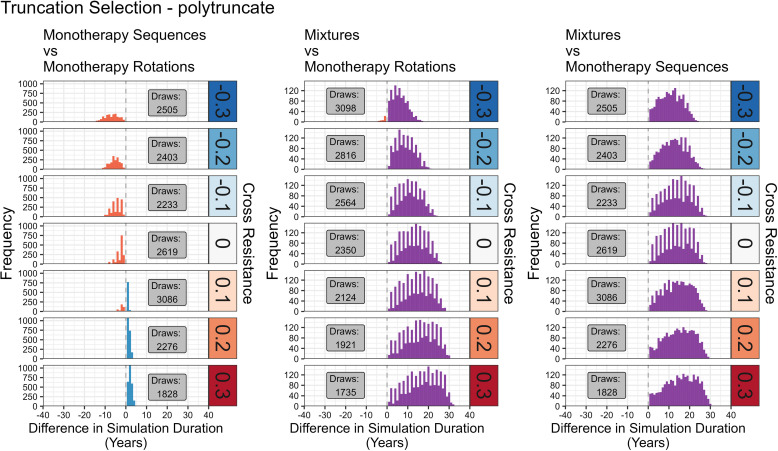
Comparison of Sequences, Rotations and Mixtures for Polytruncate. As Fig 10, but with results obtained from “polytruncate”.

**Fig 12:**
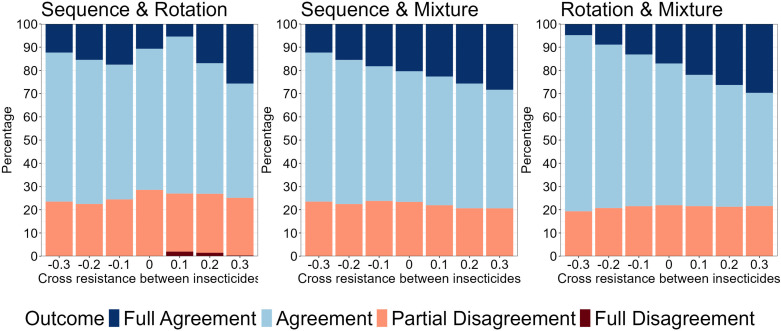
Comparing Outcomes between Polysmooth and Polytruncate. The data shows the extent of agreement for the strategy outcome between the “polysmooth” and “polytruncate” models for the same parameter inputs. Colours indicate how the strategies performed in both models. Dark blue = both models indicate the same strategy performed best (“full agreement”). Light blue = both strategies draw in both models (“agreement”). Light red = both strategies drew in one model, but one strategy performed better than the other in the other model (“partial disagreement”). Dark red = Models diverge as to which strategy performed best (“full disagreement”).

We compare results obtained from “polytruncate” and “polysmooth” against each other (Figs 10–12) and against results in the IRM modelling literature. To allow comparisons with the previous literature, simulations consisted of 2 insecticides (with equivalent properties) deployed as monotherapy sequences, monotherapy rotations or full-dose mixtures. The starting resistance was ${\overset{―}{z}}_{I}$=0. Simulations had a 10-generation deployment interval (i.e., the opportunity to change deployment occurred every 10 generations). Insecticide decay was not included, thereby allowing comparison to the previous “polyres” model which did not include insecticide decay [11]. Simulations used 10% and 8% as bioassay survival withdrawal and return thresholds respectively [11].

The parameter space of coverage (0.1-0.9), dispersal (0.1-0.9), fitness costs (0.04-0.58), female exposure (0.4-0.9), male exposure (0–1), heritability (0.05-0.3) was sampled using a Latin hypercube [34] to generate 5000 parameter sets. These were replicated across seven cross-resistance values (-0.3 to 0.3 at 0.1 intervals) for a total of 35,000 parameter sets. The same parameter sets were used for each strategy and model (“polytruncate” and “polysmooth”) to allow for direct comparisons. Simulations were terminated when no insecticides were available for deployment (because resistance had evolved to them all), or the 500-generation (~50 years) cap was reached. Simulation duration (in years) therefore measures operational lifespan of the IRM strategy and is used in the comparisons i.e., between sequences-rotations, sequences-mixtures, and rotations-mixtures. Strategies could therefore “win”, “lose” or “draw” in each comparison. We use the simulation duration metric for consistency and comparison with previous studies [11,20,21,35].

Agreement between “polytruncate” and “polysmooth” was assessed:

1. Full Agreement: both models indicate the same strategy performed best.
2. Agreement: both strategies draw in both models. Note that draws may not be truly equivalent because one may become superior if the simulations were allowed to run longer than the user-defined 500 generations).
3. Partial Disagreement: Both strategies drew in one model, but one strategy performed better than the other in the other model.
4. Full Disagreement: Models diverge as to which strategy performed best.

#### Methods Section 4.4. Importance of Multiple Gonotrophic Cycles for Micro-Mosaics.

The importance of including multiple cycles for evaluating more complex IRM strategies was evaluated by comparing two insecticides (i and j) deployed in rotation versus being deployed as a micro-mosaic (where $c_{i}$ = 0.5 and $c_{j}$ = 0.5). Each strategy used the same 5000 randomly sampled parameter values, allowing for direct comparison between the strategies. Parameters were sampled from uniform distributions using Latin hyperspace sampling [34]: Heritability ($h_{I}^{2}$)[0.05 to 0.3]; Female insecticide exposure (x) [0.4 to 0.9]; Male insecticide exposure (m)[0–1]; Female Fitness Cost ($S_{I}^{\varphi \text{\textbackslash{}venus}}$) [0.04 to 0.58]; Male Fitness Cost ($S_{I}^{\varphi \text{\textbackslash{}mars}}$) [0.04 to 0.58]; Intervention (C) [0.1 to 0.9]; Dispersal (θ)[0.1 to 0.9]. The simulations were run using a single cycle, five cycles with no natural mortality, and finally with five cycles with daily natural survival (d=0.8) and a cycle length of three days. Simulations were run for 500 generations (50 years), and the difference in bioassay survival to insecticide i was reported each year. No withdrawal threshold value was used so all simulations ran for the 50-year time horizon.

#### Methods Section 4.5. Estimating Mosquito Age Profiles given Resistance and Interventions.

The multiple cycle methodology allows the estimation of mosquito age profiles. Mosquito age is a key metric for disease transmission. The mosquito must become infected and then survive the extrinsic incubation period before becoming infectious. For *Plasmodium falciparum* this is approximately 10 days (corresponding to ~3 cycles). $N_{(G}^{P\text{\textbackslash{}venus}}$, calculated in Equation 13b(♀), is the number of female mosquitoes surviving in each cycle, and is the age distribution. The IRM performance of interventions is a secondary consideration, with disease control being the primary consideration. As “polysmooth” is not an explicit population dynamics model, the exact number of mosquitoes emerging on any day is unknown, however we assume, in the absence of interventions 100,000 female mosquitoes would successfully complete the first cycle. We run snap-shot simulations (i.e., single-generation) covering 10 cycles to generate the age distributions and make some simplifying assumptions to aid their interpretations. First, coverage = 1 (no refugia is present) and x = 0.7. This means that mosquitoes have a 70% probability of encountering the insecticidal intervention in each cycle. These snap-shots do not consider change in resistance, male exposure, heritability and fitness costs so their associated parameters do not need specifying. Insecticide efficacy and the mean PRS is specified for each snap-shot. A natural survival rate between cycles (ρ) of 0.729 was used. For all snap-shots the age profile of the population in the absence of insecticide deployment is also reported. We run these snap-shot simulations considering the following three deployment strategies: monotherapies, mixtures and micro-mosaics.

The impact of both increasing resistance and decaying efficacy for monotherapy deployments was investigated. Insecticide efficacy is included from 0.1 to 1 at 0.1 intervals. As the 10% bioassay is the designated withdrawal threshold for monotherapy formulations [22] we perform this with a cohort of mosquitoes with 0, 5 and 10% bioassay survival.

For insecticide mixtures the considerations are both efficacy and resistance to either insecticide. Further when considering next-generation ITNs substantial resistance to the pyrethroid partner may already be present. The insecticides may decay at different rates. These snap-shots were run with insecticide resistance (0, 10, 75% bioassay for either insecticide) and insecticide efficacy (0.25, 0.5, 0.75, 1 for either insecticide) and all permutations for both insecticides, giving a total of 144 snap-shots.

Finally we consider the performance of micro-mosaics versus full-dose and half-dose mixtures to understand how the inclusion of the micro-mosaic’s “temporal mixture” effect influences mosquito age profiles. For the micro-mosaic simulations: coverages are $c_{i}$ = 0.5 and $c_{j}$ = 0.5. For each insecticide included in the snap-shot simulations the level of resistance (measured as bioassay survival) is one of 0, 10, 50, 75%. This evaluates whether age distributions caused by micro-mosaic deployment are similar to those obtained using a full-dose mixture or a half-dose mixture. Changes in age distribution are important from a disease control perspective and are an important additional consideration for the evaluation of micro-mosaics.

#### Methods Section 4.6. Implications of the Standard Deviation of the PRS on Time to Resistance.

We explore the implications of the standard deviation of the mean PRS on the time taken for the target population to reach 10% bioassay survival for single insecticide simulations. Simulations were capped at a maximum of 500 generations. Simulations were run by varying the value of the standard deviation $\sigma_{I}$, which remained fixed for the duration of a single simulation. The input values of $\sigma_{I}$ were varied from 5 to 100, at 5 unit increments, for a total of 20 $\sigma_{I}$ values. For each simulation the time to 10% bioassay survival was recorded. This was replicated for 30 randomly sampled parameter sets from female exposure (0.4 to 0.9), male exposure (0–1) and heritability (0.05 to 0.3). Intervention coverage was set to 1 for all simulations, fitness costs were not included and insecticide decay was not included to allow for ease of interpretation. This was repeated for both the polytruncate and polysmooth model branches.

## 3. Results

### 3.1. Show case of model simulation capabilities

A demonstration of the IRM simulation capabilities of the “polytruncate” and “polysmooth” models are provided in the S7 File. We show example simulations for monotherapies and mixtures (which both “polytruncate” and “polysmooth” can perform). We further demonstrate the ability of “polysmooth” to simulate the micro-mosaic and combination IRM strategies. These also demonstrate that simulations can be run for IRM strategies using more than two insecticides.

The second part of the model show case demonstrates how the inclusion of various features of the model can change the performance ranking of IRM strategies. Fig 9 shows that regardless of the features used (and their permutations), the full-dose mixture consistently performed as the best IRM strategy. The performance of the other strategies was far more sensitive to the inclusion of the other features. For example including multiple cycles, insecticide decay and cross resistance resulted in micro-mosaics performing second best (behind full-dose mixtures). Including just insecticide decay and cross resistance (i.e., omitting multiple cycles) resulted in micro-mosaics becoming the worst performing strategy (Fig 9).

### 3.2. Comparing the “polysmooth” and “polytruncate” Models

“Polysmooth” (Fig 10) and “polytruncate” (Fig 11) models give similar results to one another and are qualitatively similar to results obtained previously using the “polyres” model [11], i.e., full-dose mixtures are best, often extending the simulation duration of the insecticides by over 10 years. The benefit of full-dose mixtures versus rotations or sequences increased with increasing cross resistance. The benefit of full-dose mixtures decreased with negative cross resistance, which is expected as negative cross resistance maintains lower resistance levels for all strategies. Rotations perform better than sequences if there is negative cross resistance. Sequences perform better than rotations if there is positive cross resistance. The benefit of choosing either rotations or sequences over each other were small, rarely more than five years duration differences. These general conclusions are in-line with monogenic models [21,30,35].

Fig 12 shows the percentage agreement between the models and shows “full disagreement” was rare, occurring only when comparing sequences and rotations. The choice between sequences and rotations has previously been shown to be highly unpredictable [30], and therefore divergence between “polysmooth” and “polytruncate” is may be expected for these two IRM strategies. Partial agreements were most common, due to the frequency of draws. This high agreement between the models gives confidence in the model predictions as they are robust to the assumptions made when calculating insecticide selection.

### 3.3. Demonstrating the importance of multiple gonotrophic cycles and natural survival: micro-mosaics vs rotations

**Fig 13:**
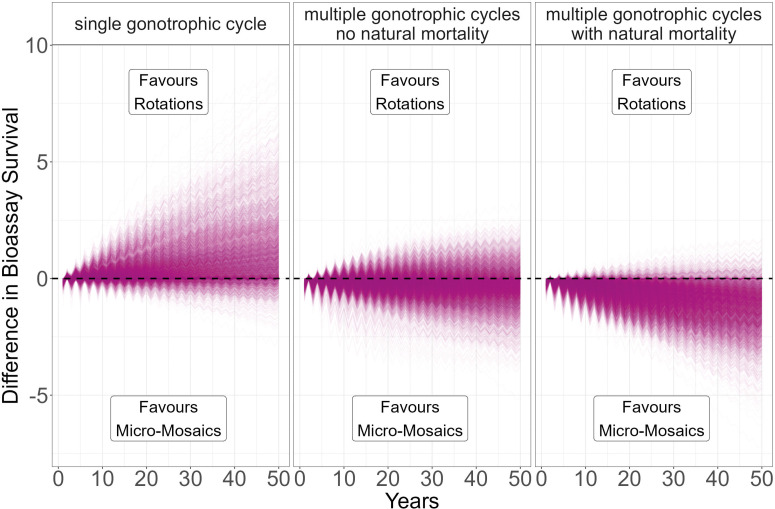
The impact of including multiple gonotrophic cycles and natural survival on the performance of deploying insecticides i **and** j **in rotation versus micro-mosaics.** Values above zero indicate the rotation strategy performed best, and values below zero indicates the micro-mosaic strategy performed best. **Left Plot:** The model is run only for a single cycle. **Centre Plot:** The model is run allow for multiple gonotrophic cycles (5 cycles) but natural survival is not included. **Right plot:** The model is run with multiple cycles and allowing for natural mortality (gonotrophic length = 3 days, natural daily survival = 0.8).

While the quantitative effect may not be dramatic, the inclusion of multiple cycles and natural mortality altered the qualitative results: micro-mosaics go from performing worse than rotations (single cycle, Fig 13 left panel) to performing equally well (multiple cycles, Fig 13 centre panel), to performing better than micro-mosaics (multiple cycles with natural mortality, Fig 13 right panel). The reason for this is that the benefit of halving the selection to each insecticide over space becomes more pronounced as the first cycle becomes most important for insecticide selection combined with its “temporal mixture” effect being more beneficial when including natural mortality. This highlights why the inclusion of multiple cycles and natural mortality was required for the model (we stress it is also much more biologically realistic).

### 3.4. Estimating mosquito age profiles given insecticide deployments and insecticide resistance

**Fig 14:**
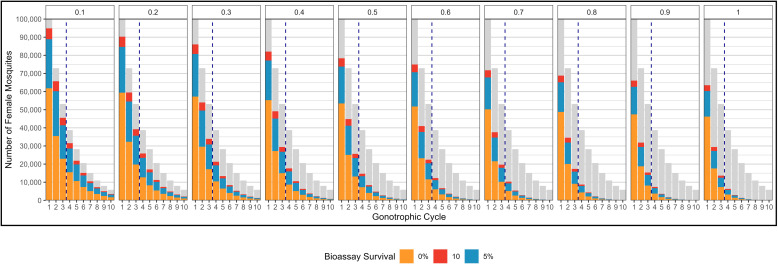
How mosquito age distributions change given insecticide efficacy and resistance. The grey bars indicate the age profile if no intervention had been deployed. The colored bars represent age distribution at different levels of IR quantified as bioassay survival. The value at the top of each panel is insecticide efficacy.

**Fig 15:**
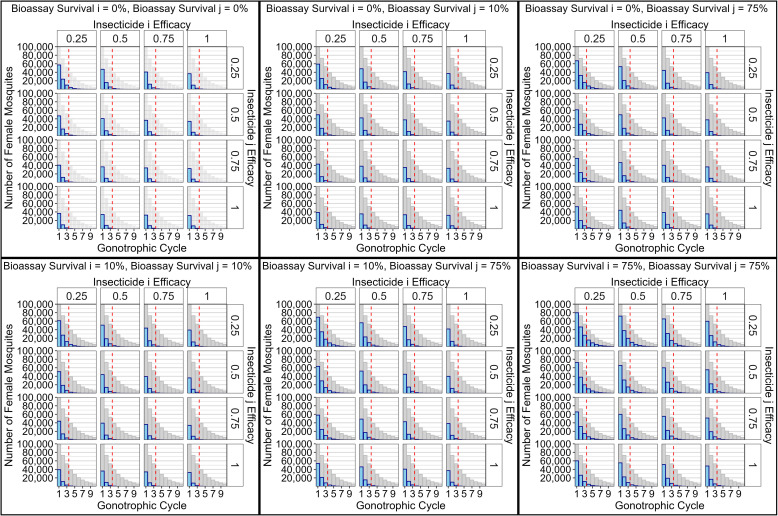
Changes in mosquito age distributions given resistance and insecticide efficacy for mixtures. The grey shaded area is the age profile if the intervention was not deployed. The blue bars are the age profile given the values of insecticide efficacies and resistance specified at the top of each plot. A red vertical dashed line is plotted at 10 days of age which is the minimum age mosquitoes can become infectious.

**Fig 16:**
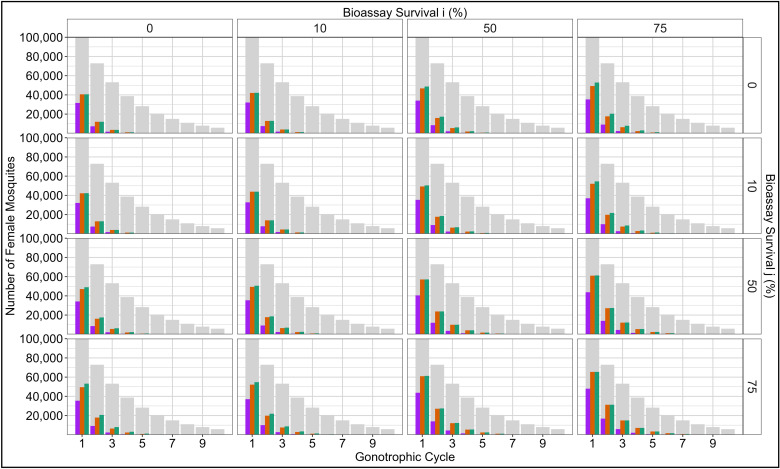
Age profile comparisons depending on whether IRM is deployed as micro-mosaics, half-dose mixtures and full-dose mixtures. The colored bars are the age distributions that occur if there is no intervention (grey), if full-dose mixtures are deployed (purple), if micro-mosaics are deployed (green) or if half-dose mixtures (HD50%_HD50%) are deployed (orange).

Fig 14 shows the age distributions when considering the deployment of a single insecticide, and how the ability of the insecticide to control mosquitoes may be compromised by both insecticide resistance and declining insecticide efficacy. As the level of resistance increases, so does the number of female mosquitoes surviving multiple cycles, and this survival is further increased when insecticide efficacy declines. Increasing numbers of female mosquitoes surviving beyond the extrinsic incubation of the pathogen correspond to expected increases in transmission. Fig 15 shows the age distributions when considering insecticide mixtures, and how as insecticides decay and resistance levels increase to the constituent partners the mixtures become less effective in suppressing the age profile. Fig 16 shows the comparison between micro-mosaics, full-dose mixtures and half-dose mixtures. The full-dose mixtures are consistently the best at suppressing the age distribution, and more importantly it shows that micro-mosaics and half-dose mixtures have near identical effects. This suggests that micro-mosaics may be a viable alternative to half-dose mixtures and therefore require further evaluation based on their capability to manage insecticide resistance.

### 3.5. Implication of the standard deviation of the PRS on time to resistance

**Fig 17:**
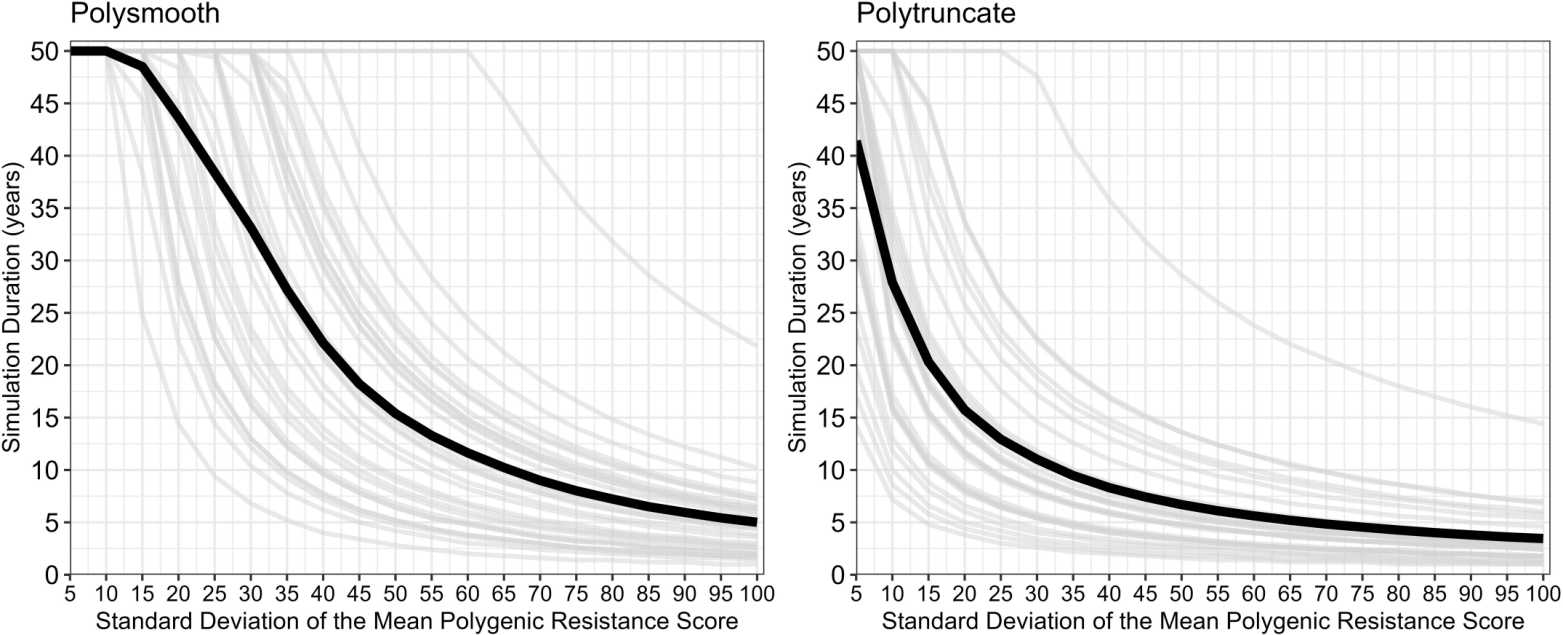
Impact of the standard deviation of the PRS on time to resistance. Each grey line represents a single parameter set, with a total 30 parameter sets generated from sampling uniform distributions of heritability, female insecticide exposure and male insecticide exposure), with the time taken to reach 10% bioassay survival. The black line is the mean value for these simulations.

Fig 17 demonstrates the impact of the standard deviation of the mean PRS and shows that as the standard deviation increases, the time taken to reach 10% bioassay survival decreases. This finding applies to both the polysmooth or polytruncate models.

## 4. Discussion

We have presented a methodology for dynamically modelling the evolution of polygenic IR. This model provides a more sophisticated method for calculating the evolutionary trajectory of IR, resulting in its ability to evaluate the impacts of a larger number of parameters compared to previous models assuming polygenic resistance [11–14]. Many of these factors (e.g., insecticide decay, insecticide dosing and cross resistance) are suspected to be highly important in determining the optimal IRM strategy. Feedback from our colleagues involved in policy decisions indicates their inclusion is required to make the modelling more compelling and able to influence insecticide deployment policy and product development.

### 4.1. Benefits of the dynamic model

The benefit of the developed dynamic polygenic model comes from an increase in the biological and operational realism, notably the simultaneous inclusion of three important factors: insecticide dosing, insecticide decay and cross-resistance. These three factors are considered critical for evaluating the relative performance of IRM strategies despite being frequently absent from models [9,15].

An additional advantage of these dynamic models is the comparative ease with which an insecticide armoury of more than 2 insecticides can be investigated. Monogenic models are generally limited to two insecticides as tracking more than 2 loci involves tracking large numbers of genotypes and linkage dis-equilibriums, where the calculations rapidly become computationally unfeasible. Allowing larger insecticide armouries allows for the evaluation of more complex IRM strategies, for example the combination strategy with rotations of IRS insecticides (S7 File).

We also demonstrated the importance of extending the model to incorporate multiple cycles, and how this capability is required for evaluating micro-mosaics and combinations (Fig 13). The performance of micro-mosaics against rotations and mixtures (both full and reduced dose) is still to be evaluated across a wide biological and operational parameter space and is the subject of a separate piece of work [36]. Furthermore, the multi-cycle model can be used to estimate the age profile of mosquito populations (Figs 14–16), which will be a key metric for linking IRM and disease transmission.

An additional finding from the model was that increasing the standard deviation of the PRS of the population decreases the time for operationally relevant resistance to occur (Fig 17 and Figs A and B in S2 File). Often, the point estimate from susceptibility bioassays are reported, however we would argue that the variability of these estimates contains pertinent information regarding the evolutionary potential of those populations. The main susceptibility bioassay database (Malaria Threat Map) only includes the point estimate of bioassays and not the variability of these estimates.

### 4.2. Consistency of dynamic models with previous model of IR

Despite these differences in complexity, the dynamic models generated results consistent with a previous quantitative genetics model [11] and monogenic models [21,30,35]. Most notably full-dose mixtures are more effective than deploying monotherapies as sequences or rotations, and there is little difference between the sequence and rotation strategies (Figs 11 and 12) [11]. This demonstration of consistency is extremely important as it implies that the evaluation of IRM strategies is not critically dependent on the underlying assumptions made in the different methodologies, in particular whether IR is assumed to be a monogenic or polygenic trait. As noted above, this greatly increases the confidence with which we can make policy recommendations.

The need to ensure models are robust to their underlying assumptions was a primary driver of implementing insecticide mortality as both a truncating and probabilistic process. The probabilistic selection process is likely to better account for the variation in insecticide contact times, and its ability to model multiple-cycles certainly makes it the more versatile branch. Nevertheless the truncation methodology may be more suitable when considering interventions where exposure is more uniform such as larviciding or fogging, or indeed in agriculture. Truncation selection is the more classical way of considering insecticide selection [37].

### 4.3. Future applications for the dynamic models

Several publications have arisen from meetings in which modellers and policy makers met to identify key knowledge gaps that need to be addressed. For example, the malERA consortium identified the need to evaluate mosaics and combinations of pyrethroid-ITNs with non-pyrethroid IRS [38] and the Rex Consortium noted half-dose mixtures required evaluation [39]. The models presented here have the capacity to fill these knowledge gaps, and we therefore highlight four priority areas to explore in scenario-specific projects:

1. Insecticide decay has recently been demonstrated as an important, yet often absent, parameter in models evaluating IRM strategies when considering monogenic resistance [15]. This issue requires further evaluation when considering polygenic resistance[40].
2. It is suspected that the benefit of mixtures is maximal when resistance to both insecticides is at low levels [16]. Current next-generation mixture ITNs include a pyrethroid as one of the insecticide partners, to which there is already widespread resistance. Next-generation mixture ITNs have demonstrably better epidemiological efficacy than standard (pyrethroid-only) ITNs [41,42]. Evaluating the long-term evolutionary consequences of mixture ITNs where there are both high levels of resistance to one partner (e.g., the pyrethroid) and reduced insecticide dosages is an issue of immediate operational relevance.
3. Micro-mosaics are likely to be a logistically challenging strategy to implement. This requires their thorough evaluation in computational simulations before extensive time, resources and finances are used in field trials. Simulations can evaluate how robust this strategy is to unexpected issues which may occur in its deployment, for example supply-chain issues due to differential distribution pathways [43], and therefore potential mis-matched coverages of insecticides in the same setting.
4. The evaluation of combinations for IRM is still in its infancy [38], despite inclusion as a recommended IRM strategy in the Global Plan for Insecticide Resistance Management [4]. This strategy was recently evaluated [19], however this methodology did not consider a major putative benefit, i.e., the ability to rotate out the insecticide used for the IRS independently of the pyrethroid-ITN. The modelling presented above can achieve this [44].

## Conclusion

The dynamic models developed above allow simulation of many IRM strategies which would be impractical to evaluate in field settings helping to identify whether they are worth pursuing and, importantly, whether the proposed benefits of such strategies are sufficiently large to justify the required procurement and deployment systems. The development of the models described above, and their demonstrated near-consistency with previous work, therefore plays an important role in guiding insecticide deployment programmes aimed at controlling mosquitoes that spread several important human diseases. In particular, insecticide-based control programmes have been instrumental in substantially reducing the malaria disease burden over the last 25 years. These gains are now threatened by increasing levels of IR which is likely to require ever more complex deployment policies to counter-act.

## Supporting information

S1 File Symbols used in the “polysmooth” and “polytruncate” Models. (DOCX)

S2 FileCalibration of the Exposure Scaling Factor (β) for “novel” insecticides.(DOCX)

S3 File Fixed and Variable Standard Deviations. (DOCX)

S4 File Fitness cost Selection Differential Values. (DOCX)

S5 File Calculating the Male Insecticide Selection Differential with Complex Insecticide Encounters. (DOCX)

S6 File Multiple gonotrophic cycles and dispersal. (DOCX)

S7 File Examples of insecticide resistance management strategies. (DOCX)
