# Supplementary material for: Simulating dynamic insecticide selection pressures for resistance management in mosquitoes assuming polygenic resistance
Source: PLoS Comput Biol. 2025 Apr 28;21(4):e1012944. doi: 10.1371/journal.pcbi.1012944 (PMC12058183; doi:10.1371/journal.pcbi.1012944)
Supplement: S1 File — (DOCX) [file pcbi.1012944.s001.docx]

**S1 File: Symbols used in the “polysmooth” and “polytruncate” Models**

In this supplement we provide detailed descriptions of all symbols used in the mathematical model (Table A to E in S1 File). Fig A in S1 File provides additional detail on what is meant by $F_{z_{I}}.$


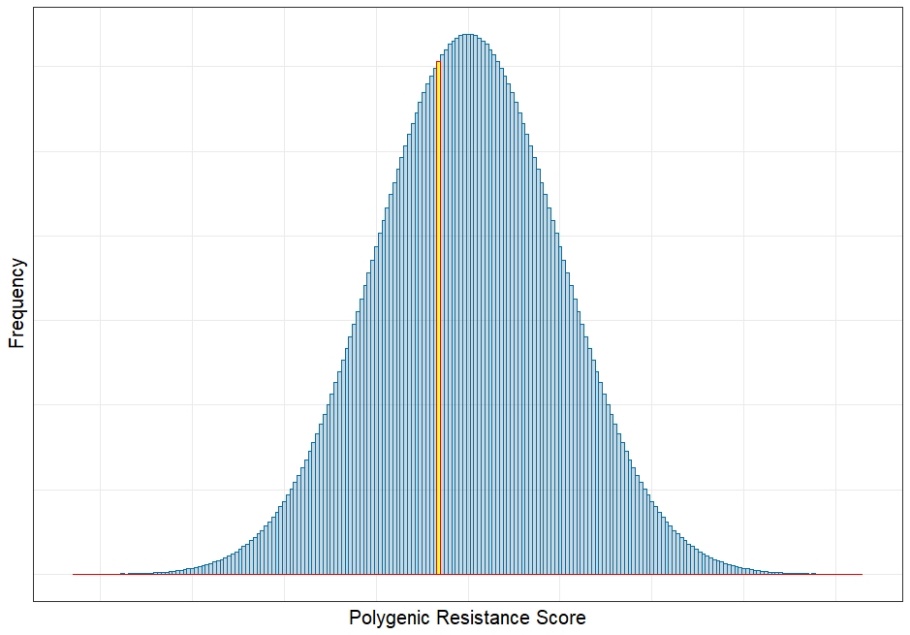


**Fig A:** **Graphical explanation of** $\boldsymbol{F}_{\boldsymbol{z}_{\boldsymbol{I}}}$**.** The model code tracks four vectors, two containing the binned values of the polygenic resistance score ($z_{I}^{\text{♀}}$ or $z_{I}^{\text{♂}}$) in the Normal distribution (illustrated in this plot), and two vectors containing the corresponding frequencies of the binned values of $z_{I}^{\text{♀}}$ or $z_{I}^{\text{♂}}$. The values of $z_{I}^{\text{♀}}$ or $z_{I}^{\text{♂}}$are grouped into a number of small bins (which defines the length of the vector). Numerical precision is achieved providing the number of bins is sufficiently high.

| **Table A: Symbols introduced in Methods Section 1.1 to 1.4** | | |
| --- | --- | --- |
| Symbol | Description | Value/Ranges |
| $K_{i}^{B}$ | The bioassay survival of a mosquito calculated from its polygenic resistance score (PRS). | Internally calculated. |
| $K_{max}$ | The maximum bioassay survival. | 1 |
| $z_{I}$ | The PRS of a mosquito for trait $I$ which gives resistance to insecticide $i$. | Calculated Internally |
| $z_{50}$ | The polygenic resistance score which gives 50% bioassay survival. | 900 (see Hobbs et al, 2023) |
| $n$ | The slope of the Michaelis-Menten Equation | 1 |
| $K_{i}^{F}$ | The field survival of a mosquito given their bioassay survival (dependent on the PRS). | Internally calculated |
| $\varphi_{1}$ | The regression coefficient between the field (experimental hut) survival and bioassay survival. | 0.48 [1] |
| $\varphi_{2}$ | The regression intercept between the field (experimental hut) survival and bioassay survival. | 0.15 [1] |
| $\zeta_{\tau}$ | The current concentration of the insecticide. | Internally calculated |
| $\zeta_{0}$ | The deployed concentration of the insecticide. | Insecticide dependent |
| $k$ | The instantaneous decay rate of the insecticide. | Insecticide dependent |
| $\tau$ | The number of mosquito generations since the insecticide was deployed. | Internally calculated |
| $\omega_{\tau}^{i}$ | The current efficacy of the insecticide after $\tau$ generations against fully susceptible mosquitoes ($z_{I}$ < 0) | Internally Calculated |
| $\omega_{0}^{i}$ | The initial deployed efficacy of the insecticide against fully susceptible mosquitoes ($z_{I}$ < 0). | full dose = 1, half dose = 0.5,  User input. |
| ${\delta_{b}}^{i}$ | The base decay rate of the insecticide, where the insecticide decays slowly. | Insecticide dependent, user input |
| $\tau_{b}^{i}$ | The “threshold” number of generation post-deployment at which there is a change in the decay rate. | Insecticide dependent, user input |
| ${\delta_{r}}^{i}$ | The rapid decay rate occurring after the threshold generation ($\tau_{b}^{i}$) is exceeded. | Insecticide dependent, user input |
| $\varphi_{3}$ | The regression coefficient between the mean PRS from bioassays and the standard deviation from those bioassays. | 0.4 modelled estimate (see Supplement 3) |
| $\varphi_{4}$ | The regression intercept between the mean PRS from bioassays and the standard deviation from those bioassays. | 18 modelled estimate (see Supplement 3) |
| $\sigma_{I}$ | The standard deviation of trait $I$. | “polytruncate”=20, “polysmooth”=50, or internally calculated |
| $\sigma_{I}^{Int}$ | The standard deviation of trait $I$ in the intervention site. | “polytruncate”=20, “polysmooth”=50, or internally calculated |
| $\sigma_{I}^{Ref}$ | The standard deviation of trait $I$ in the refugia. | “polytruncate”=20, “polysmooth”=50, or internally calculated |

| **Table B: Symbols introduced in Methods Section 2.1 to 2.7** | | |
| --- | --- | --- |
| Symbol | Description | Value/Range |
| $R_{I}^{S\phi}$ | The response to selection due to both insecticide selection and fitness costs. | Internally Calculated |
| $R_{I}^{\phi}$ | The response to selection due to fitness costs only. | Internally Calculated |
| $h_{I}^{2}$ | The narrow sense heritability of the trait, i.e., the proportion of the selection differential is inherited by the next generation. | Uniform(0.05 – 0.3) |
| $S_{I}^{S\phi}$ | The overall selection differential resulting from insecticide selection and fitness costs. | Internally Calculated |
| $\beta$ | A factor which is used to calibrate the model to the desired timescale. | “polytruncate”=1, “polysmooth”=10 |
| $S_{I}^{S}$ | The selection differential due to insecticide selection. | Internally Calculated |
| $S_{i}^{\phi}$ | The selection differential due to fitness costs. | Internally Calculated |
| $S_{I}^{S\phi\text{♀}}$ | The female selection differential due to insecticide selection and fitness costs. | Internally Calculated |
| $S_{I}^{S\text{♀}}$ | The female selection differential due to insecticide selection. | Internally Calculated |
| $S_{I}^{\phi\text{♀}}$ | The female selection differential due to fitness costs. | Internally Calculated |
| $S_{I}^{S\phi\text{♂}}$ | The male selection differential due to insecticide selection and fitness costs. | Internally Calculated |
| $S_{I}^{S\text{♂}}$ | The male selection differential due to insecticide selection. | Internally Calculated |
| $S_{I}^{\phi\text{♂}}$ | The male selection differential due to fitness costs. | Internally Calculated |
| $\bar{z}_{I}$ | The mean PRS of trait $I$ giving resistance to insecticide $i.$ | Internally Calculated |
| $F_{z_{I}}$ | Relative frequency of $z_{I}$ in the population. | Internally Calculated |
| $\bar{z}_{I}^{\text{♀}}$ | The mean PRS of female mosquitoes prior to any selection. | Internally Calculated |
| $\bar{z}_{I}^{P\text{♀}}$ | The mean PRS for parents (mothers) after insecticide selection. | Internally Calculated |
| $\bar{z}_{I}^{\text{♂}}$ | The mean PRS of male mosquitoes prior to any selection. | Internally Calculated |
| $\bar{z}_{I}^{P\text{♂}}$ | The mean PRS for parents (fathers) after insecticide selection. | Internally Calculated |
| $N^{u\text{♀}}$ | The number of female mosquitoes not encountering the insecticide. | Internally Calculated |
| $N_{i}^{E\text{♀}}$ | The number of female mosquitoes encountering and surviving the insecticide. | Internally Calculated |
| $\bar{z}_{I}^{E\text{♀}}$ | The mean PRS of females who survived the insecticide exposure. | Internally Calculated |
| $N^{P\text{♀}}$ | The total number of females becoming parents (mothers) of the next generation. | Internally Calculated |
| $N^{u\text{♂}}$ | The number of males not encountering the insecticide. | Internally Calculated |
| $N_{i}^{E\text{♂}}$ | The number of male mosquitoes encountering and surviving the insecticide. | Internally Calculated |
| $\bar{z}_{I}^{E\text{♂}}$ | The mean PRS of males who survived the insecticide exposure. | Internally Calculated |
| $N^{P\text{♂}}$ | The total number of males becoming parents (fathers) of the next generation. | Internally Calculated |
| $N^{T\text{♀}}$ | The initial total number of females in the population prior to any selection. | Internally Calculated |
| $x$ | The proportion of female mosquitoes encountering the insecticide. | Uniform(0.4 – 0.9) |
| $N^{T\text{♂}}$ | The initial total number of males in the population prior to any selection. | Internally Calculated |
| $m$ | The proportion of male mosquitoes encountering the insecticide as a proportion of the female encounter rate. | Uniform(0-1) |
| $S_{I}^{E\text{♀}}$ | The selection differential for female exposed survivors. | Internally Calculated |
| $S_{I}^{E\text{♂}}$ | The selection differential for male exposed survivors. | Internally Calculated |
| $F_{z_{I}^{E\text{♀}}}$ | The frequency of $z_{I}$ of female mosquitoes who have survived the insecticide. | Internally Calculated |
| $F_{z_{I}^{E\text{♂}}}$ | The frequency of values of $z_{I}$ of male mosquitoes who survived the insecticide. | Internally Calculated |
| $N_{ij}^{E}$ | The number of individuals encountering the mixture and surviving the exposure. | Internally Calculated |
| $N_{ij}^{E\text{♀}}$ | The number of female mosquitoes encountering the mixture and surviving. | Internally Calculated |
| $N_{ij}^{E\text{♂}}$ | The number of male mosquitoes encountering the mixture and surviving. | Internally Calculated |
| $\bar{K}_{i}^{F}$ | The mean field survival of the mosquito population to insecticide $i$. | Internally Calculated |
| $\bar{K}_{j}^{F}$ | The mean field survival of the mosquito population to insecticide $j$. | Internally Calculated |
| $\bar{z}_{I}^{E\text{♀}}$ | The mean PRS of females for Trait $I$ of individuals who survived the insecticide exposure. | Internally Calculated |
| $\phi$ | The fitness cost of the trait, as a proportion of the standard deviation. | Uniform (0.05 to 0.2) |
| $\phi^{\text{♀}}$ | The female fitness cost of the trait, as a proportion of the standard deviation. | Uniform (0.05 to 0.2). |
| $\phi^{\text{♂}}$ | The male fitness cost of the trait, as a proportion of the standard deviation. | Uniform (0.05 to 0.2) |
| $\bar{z}_{I}^{Int}$ | The initial mean PRS at the start of the generation in the intervention site. | Internally calculated |
| $\bar{z}_{I}^{Int^{'}}$ | The mean PRS of the eggs which the females from the intervention site will lay. | Internally calculated |
| $\alpha_{\Gamma I}$ | The degree of genetic correlation and the amount of cross resistance between trait $\Gamma$ and trait $I$. | -1 to 1. |
| $\bar{z}_{I}^{Ref}$ | The initial mean PRS at the start of the generation in the refugia. | Internally calculated |
| $\bar{z}_{I}^{Ref^{'}}$ | The mean PRS of the eggs which the females from the refugia will lay. | Internally calculated |
| $r_{Int}$ | The number of mosquitoes migrating from the intervention site to the refugia. | Internally calculated |
| $r_{Ref}$ | The number of mosquitoes migrating from the refugia site to the intervention site. | Internally calculated |
| $\theta$ | The dispersal rate of the populations. | Uniform(0.1 – 0.9) |
| $C$ | The proportion of the total population of mosquitoes residing in the intervention site. | Uniform(0.1 - 0.9) |
| $\bar{z}_{I}^{Int^{''}}$ | The mean PRS of eggs laid in the intervention site, i.e., the mean PRS of the next generation in the intervention site. | Internally calculated |
| $\bar{z}_{I}^{Ref^{''}}$ | The mean PRS of eggs laid in the refugia, i.e., the mean PRS of the next generation in the refugia. | Internally calculated |

| **Table C: Symbols introduced in Methods Section 3.1** | | |
| --- | --- | --- |
| Symbol | Description | Value/Range |
| $c_{i}$ | The proportion of coverage where a house is treated with only insecticide $i$. | 0-1 |
| $c_{j}$ | The proportion of coverage where a house is treated with only insecticide $j$. | 0-1 |
| $c_{ij}$ | The proportion of coverage where a house is treated with both insecticide $i$ and insecticide $j$. | 0-1 |
| $\Lambda_{i\vert ij}$ | The probability of encountering only insecticide $i$ given the mosquito entered a house with both insecticide $i$ and $j$. | 0-1 |
| $\Lambda_{j\vert ij}$ | The probability of encountering only insecticide $j$ given the mosquito entered a house with both insecticide $i$ and $j$. | 0-1 |
| $\Lambda_{ij\vert ij}$ | The probability of encountering both insecticide $i$ and $j$ given the mosquito entered a house with both insecticide $i$ and $j$. | 0-1 |
| $\Lambda_{i\vert ij}^{\text{♀}}$ | The probability of encountering only insecticide $i$ given the mosquito entered a house with both insecticide $i$ and $j$ for female mosquitoes. | 0-1 |
| $\Lambda_{j\vert ij}^{\text{♀}}$ | The probability of encountering only insecticide $j$ given the mosquito entered a house with both insecticide $i$ and $j$ for female mosquitoes. | 0-1 |
| $\Lambda_{ij\vert ij}^{\text{♀}}$ | The probability of encountering both insecticide $i$ and $j$ given the mosquito entered a house with both insecticide $i$ and $j$ for female mosquitoes. | 0-1 |
| $\Lambda_{i\vert ij}^{\text{♂}}$ | The probability of encountering only insecticide $i$ given the mosquito entered a house with both insecticide $i$ and $j$ for male mosquitoes. | 0-1 |
| $\Lambda_{j\vert ij}^{\text{♂}}$ | The probability of encountering only insecticide $j$ given the mosquito entered a house with both insecticide $i$ and $j$ for male mosquitoes. | 0-1 |
| $\Lambda_{ij\vert ij}^{\text{♂}}$ | The probability of encountering both insecticide $i$ and $j$ given the mosquito entered a house with both insecticide $i$ and $j$ for male mosquitoes. | 0-1 |
| $\rho$ | The proportion of female mosquitoes who laid eggs in the previous gonotrophic cycle surviving to start the next cycle. | Internally calculated |
| $d$ | The daily “natural” survival probability in the absence of insecticides. | 0.8 [default] [2] |
| $g$ | The length of the gonotrophic cycle in days. | 3 days [default] |

| **Table D: Symbols introduced in Methods Sections 3.2 to 3.3** | | |
| --- | --- | --- |
| Symbol | Description | Value/Range |
| $S_{I\left( G \right)}^{\text{S♀}}$ | The selection differential resulting from insecticide selection in females in cycle $G$. | Internally calculated |
| $\bar{z}_{I \left( G \right)}^{\text{P♀}}$ | The mean PRS in the population after insecticide selection in cycle $G$. | Internally calculated |
| $\bar{z}_{I \left( G=0 \right)}^{\text{♀}}$ | The mean PRS of the female prior to any selection and therefore before the start of gonotrophic cycle 1. | Internally calculated |
| $S_{I\left( G \right)}^{S\phi\text{♀}}$ | The selection differential resulting from insecticide selection and fitness costs in females in cycle $G$. | Internally calculated |
| $R_{I\left( G \right)}^{S\phi}$ | The response resulting from insecticide selection and fitness costs in cycle $G$. | Internally calculated |
| $N_{o}$ | The total number of oviposition events by all female mosquitoes in a single mosquito generation. | Internally calculated |
| $R_{I}^{T}$ | The overall response for the mosquito generation, i.e., the average between generation changes in PRS. | Internally calculated |
| $\bar{z}_{I\left( G \right)}^{P\text{♀}}$ | The mean PRS in the population after insecticide selection in cycle $G$. | Internally calculated |
| $N_{\left( G \right)}^{S\text{♀}}$ | The total number of females surviving selection, i.e., the number of females surviving insecticide encounter and the number of females not encountering the insecticide in cycle $G$. | Internally calculated |
| $N_{\left( G \right)}^{u\text{♀}}$ | The number of female mosquitoes not encountering the insecticide in cycle $G$. | Internally calculated |
| $\bar{z}_{I\left( G \right)}^{E\text{♀}}$ | The mean PRS of females for who survived the insecticide exposure in cycle $G$. | Internally calculated |
| $N_{\left( G \right)}^{\text{P}\text{♀}}$ | The number of female mosquitoes encountering and surviving the insecticide in cycle $G$ | Internally calculated |
| $F_{z_{I\left( G \right)}^{\text{♀}}}$ | The frequency of values of $z_{I}$ for all female mosquitoes in gonotrophic cycle $G$. | Internally calculated |
| $F_{z_{I\left( G \right)}^{E\text{♀}}}$ | The relative frequency of values of $z_{I}$ of female mosquitoes who have encountered and survived the insecticide in cycle $G$. | Internally calculated. |

| **Table E: Symbols introduced in Methods Section 3.4** | | |
| --- | --- | --- |
| Symbol | Description | Value/Range |
| $\bar{z}_{I \left( G=0 \right)}^{Ref\text{♀}}$ | The mean PRS of female mosquitoes hatching in the refugia prior to any selection. | Internally calculated |
| $F_{z_{I Ref \left( G \right)}^{Ref\text{♀}}}$ | The relative frequency of a value of $z_{I}$ for female mosquitoes who emerged in the refugia and lay eggs in the refugia in the cycle $G$. | Internally calculated |
| $F_{z_{I Ref \left( G \right)}^{Int \text{♀}}}$ | The relative frequency of a value of $z_{I}$ for female mosquitoes who hatched in the intervention site and lay eggs in the refugia in cycle $G$. | Internally calculated |
| $\bar{z}_{I Ref \left( G \right)}^{S Ref \text{♀}}$ | The mean PRS of female mosquitoes who hatched in the refugia and laying eggs in the refugia in the cycle $G$. | Internally calculated |
| $\bar{z}_{I Ref \left( G \right)}^{S Int \text{♀}}$ | The mean PRS of female mosquitoes who hatched in the intervention site and laying eggs in the refugia in the cycle $G$. | Internally calculated |
| $N_{Ref (G)}^{Ref \text{♀}}$ | The number of female mosquitoes who hatched (and mated) in the refugia laying eggs in the refugia in cycle $G$. | Internally calculated |
| $N_{Ref \left( G \right)}^{Int \text{♀}}$ | The number of female mosquitoes who hatched (and mated) in the intervention site laying eggs in the refugia in cycle $G$. | Internally calculated |
| $S_{I Ref \left( G \right)}^{\text{S}\text{ }Ref\text{ ♀}}$ | The insecticide selection differential of female mosquitoes who hatched in the refugia and lay eggs in the refugia in cycle $G$. | Internally calculated |
| $S_{I Ref \left( G \right)}^{\text{S}\text{ }\text{Int}\text{ ♀}}$ | The insecticide selection differential of female mosquitoes who hatched in the intervention site and lay eggs in the refugia in cycle $G$. | Internally calculated |
| $R_{I Ref \left( G \right)}^{Ref}$ | The response for trait $I$ of eggs laid by female mosquitoes who hatched (and mated) in the refugia and lay eggs in the refugia in cycle $G$. | Internally calculated |
| $R_{I Ref \left( G \right)}^{Int}$ | The response for trait $I$ of eggs laid by female mosquitoes who hatched (and mated) in the intervention site and lay eggs in the refugia in cycle $G$. | Internally calculated |
| $F_{z_{I Int \left( G \right)}^{Ref\text{♀}}}$ | The frequency of a value of $z_{I}$ for female mosquitoes who hatched in the refugia and lay eggs in the intervention site in cycle $G$. | Internally calculated |
| $F_{z_{I Int \left( G \right)}^{Int\text{♀}}}$ | The frequency of a value of $z_{I}$ for female mosquitoes who hatched in the intervention site and lay eggs in the intervention site in cycle $G$. | Internally calculated |
| $\bar{z}_{I Int \left( G \right)}^{P Ref \text{♀}}$ | The mean PRS of female mosquitoes who hatched in the refugia and laying eggs in the intervention site in the cycle $G$. | Internally calculated |
| $\bar{z}_{I Int \left( G \right)}^{P Int \text{♀}}$ | The mean PRS of female mosquitoes who hatched in the intervention site and laying eggs in the intervention site in the cycle $G$. | Internally calculated |
| $S_{I Int \left( G \right)}^{\text{S}\text{ }Ref\text{ ♀}}$ | The insecticide selection differential of female mosquitoes who hatched in the refugia and lay eggs in the intervention site in cycle $G$. | Internally calculated |
| $S_{I Int \left( G \right)}^{\text{S}\text{ }Int \text{♀}}$ | The insecticide selection differential of female mosquitoes who hatched in the intervention site and lay eggs in the intervention site in cycle $G$. | Internally calculated |
| $N_{Int (G)}^{Ref \text{♀}}$ | The number of female mosquitoes who hatched (and mated) in the refugia laying eggs in the intervention site in cycle $G$. | Internally calculated |
| $N_{Int \left( G \right)}^{Int \text{♀}}$ | The number of female mosquitoes who hatched (and mated) in the intervention site laying eggs in the intervention site in cycle $G$. | Internally calculated |
| $R_{I Int \left( G \right)}^{Ref}$ | The response of trait $I$ of eggs laid by female mosquitoes who hatched (and mated) in the refugia and lay eggs in the intervention site in cycle $G$. | Internally calculated |
| $R_{I Int \left( G \right)}^{Int}$ | The response of trait $I$ of eggs laid by female mosquitoes who hatched (and mated) in the intervention site and lay eggs in the intervention site in cycle $G$. | Internally calculated |
| $N_{o Ref}^{Total\text{♀}}$ | The total number of oviposition events in the refugia across all cycles. | Internally calculated |
| $N_{o Ref}^{Ref\text{♀}}$ | The total number of oviposition events in the refugia across all cycles for females who hatched (and mated) in refugia. | Internally calculated |
| $N_{o Ref}^{Int\text{♀}}$ | The total number of oviposition events in the refugia across all cycles for females who hatched (and mated) in the intervention site. | Internally calculated |
| $N_{o Int}^{Total\text{♀}}$ | The total number of oviposition events in the intervention site across all cycles. | Internally calculated |
| $N_{o Int}^{Int\text{♀}}$ | The total number of oviposition events in the intervention site across all cycles for females who hatched (and mated) in the intervention site. | Internally calculated |
| $N_{o Int}^{Ref \text{♀}}$ | The total number of oviposition events in the intervention site across all cycles for females who hatched (and mated) in the refugia. | Internally calculated |
| $R_{I Int}^{T Int}$ | The average overall response for trait $I$ of eggs laid in the intervention site across all cycles by females who hatched (and mated) in the intervention site. | Internally calculated |
| $R_{I Int}^{T Ref}$ | The average overall response for trait $I$ of eggs laid in the intervention site across all cycles by females who hatched (and mated) in the refugia. | Internally calculated |
| $R_{I Ref}^{T Ref}$ | The average overall response for trait $I$ of eggs laid in the refugia across all cycles by females who hatched (and mated) in the refugia. | Internally calculated |
| $R_{I Ref}^{T Int}$ | The average overall response for trait $I$ of eggs laid in the refugia across all cycles by females who hatched (and mated) in the intervention site. | Internally calculated |

**References**

1. Hobbs N, Weetman D, Hastings I. Insecticide resistance management strategies for public health control of mosquitoes exhibiting polygenic resistance: a comparison of sequences, rotations, and mixtures. Evolutionary Applications. 2023;16: 936–959. doi:DOI: 10.1111/eva.13546

2. Matthews J, Bethel A, Osei G. An overview of malarial Anopheles mosquito survival estimates in relation to methodology. Parasites and Vectors. 2020;13: 1–12. doi:10.1186/s13071-020-04092-4
