# Supplementary material for: Simulating dynamic insecticide selection pressures for resistance management in mosquitoes assuming polygenic resistance
Source: PLoS Comput Biol. 2025 Apr 28;21(4):e1012944. doi: 10.1371/journal.pcbi.1012944 (PMC12058183; doi:10.1371/journal.pcbi.1012944)
Supplement: S2 File — (DOCX) [file pcbi.1012944.s002.docx]

**S2 File: Calibration of the Exposure Scaling Factor (**$\boldsymbol{\beta}$**) for “novel” insecticides**

As previously described for the “polyres” model, the model is calibrated such that, on average, a novel insecticide would be expected to last approximately 10 years under continuous deployment in the absence of fitness costs and refugia [1]; this is achieved using $\beta$ in Equation 1b. With the dynamic models described here (“polytruncate” and “polysmooth”) there are two values requiring calibration. First, the standard deviation ($\sigma_{I}$) of the mean PRS ($\bar{z}_{I}$), and second the exposure scaling factor ($\beta$). The standard deviation for novel insecticides ($\bar{z}_{I}$=0 to $\bar{z}_{I}$=100) is input as values as estimated (see S3 File), where plausible range of $\sigma_{I}$ = 20 to $\sigma_{I}$ = 80. The exposure scaling factor ($\beta$) is a factor converting the selection to our desired timescale and is used to account for uncertainty in the value of selection differentials and heritability.

**Beta and Standard Deviation Calibration Simulations**

Parameters were sampled using Latin hypercube sampling [2] within uniform distributions. A total of 5000 parameter sets for values of female exposure (0.4-0.9), male exposure (0-1) and heritability (0.05 – 0.30) were used. Coverage was set to 1, dispersal is therefore absent (if coverage is 1, there is no refugia) and fitness costs were set to zero. No insecticide decay was allowed, and the insecticide efficacy ($\omega_{\tau}^{i}$) remained at 1 for the duration of the simulation. These same 5000 parameter sets were used for each standard deviation and exposure scaling factor permutation, and for both the “polytruncate” and “polysmooth” model. Simulations were run with a single insecticide deployed in sequence. The withdrawal threshold was 10% bioassay survival. The outcome measured was time (in generations) to 10% bioassay survival, defined as the “operational lifespan” of the insecticide.

**Beta and Standard Deviation Calibration Results**

The permutation of exposure scaling factor and standard deviation which gave an average insecticide lifespan of ~10 years (within the 8-12 year range) was found to be standard deviation = 50 and exposure scaling factor = 10 for “polysmooth” (Fig A in S2 File), and for “polytruncate” was found to be standard deviation = 20 and exposure scaling factor = 1 (Fig B in S2 File). It should be noted the exposure scaling factor for the “polysmooth” model was identified to be 10, the same value as used for the calibration of the previously published “polyres” model[1].


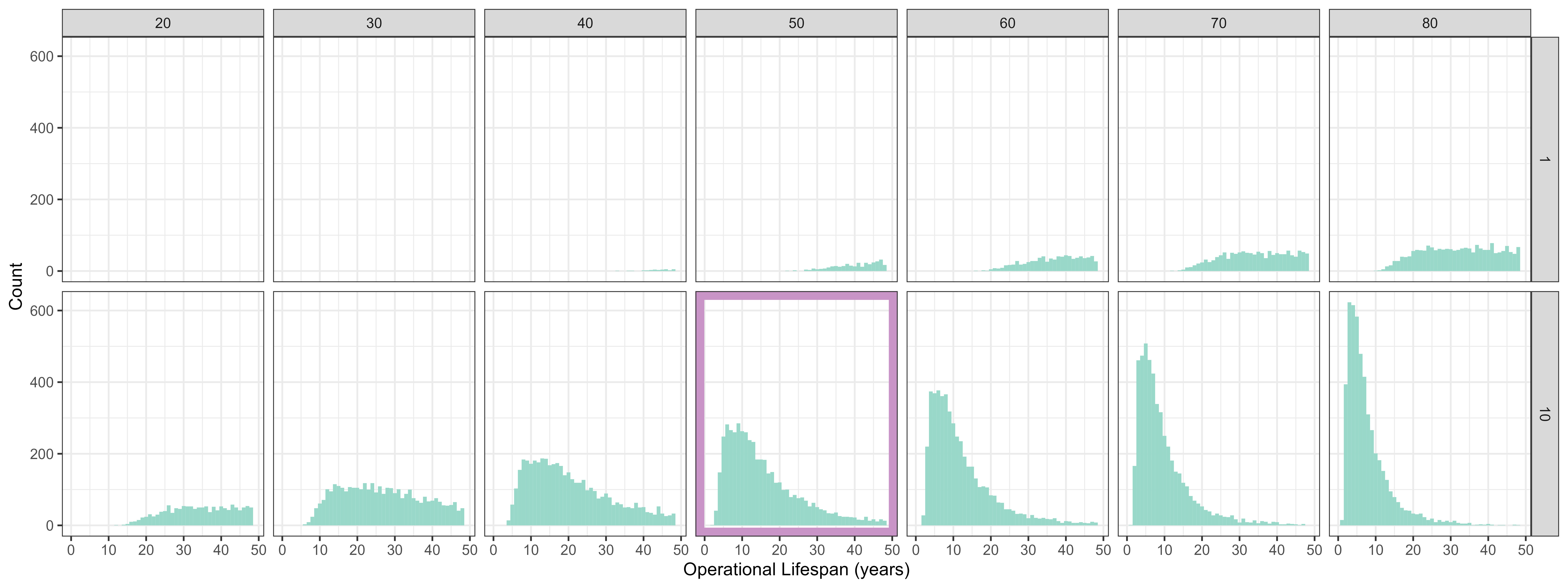


**Fig A: Polysmooth Calibration with Exposure Scaling Factor and Standard Deviation**. Each row of plots indicates the exposure scaling factor used (i.e., 1 or 10). Each column of the plots indicates the standard deviation used (i.e., 20 to 80). A standard deviation of 50 and an exposure scaling factor of 10 was found to best calibrate the “polysmooth” model, highlighted in purple.


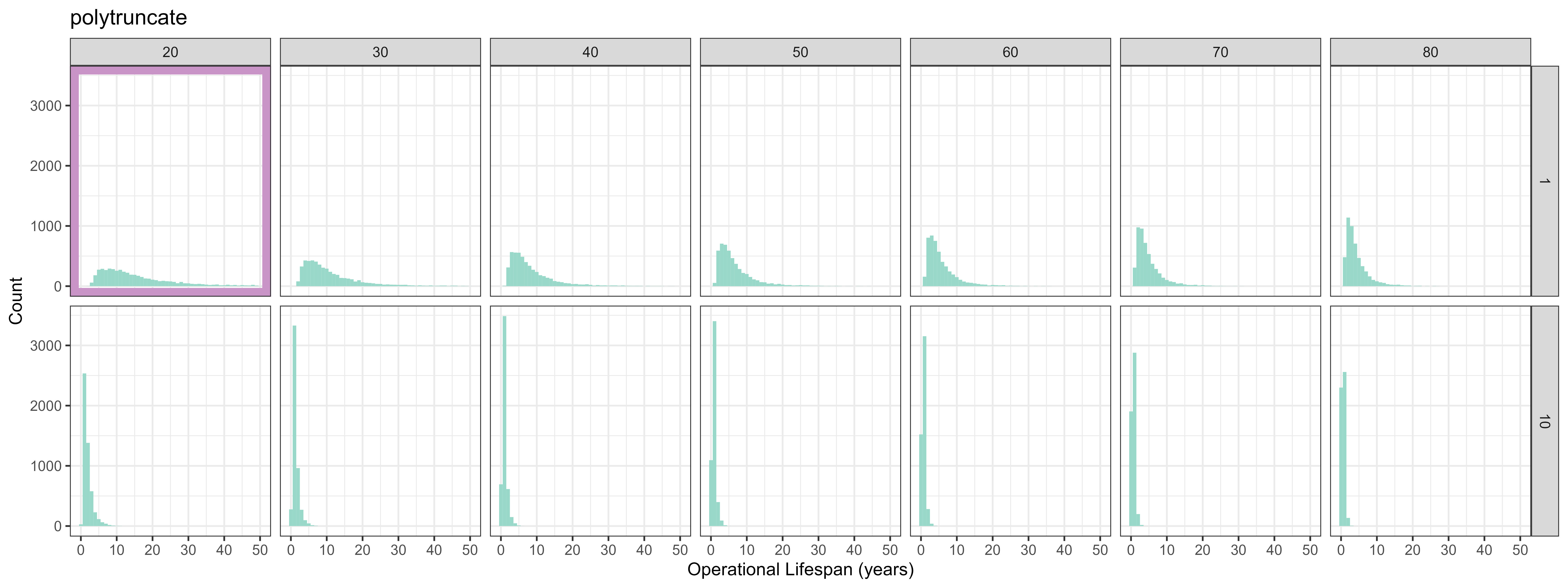


**Fig B: Polytruncate Calibration with Exposure Scaling Factor and Standard Deviation.** Plot structure is as for Fig A in S2. The plot highlighted in purple indicates that a standard deviation of 20 and an exposure scaling factor of 1 was found to best calibrate the “polytruncate” model.

**References:**

1. Hobbs N, Weetman D, Hastings I. Insecticide resistance management strategies for public health control of mosquitoes exhibiting polygenic resistance: a comparison of sequences, rotations, and mixtures. Evolutionary Applications. 2023;16: 936–959. doi:DOI: 10.1111/eva.13546

2. Carnell R. lhs: Latin Hypercube Samples. 2020.
