# Supplementary material for: Simulating dynamic insecticide selection pressures for resistance management in mosquitoes assuming polygenic resistance
Source: PLoS Comput Biol. 2025 Apr 28;21(4):e1012944. doi: 10.1371/journal.pcbi.1012944 (PMC12058183; doi:10.1371/journal.pcbi.1012944)
Supplement: S3 File — (DOCX) [file pcbi.1012944.s003.docx]

**S3 File: Fixed and Variable Standard Deviations**

This supplement explains the rationale behind allowing the standard deviation to be fixed or to scale with the population mean value of the Polygenic Resistance Score (PRS) (Methods Section 1.4). It also provides details of the estimation of the value of the population standard deviation ($\sigma_{I}$).

**Estimating the Population Standard Deviation (**$\boldsymbol{\sigma}_{\boldsymbol{I}}$**) of the Mean Polygenic Resistance Score (**${\bar{\boldsymbol{z}}}_{\boldsymbol{I}}$**) from Field Data**

The “polytruncate” and “polysmooth” models require a manual input of the standard deviation of the mean PRS of the population ($\sigma_{I}$). The first step is to therefore calculate the standard deviation (of the PRS) from WHO bioassay results. This required identifying papers which reported both the mean bioassay mortality and the associated 95% confidence interval of this estimate. Note that the WHO dataset of bioassay results (the Malaria Threat Map) only includes the point estimate of the bioassay mortality and does not include the 95% confidence intervals.

Publications were included if they met the following three requirements: 1). reported the mean bioassay mortality; and 2). reported the 95% confidence interval (CI) of the estimate; 3). Reported the number of bioassays or number of mosquitoes. For publications where the number of mosquitoes was given instead of the number of bioassays, we estimated the expected number of bioassays as number of mosquitoes divided by 25; and rounded to the nearest whole number. The number of mosquitoes was divided by 25 as this is the recommended number of mosquitoes per cylinder bioassay [1]. Data meeting these requirements was obtained and extracted from WHO cylinder bioassay results from Uganda [2], Democratic Republic of the Congo [3] and Ethiopia [4]. Data was from *Anopheles gambiae* s.l. and *Anopheles arabiensis* and included a wide variety of insecticides.

The reported mean bioassay mortality and 95% CI was concerted to the corresponding bioassay survival (survival = 1 - mortality). These survival values were then converted to the PRS (using Equation 1a). The standard deviation of the PRS can then be calculated:

$$\sigma_{i}= \sqrt{N}*(Upper 95\% CI -Lower 95\%CI)/3.92$$

 Equation S3

Where $N$ is the number of WHO cylinder bioassays conducted (or estimated to have been conducted).

One of the major purposes of the model is to evaluate how to deploy novel (or near novel) insecticides to extend their operational lifespan. Therefore, we restricted the analysis of publications to include only those where the bioassay survival was less than 10%. Values for the standard deviation of the PRS for novel (or novel-like) insecticides (i.e., have a mean PRS ($\bar{z}_{I}$) of between 0 and 100) were typically in the range of 20 to 80 (Fig A in S3 File), so these values are used to calibrate the exposure scaling factor ($\beta$) (see S2 File).

The standard deviation varies with the magnitude of resistance in the population (Fig B in S3 File). This is an important point, especially when considering mixtures, because where the two insecticides in the mixture may have substantially different levels of resistance. We therefore constructed a linear model of standard deviation ~ mean PRS (Equation 2e). Values were limited to PRS values less than 3600 (80% bioassay survival). The results of the linear model are given in Table A in S3 File. We use an intercept value of 18 and regression coefficient value of 0.4 in our simulations.

| **Table A: Linear Model Mean PRS and Standard Deviation** | | | | |
| --- | --- | --- | --- | --- |
|  | Estimate | Lower 95% CI | Upper 95% CI | p value |
| Intercept | 18.19349 | -1.9884385 | 38.3754220 | 0.0768 |
| Mean PRS | 0.40220 | 0.3822935 | 0.4221146 | <2e-16 |
| Residual standard error: 98.43 on 127 degrees of freedom  Multiple R-squared: 0.9264, Adjusted R-squared: 0.9258  F-statistic: 1598 on 1 and 127 DF, p-value: < 2.2e-16 | | | | |


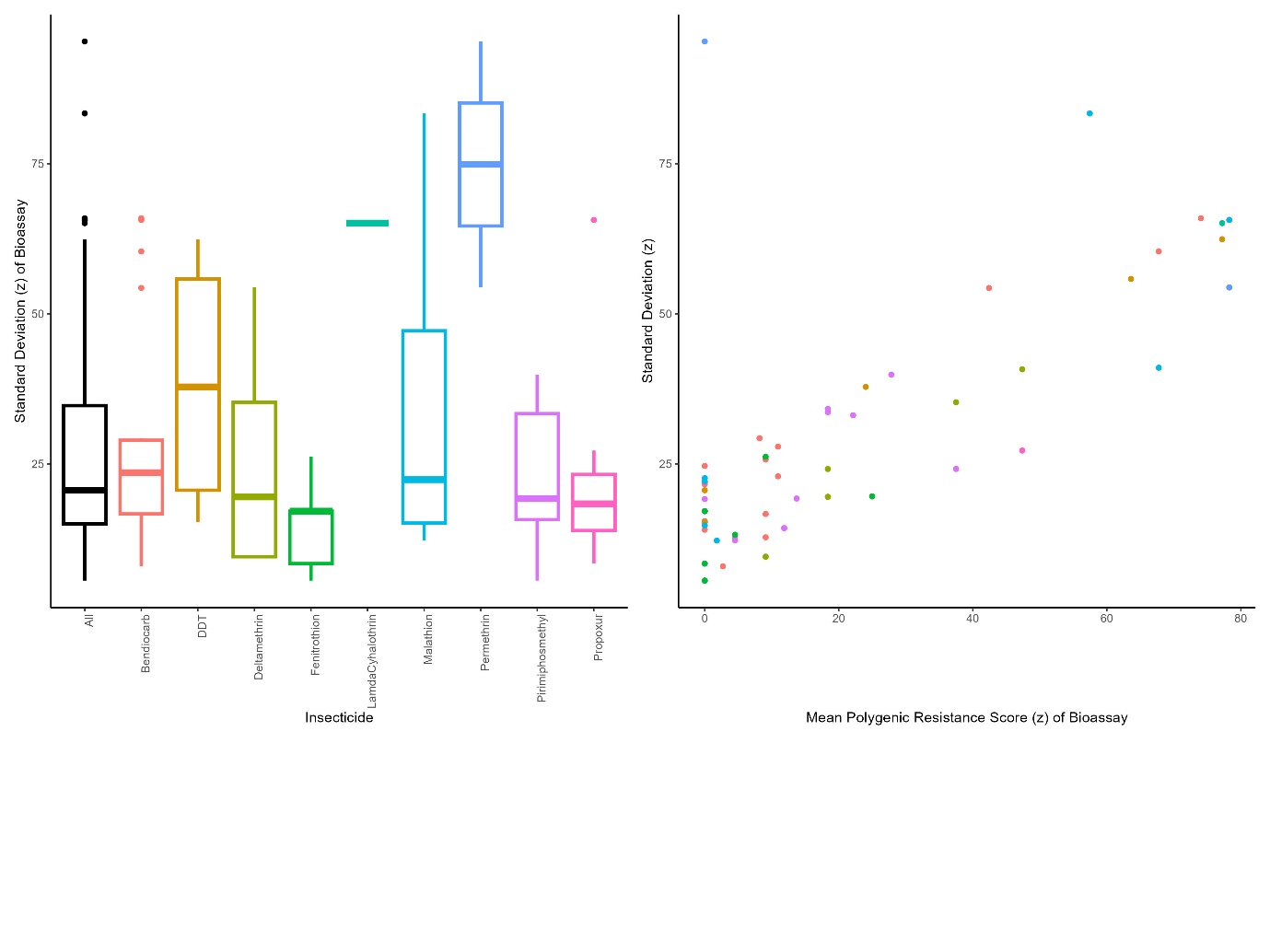


**Fig A: Standard deviation of Polygenic Resistance Score at 10% Bioassay Survival.** Left panel: Boxplot of the standard deviation of the calculated mean polygenic resistance score for all (black) and individual insecticides (colours). Right panel: scatterplot of the relationship between the mean PRS value and its< corresponding calculated standard deviation.


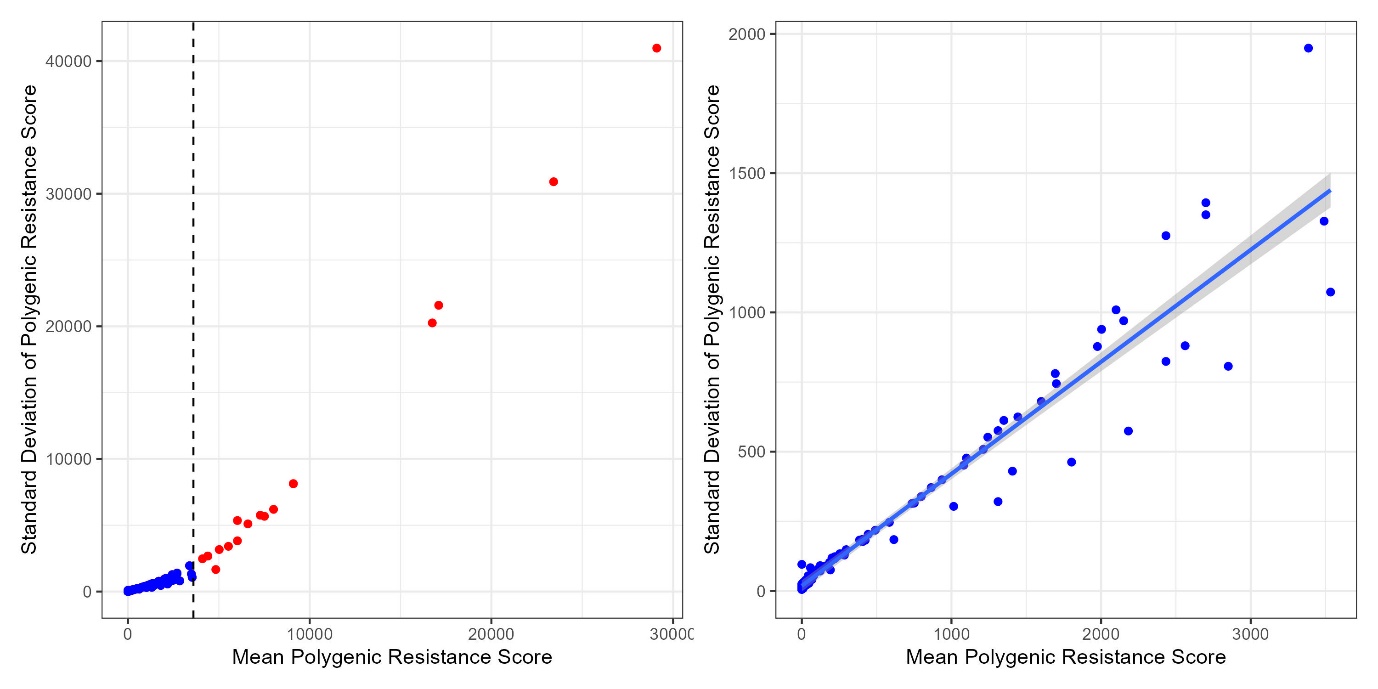


**Fig B: Relationship between Mean Polygenic Resistance Score and Standard Deviation.** Left Panel: Relationship for all PRS values. Right Panel: PRS values limited to 0-3600 (0-80% bioassay survival), with fitted linear model (and 95% CI).

**References**

1. WHO. Test procedures for insecticide resistance monitoring in malaria vector mosquitoes (Second edition) (Updated June 2018). Who. Geneva; 2018.

2. Thomsen EK, Strode C, Hemmings K, Hughes AJ, Chanda E, Musapa M, et al. Underpinning sustainable vector control through informed insecticide resistance management. PLoS ONE. 2014;9. doi:10.1371/journal.pone.0099822

3. Wat’Senga F, Manzambi EZ, Lunkula A, Mulumbu R, Mampangulu T, Lobo N, et al. Nationwide insecticide resistance status and biting behaviour of malaria vector species in the Democratic Republic of Congo. Malaria Journal. 2018;17: 1–13. doi:10.1186/s12936-018-2285-6

4. Alemayehu E, Asale A, Eba K, Getahun K, Tushune K, Bryon A, et al. Mapping insecticide resistance and characterization of resistance mechanisms in Anopheles arabiensis (Diptera: Culicidae) in Ethiopia. Parasites and Vectors. 2017;10: 1–11. doi:10.1186/s13071-017-2342-y
