# Supplementary material for: Simulating dynamic insecticide selection pressures for resistance management in mosquitoes assuming polygenic resistance
Source: PLoS Comput Biol. 2025 Apr 28;21(4):e1012944. doi: 10.1371/journal.pcbi.1012944 (PMC12058183; doi:10.1371/journal.pcbi.1012944)
Supplement: S4 File — (DOCX) [file pcbi.1012944.s004.docx]

**S4 File: Fitness cost Selection Differential Values:**

In this supplement we provide details of the two options for implementing fitness costs and their associated parameter estimates.

**Option 1 Parameter Estimation: Fixed Fitness Cost Selection Differential.**

When the standard deviation is constant in the simulation, the fitness cost selection differential is user defined as a fixed value. In the previous model version (polyres), fitness costs were calculated as a proportion of the between generation response (Hobbs et al., 2023). The response was constant in “polyres” and this approach ensure fitness costs were lower than the response (guaranteeing resistance will spread). However, in the dynamic models developed here, the response varies depending on the level of resistance in the population and is therefore not fixed throughout the simulation.

For the “polyres” model the fitness costs response (Equation 5 in Hobbs et al., 2023) was assumed to lie between a maximum value calculated as:

$$fitness cost response=-0.2\left( 10\frac{0.3*0.9*\left( 1+1 \right)}{2} \right)=0.174$$

and a minimum value calculated as:

$$fitness cost response=-0.01\left( 10\frac{0.05*0.4*\left( 1+0 \right)}{2} \right)=0.002$$

This was calculated assuming male and female fitness costs were identical ($S_{i}^{\phi\text{♀}}= S_{i}^{\phi\text{♂}}$) and $h_{I}^{2}$ is maximally 0.3 and minimally 0.05. These values can be input into Equation 3c in the presented manuscript. The minimum and maximum fitness cost selection differentials for the dynamic model are therefore $S_{i}^{\phi}=-0.58$ and $S_{i}^{\phi}=-0.04$.

**Option 2 Parameter Estimation: Fitness selection differentials vary with** $\boldsymbol{\sigma}_{\boldsymbol{I}}$ **and** ${\bar{\boldsymbol{z}}}_{\boldsymbol{I}}$

In this option, the fitness cost selection differential varies with the standard deviation. $S_{I}^{\phi}$ is minimally and maximally at $-0.58$ and $-0.04$, for a fixed standard deviation. When $\bar{z}_{I}$=0, then $\sigma_{I}$=18 (Equation 2e). Therefore, using Equation S4.1, $\phi$ is maximally 0.0322 and minimally 0.0022 when allowing the fitness costs to vary with the magnitude of the mean resistance of the population. We set the fitness costs to be a fixed proportion of the standard deviation, allowing the fitness costs selection differential to increase with an increasing $\bar{z}_{I}$ and therefore increasing $\sigma_{I}$.

$$S_{I}^{\phi}=\phi\sigma_{I}$$

 Equation S4.1

As it may be expected for fitness costs differentially affect males and females, we can separately calculate the sex-specific fitness cost selection differentials.

$$S_{I}^{\phi\text{♀}}=\phi^{\text{♀}}\sigma_{I}$$

 Equation S4.1($\text{♀}$)

$$S_{I}^{\phi\text{♂}}=\phi^{\text{♂}}\sigma_{I}$$

 Equation S4.1($\text{♂}$)

By manually inputting the fitness selection differentials (option 1 or option 2), it may be possible for some simulations to never have IR spread if the fitness cost selection differential is higher than selection differential brought about by insecticide selection ($S_{I}^{\phi\text{♀}}$ and $S_{I}^{\phi\text{♂}}$ are greater than $S_{I}^{S\text{♀}}$ and $S_{I}^{S\text{♂}}$). These would be situations where insecticide selection is low (e.g., low coverage and low exposure), and in such situations the selection pressure would be low anyway such that even in the absence of any fitness cost resistance would be expected to be very slow to build up anyway. And of course, in situations where IR cannot take off, there is no need for any IRM as IR does not become a problem.

**References**

Hobbs N, Weetman D, Hastings I. Insecticide resistance management strategies for public health control of mosquitoes exhibiting polygenic resistance: a comparison of sequences, rotations, and mixtures. Evolutionary Applications. 2023;16: 936–959. doi:DOI: 10.1111/eva.13546
