# Supplementary material for: Simulating dynamic insecticide selection pressures for resistance management in mosquitoes assuming polygenic resistance
Source: PLoS Comput Biol. 2025 Apr 28;21(4):e1012944. doi: 10.1371/journal.pcbi.1012944 (PMC12058183; doi:10.1371/journal.pcbi.1012944)
Supplement: S5 File — (DOCX) [file pcbi.1012944.s005.docx]

**S5 File: Calculating the Male Insecticide Selection Differential with Complex Insecticide Encounters:**

Male mosquitoes may also contact complex insecticide deployments such as combinations or micro-mosaics. This will impact the level of selection on male mosquitoes and therefore impact the male insecticide selection differential. This can be included in the model by updating Equation 4b($\text{♂}$), to allow male mosquitoes to encounter just insecticide $i$, just insecticide $j$ or both insecticide $i$and $j$. The mean PRS of the male mosquitoes in the parental population ($\bar{z}_{I}^{P\text{♂}}$) is calculated from all insecticide encounters, where the superscript “$P$” indicates the parental population. The superscript “$E$” indicates the mosquitoes survived their encounter with the insecticide(s) and the superscript “$u$” indicates those males did not encounter the insecticide.

$$\bar{z}_{I}^{P\text{♂}}= \frac{\left( \left( N_{i}^{E\text{♂}}\bar{z}_{I}^{E\text{♂}} \right)+\left( N_{j}^{E\text{♂}}\bar{z}_{I}^{\text{♂}} \right)+\left( N_{ij}^{E\text{♂}}\bar{z}_{I}^{E\text{♂}} \right) +\left( N^{u\text{♂}} \bar{z}_{I}^{\text{♂}} \right) \right)}{N^{P\text{♂}}}$$

 Equation 12a($\text{♂}$)

The total number of males is then:

$$N^{P\text{♂}}= N_{i}^{E\text{♂}}+ N_{j}^{E\text{♂}}+N_{ij}^{E\text{♂}} +N^{u\text{♂}}$$

Equation 12b($\text{♂}$)

The number of males surviving the encounter only with insecticide $i$ ($N_{i}^{E\text{♂}}$) is calculated from two groups: (i) those entering houses with insecticide $i$ only and surviving, (ii) those entering houses with both insecticide $i$ and $j$ but contacting only insecticide $i$ and surviving.

$$N_{i}^{E\text{♂}}=\left( c_{i}xm\bar{K}_{i}^{F}N^{T\text{♂}} \right)+\left( c_{ij}\Lambda_{i|ij}^{\text{♂}}xm\bar{K}_{i}^{F}N^{T\text{♂}} \right)$$

 Equation 12c($\text{♂}$)

Similarly for encountering only insecticide $j$:

$$N_{j}^{E\text{♂}}=\left( c_{j}xm\bar{K}_{j}^{F}N^{T\text{♂}} \right)+ \left( c_{ij}\Lambda_{j|ij}^{\text{♂}}xm\bar{K}_{j}^{F}N^{T\text{♂}} \right)$$

 Equation 12d($\text{♂}$)

The number of males surviving the encounter with both insecticides is the number of males entering a house with both insecticides, encountering both insecticides, and surviving both insecticides.

$$N_{ij}^{E\text{♂}}=c_{ij}\Lambda_{ij|ij}^{\text{♂}}xm\bar{K}_{i}^{F}\bar{K}_{j}^{F}N^{T\text{♂}}$$

 Equation 12e($\text{♂}$)

The number of males not encountering insecticides:

$$N^{u\text{♂}}=N^{T\text{♂}}\left( 1-xm \right)$$

 Equation 12f($\text{♂}$)

The value of $F_{z_{I}^{E\text{♂}}}$ is calculated for those encountering insecticide $i$:

$F_{z_{I}^{E\text{♂}}}=\left( F_{z_{I}^{\text{♂}}}K_{i}^{F}xmc_{i} \right)+ \left( {F_{z_{I}^{\text{♂}}}c}_{ij}\Lambda_{i|ij}^{\text{♂}}xmK_{i}^{F} \right)+ \left( F_{z_{I}^{\text{♂}}}c_{ij}\Lambda_{ij|ij}^{\text{♂}}xmK_{i}^{F}\bar{K}_{j}^{F} \right)$

Equation 12g($\text{♂}$)

The values of $F_{z_{I}^{E\text{♂}}}$ can then be used to calculate the mean PRS of the exposed surviving males:

$$\bar{z}_{I}^{E\text{♂}}=\left( \sum_{z_{I}^{\text{♂}}= -\infty}^{\infty} F_{z_{I}^{E\text{♂}}}z_{I}^{\text{♂}} \right) / {(N}_{i}^{E\text{♂}}+N_{ij}^{E\text{♂}})$$

 Equation 12h($\text{♂}$)

$\bar{z}_{I}^{E\text{♂}}$ is returned to Equation 12a($\text{♂}$) to calculate $\bar{z}_{I}^{P\text{♂}}$ which is required to calculate the male insecticide selection differential (Equation 4a($\text{♂}$)) which can be combined with fitness costs to finally be incorporated into the sex-specific Breeder's equation allowing for multiple gonotrophic cycles (Equation 11b) to allow for the calculation of the response.
