# Supplementary material for: Simulating dynamic insecticide selection pressures for resistance management in mosquitoes assuming polygenic resistance
Source: PLoS Comput Biol. 2025 Apr 28;21(4):e1012944. doi: 10.1371/journal.pcbi.1012944 (PMC12058183; doi:10.1371/journal.pcbi.1012944)
Supplement: S6 File — (DOCX) [file pcbi.1012944.s006.docx]

**S6 File: Multiple Gonotrophic Cycles and Dispersal.**

Firstly, recall that female mosquitoes mate only once and the stored sperm is used to fertilize eggs in all subsequent gonotrophic cycles. Mating occurs within their area of hatching (intervention or refugia) and non-overlapping generations are assumed. Consequently, male dispersal does not to be tracked.

A conceptual description of the multiple gonotrophic cycle model with dispersal is provided in Fig A in S6 File. A graphical guide to notation is in Fig B in S6 File.


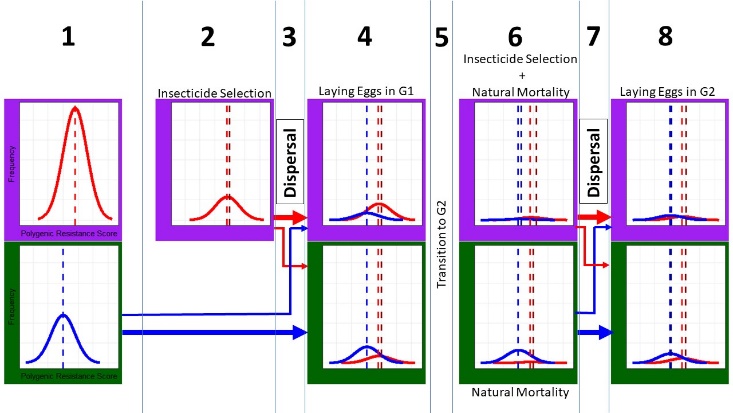


**Fig A: Diagrammatic Representation of Multiple Dispersal Events with Multiple Gonotrophic Cycles.** The curves show the distributions of the polygenic resistance scores (PRS) in each site at each time point. Left to Right: **1):** Females emerge in the intervention site (purple background) and refugia (green background). **2):** Selection occurs. Insecticide selection occurs only in the intervention site. Fitness costs occur both in the intervention site and refugia. After insecticide selection the mosquitoes mate. Males and females which emerged in the intervention site mate together. Males and females which emerged in the refugia mate together. **3):** Females can disperse between the two sites. **4):** Females laying eggs in the intervention site (purple background) are the remaining females from the intervention site (red) and the females joining from the refugia (blue). Laying eggs in the refugia (green background) are females remaining (blue) and those joining from the intervention site (red). **5):** The second gonotrophic cycle starts. **6):** Females in the intervention site (purple background), consist of both the “intervention” females (red) and the “refugia” females (blue) and undergo both insecticide selection and natural mortality. Females in the refugia (green background) consist of both the “refugia” females (blue) and the “intervention” females (red) and undergo only natural mortality. **7):** The female mosquitoes again disperse between the two sites. **8):** The female mosquitoes then lay eggs in their site of residence (intervention or refugia). Subsequent gonotrophic cycles (i.e., #3 onwards) follow the same process until the maximum number of gonotrophic cycles is reached. The light coloured vertical lines show initial mean PRS at hatching. The darker coloured vertical lines show the current mean PRS in the current location after fitness costs (both sites) and insecticide selection (intervention site). The difference between these lines are therefore the female insecticide selection differential in each site.


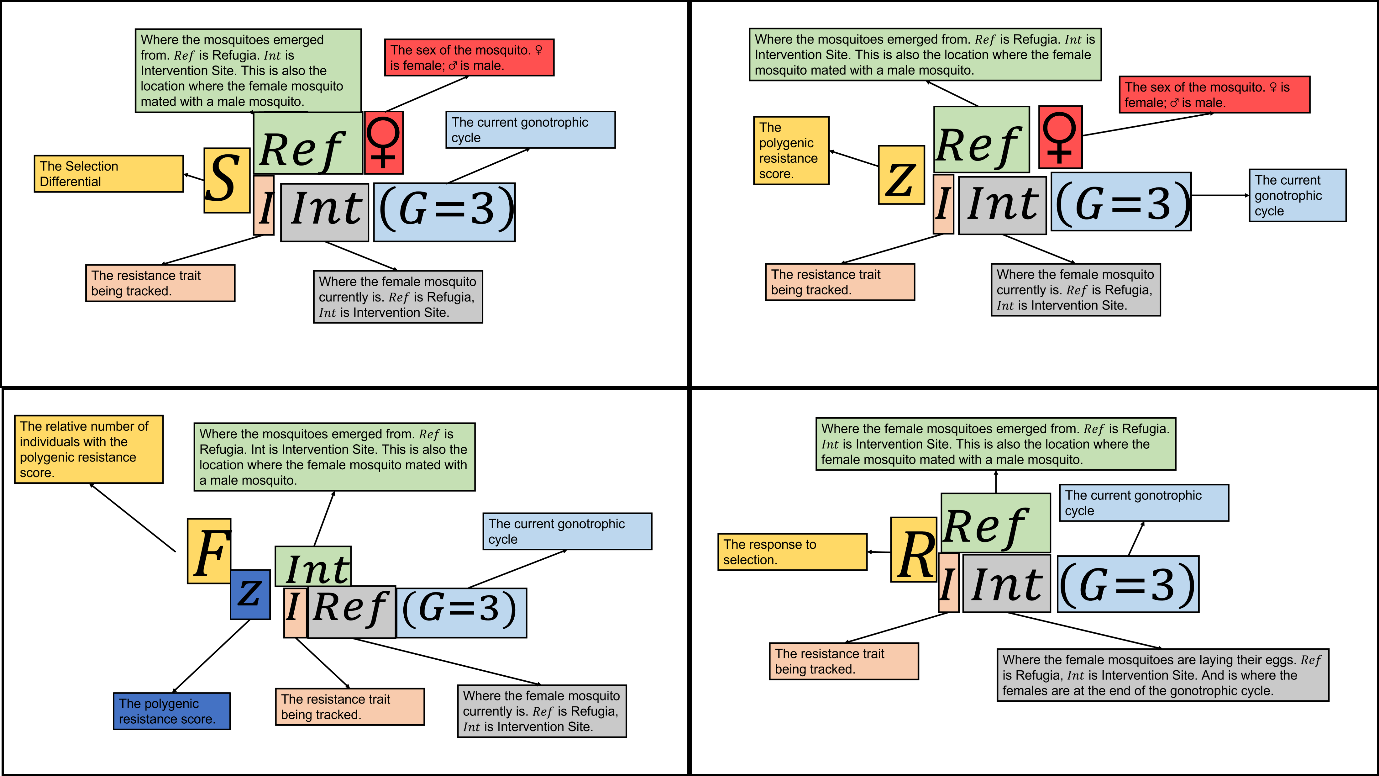


**Fig B:** **Structure of the Mathematical Symbols Used for Tracking Multiple Gonotrophic Cycles with Dispersal.** The coding is explained on the diagram; for example (i) superscripts in green represent where the mosquitoes hatched (and for females this also indicates where they mated). $Int$ is the intervention site, and $Ref$ is the refugia. Subscripts in light grey use the same code ($Int$ or $Ref$) refer to the current location of the mosquito (i.e., where it is encountering a selection pressure or laying eggs).

**Equations for Gonotrophic cycle 1: Laying Eggs in the Refugia**

The frequency of females with a PRS of $z_{I}^{\text{♀}}$ laying eggs in the refugia in the first cycle consists of (1) those who hatch, mate and stay in the refugia ($F_{z_{I Ref \left( G=1 \right)}^{Ref\text{♀}}}$) and (ii) those who hatch and mate in the intervention site (undergoing insecticide selection) then disperse to the refugia ($F_{z_{I Ref \left( G=1 \right)}^{Int \text{♀}}}$):

$$F_{z_{I Ref \left( G=1 \right)}^{Ref\text{♀}}}=\left( F_{z_{I Ref\left( G=0 \right)}^{Ref\text{♀}}}*\left( 1-C \right) \right)*\left( 1-\theta\right)$$

 Equation 14a(i)(Ref)

$$F_{z_{I Ref \left( G=1 \right)}^{Int \text{♀}}}=\left( \left( F_{z_{I Int \left( G=0 \right)}^{Int \text{♀}}}Cc_{i}xK_{i}^{F} \right)+\left( F_{z_{I Int \left( G=0 \right)}^{Int \text{♀}}}Cc_{j}x \bar{K}_{j}^{F} \right)+ \left( F_{z_{I Int \left( G=0 \right)}^{Int \text{♀}}}Cc_{ij}\Lambda_{j|ij}^{\text{♀}}x \bar{K}_{j}^{F} \right)+ \left( F_{z_{I Int \left( G=0 \right)}^{Int \text{♀}}}Cc_{ij}\Lambda_{i|ij}^{\text{♀}}xK_{i}^{F} \right)+ \left( F_{z_{I Int \left( G=0 \right)}^{Int \text{♀}}}Cc_{ij}\Lambda_{ij|ij}^{\text{♀}}xK_{i}^{F}\bar{K}_{j}^{F} \right)+ \left( F_{z_{I Int \left( G= 0 \right)}^{Int \text{♀}}}C*\left( 1-x \right) \right) \right)* \theta$$

Equation 14b(i) (Ref)

The term $C$ scales the intervention and refugia population sizes by the coverage and is included only in the first cycle. $F_{z_{I \left( G=0 \right)}^{Ref\text{♀}}}$ and $F_{z_{I Int \left( G= 0 \right)}^{Int \text{♀}}}$are calculated from Equation 4g($\text{♀}$). $K_{i}^{F}$ depends on insecticide $i$ efficacy. $\bar{K}_{j}^{F}$ depends on $\bar{z}_{J Int \left( G=0 \right)}^{Int \text{♀}}$ and of insecticide $j$ efficacy (Equation 2b(i)).

The total number of females laying eggs in the refugia in each cycle consists of (i) those who originally hatched, mated and stayed in the refugia ($N_{Ref (G)}^{Ref \text{♀}}$) and (ii) those who hatched and mated in the intervention site but dispersed to the refugia ($N_{Ref \left( G \right)}^{Int \text{♀}}$):

$$N_{Ref (G)}^{Ref \text{♀}}= \sum_{z_{I Ref}^{Ref\text{♀}}= -\infty}^{\infty} F_{z_{I Ref \left( G \right)}^{Ref\text{♀}}}$$

 Equation 14c(i) (Ref)

$$N_{Ref \left( G \right)}^{Int \text{♀}}= \sum_{z_{I Ref}^{Int \text{♀}}= -\infty}^{\infty} F_{z_{I Ref \left( G \right)}^{Int \text{♀}}}$$

 Equation 14d(i) (Ref)

The mean PRS for females each cycle is calculated depending on their hatching site. For those who hatched in the refugia:

$$\bar{z}_{I Ref \left( G \right)}^{P Ref \text{♀}}=\left( \sum_{z_{I Ref \left( G \right)}^{Ref\text{♀}}= -\infty}^{\infty} F_{z_{I Ref \left( G \right)}^{Ref\text{♀}}}z_{I \left( G=0 \right)}^{Ref\text{♀}} \right)/N_{Ref (G)}^{Ref \text{♀}}$$

 Equation 14e(i) (Ref)

And those who hatched in the intervention site:

$$\bar{z}_{I Ref \left( G \right)}^{P Int \text{♀}}=\left( \sum_{z_{I Ref \left( G \right)}^{Int \text{♀}}= -\infty}^{\infty} F_{z_{I Ref \left( G \right)}^{Int\text{♀}}}z_{I \left( G=0 \right)}^{Int\text{♀}} \right)/N_{Ref (G)}^{Int\text{♀}}$$

 Equation 14f(i) (Ref)

Female insecticide selection differentials in the refugia can be written for any cycle as:

$$S_{I Ref \left( G \right)}^{\text{ }\text{S}\text{ }Ref\text{ ♀}}=\bar{z}_{I Ref \left( G \right)}^{PRef\text{♀}}- \bar{z}_{I \left( G=0 \right)}^{Ref \text{♀}}$$

 Equation 14g(i) (Ref)

$$S_{I Ref \left( G \right)}^{\text{S}\text{ }Int \text{♀}}=\bar{z}_{I Ref \left( G \right)}^{P Int \text{♀}}- \bar{z}_{I \left( G=0 \right)}^{Int\text{♀}}$$

 Equation 14h(i) (Ref)

This is combined with the fitness cost selection differential (Equation 11a(ii)) to give the overall selection differential. The responses are then calculated separately. For females which hatched, mated and stayed in the refugia ($R_{I Ref \left( G \right)}^{Ref}$):

$$R_{I Ref \left( G \right)}^{Ref}= h^{2}\left( \frac{S_{I Ref \left( G \right)}^{Ref \Phi\text{♀}}+ S_{I}^{Ref \Phi\text{♂}}}{2} \right)\beta$$

 Equation 14i(i) (Ref)

And for females who hatched and mated in the intervention site and dispersed to the refugia ($R_{I Ref \left( G \right)}^{Int}$):

$$R_{I Ref \left( G \right)}^{Int}= h^{2}\left( \frac{S_{I Ref \left( G \right)}^{Int S\Phi\text{♀}}+ S_{I}^{Int S\Phi\text{ ♂}}}{2} \right)\beta$$

Equation 14j(i) (Ref)

**Gonotrophic cycle 1: Laying Eggs in the Intervention Site**

A parallel process occurs for the intervention site. The number of females laying eggs in the intervention site in the first cycle consists of (i) those who hatched hatched and mated in the refugia but dispersed to the intervention site ($F_{z_{I Int\left( G=1 \right)}^{Ref\text{♀}}}$) and (ii) those who hatched, mated and stayed in the intervention site (undergoing insecticide selection) ($F_{z_{I Int\left( G=1 \right)}^{Int\text{♀}}}$):

$$F_{z_{I Int\left( G=1 \right)}^{Ref\text{♀}}}= F_{z_{I Ref\left( G=0 \right)}^{\text{ }Ref \text{♀}}}\theta*\left( 1-C \right)$$

 Equation 14a(i) (Int)

$$F_{z_{I Int\left( G=1 \right)}^{Int\text{♀}}}=\left( \left( F_{z_{I Int\left( G=0 \right)}^{Int \text{♀}}}Cc_{i}x K_{i}^{F} \right)+\left( F_{z_{I Int\left( G=0 \right)}^{Int \text{♀}}}C c_{j}x\bar{K}_{j}^{F} \right)+\left( F_{z_{I Int\left( G=0 \right)}^{Int \text{♀}}}C c_{ij}\Lambda_{i|ij}^{\text{♀}}xK_{i}^{F} \right)+\left( F_{z_{I Int\left( G=0 \right)}^{Int \text{♀}}}C c_{ij}\Lambda_{j|ij}^{\text{♀}}x\bar{K}_{j}^{F} \right)+\left( F_{z_{I Int\left( G=0 \right)}^{Int \text{♀}}}C c_{ij}\Lambda_{ij|ij}^{\text{♀}}xK_{i}^{F}\bar{K}_{j}^{F} \right) +\left( F_{z_{I Int\left( G=0 \right)}^{Int \text{♀}}}C*\left( 1-x \right) \right) \right)*\left( 1-\theta\right)$$

 Equation 14b(i) (Int)

The total number of females laying eggs in the intervention site in each cycle consist of those who hatched and mated in the refugia but dispersed to the intervention site ($N_{Int (G)}^{Ref \text{♀}}$) and those who hatched, mated and stayed in the intervention site ($N_{Int \left( G \right)}^{Int \text{♀}}$):

$$N_{Int (G)}^{Ref \text{♀}}= \sum_{z_{I Int \left( G \right)}^{Ref\text{♀}}= -\infty}^{\infty} F_{z_{I Int \left( G \right)}^{Ref\text{♀}}}$$

 Equation 14c(i) (Int)

$$N_{Int \left( G \right)}^{Int \text{♀}}= \sum_{z_{I Int \left( G \right)}^{Int \text{♀}}= -\infty}^{\infty} F_{z_{I Int \left( G \right)}^{Int \text{♀}}}$$

 Equation 14d(i) (Int)

The updated mean PRS for each cycle which originally hatched in the refugia is:

$$\bar{z}_{I Int \left( G \right)}^{P Ref \text{♀}}=\left( \sum_{z_{I Int \left( G \right)}^{Ref \text{♀}}= -\infty}^{\infty} F_{z_{I Int \left( G \right)}^{Ref\text{♀}}}z_{I \left( G=0 \right)}^{Ref\text{♀}} \right)/N_{Int (G)}^{Ref \text{♀}}$$

 Equation 14e(i) (Int)

And for those originally hatched in the intervention site:

$$\bar{z}_{I Int \left( G \right)}^{P Int \text{♀}}=\left( \sum_{z_{I Int \left( G \right)}^{Int \text{♀}}= -\infty}^{\infty} F_{z_{I Int \left( G \right)}^{Int \text{♀}}}z_{I \left( G=0 \right)}^{Int\text{♀}} \right)/N_{Int (G)}^{Int \text{♀}}$$

 Equation 14f(i) (Int)

Female insecticide selection differentials in the intervention site for any cycle are calculated as:

$$S_{I Int \left( G \right)}^{\text{S}\text{ }Ref\text{ ♀}}=\bar{z}_{I Int \left( G \right)}^{S Ref\text{ ♀}}- \bar{z}_{I \left( G=0 \right)}^{Ref \text{♀}}$$

 Equation 14g(i) (Int)

$$S_{I Int \left( G \right)}^{S Int \text{♀}}=\bar{z}_{I Int \left( G \right)}^{S Int \text{♀}}- \bar{z}_{I \left( G=0 \right)}^{Int\text{♀}}$$

 Equation 14h(i) (Int)

Fitness costs are implemented using Equation 11a(ii) giving the overall selection differential for a cycle. Responses are calculated separately, for the females who hatched and mated in the refugia ($R_{I Int \left( G \right)}^{Ref}$) and females who hatched and mated in the intervention site ($R_{I Int \left( G \right)}^{Int}$):

$$R_{I Int \left( G \right)}^{Ref}= h^{2}\left( \frac{S_{I Int \left( G \right)}^{Ref \Phi\text{♀}}+ S_{I}^{Ref \Phi\text{♂}}}{2} \right)\beta$$

 Equation 14i(i) (Int)

$$R_{I Int \left( G \right)}^{Int}= h^{2}\left( \frac{S_{I Int \left( G \right)}^{Int S\Phi\text{♀}}+ S_{I}^{Int S\Phi\text{ ♂}}}{2} \right)\beta$$

 Equation 14j(i) (Int)

**Gonotrophic Cycle 2 and Beyond: Tracking Females Laying Eggs in the Refugia**

For subsequent cycles, the frequency of females laying eggs in the refugia needs to be calculated. This requires two calculations. The first calculation tracks females who hatched, and mated in the refugia.

$$F_{z_{I Ref \left( G \right)}^{Ref\text{♀}}}=\left( F_{z_{I Ref \left( G-1 \right)}^{Ref\text{♀}}}\left( 1-\theta\right)\rho\right)+\left( \left( \left( F_{z_{I Int \left( G-1 \right)}^{Ref\text{♀}}}\left( 1-x \right) \right)+\left( F_{z_{I Int \left( G-1 \right)}^{Ref\text{♀}}}c_{i}xK_{i}^{F} \right)+\left( F_{z_{I Int \left( G-1 \right)}^{Ref\text{♀}}}c_{j}x\bar{K}_{j}^{F} \right)+ \left( F_{z_{I Int \left( G-1 \right)}^{Ref\text{♀}}}c_{ij}\Lambda_{i|ij}^{\text{♀}}xK_{i}^{F} \right)+ \left( F_{z_{I Int \left( G-1 \right)}^{Ref\text{♀}}}c_{ij}\Lambda_{j|ij}^{\text{♀}}x\bar{K}_{j}^{F} \right)+ \left( F_{z_{I Int \left( G-1 \right)}^{Ref\text{♀}}}c_{ij}\Lambda_{ij|ij}^{\text{♀}}xK_{i}^{F}\bar{K}_{j}^{F} \right) \right)*\theta\rho\right)$$

Equation 14a(ii)(Ref)

The second calculation tracks females who hatched and mated in the intervention site, who previously joined the refugia (and stay again) or those newly joining from the intervention site (having undergone insecticide selection):

$$F_{z_{I Ref \left( G \right)}^{Int \text{♀}}}= \left( F_{z_{I Ref \left( G-1 \right)}^{Int\text{♀}}}\left( 1-x \right)\left( 1-\theta\right)\rho\right)+ \left( \left( F_{z_{I Int \left( G-1 \right)}^{Int \text{♀}}}c_{i}xK_{i}^{F} \right)+\left( F_{z_{I Int \left( G-1 \right)}^{Int \text{♀}}}c_{j}x\bar{K}_{j}^{F} \right)+ \left( F_{z_{I Int \left( G-1 \right)}^{Int \text{♀}}}c_{ij}\Lambda_{i|ij}^{\text{♀}}xK_{i}^{F} \right)+ \left( F_{z_{I Int \left( G-1 \right)}^{Int \text{♀}}}c_{ij}\Lambda_{j|ij}^{\text{♀}}x\bar{K}_{j}^{F} \right)+ \left( F_{z_{I Int \left( G-1 \right)}^{Int \text{♀}}}c_{ij}\Lambda_{ij|ij}^{\text{♀}}xK_{i}^{F}\bar{K}_{j}^{F} \right)+ \left( F_{z_{I Int \left( G-1 \right)}^{Int \text{♀}}}(1-x) \right)*\theta\rho\right)$$

Equation 14b(ii) (Ref)

$F_{z_{I Ref \left( G \right)}^{Ref\text{♀}}}$ is transferred to Equations 14c(i)(Ref) and 14e(i)(Ref) and $F_{z_{I Ref \left( G \right)}^{Int \text{♀}}}$ is transferred to Equations 14d(i)(Ref) and 14f(i)(Ref).

**Gonotrophic Cycle 2 and Beyond: Laying Eggs in the Intervention Site**

A parallel process occurs in the intervention site. The frequency of females laying eggs in the intervention site is. First, laying eggs is those who hatched and mated in the refugia (and dispersed to the intervention site previously), who remain in the intervention site (undergoing insecticide selection) or are newly dispersing from the refugia:

$$F_{z_{I Int \left( G \right)}^{Ref\text{♀}}}=\left( F_{z_{I Ref \left( G-1 \right)}^{Ref\text{♀}}}\theta\rho\right)+\left( \left( \left( F_{z_{I Int\left( G-1 \right)}^{Ref\text{♀}}}xc_{i}K_{i}^{F} \right)+\left( F_{z_{I Int\left( G-1 \right)}^{Ref\text{♀}}}xc_{j}\bar{K}_{j}^{F} \right)+\left( F_{z_{I Int\left( G-1 \right)}^{Ref\text{♀}}}xc_{ij}\Lambda_{i|ij}^{\text{♀}}K_{i}^{F} \right)+\left( F_{z_{I Int\left( G-1 \right)}^{Ref\text{♀}}}xc_{ij}\Lambda_{j|ij}^{\text{♀}}\bar{K}_{j}^{F} \right)+\left( F_{z_{I Int\left( G-1 \right)}^{Ref\text{♀}}}xc_{ij}\Lambda_{ij|ij}^{\text{♀}}{K_{i}^{F}\bar{K}}_{j}^{F} \right)+\left( F_{z_{I Int\left( G-1 \right)}^{Ref\text{♀}}}\left( 1-x \right) \right) \right)*\left( 1-\theta\right)*\rho\right)$$

Equation 14a(ii) (Int)

Second is females who hatched and mated in the intervention site, who previously dispersed to the refugia and now disperse back to the intervention site and those who stayed in the intervention site (and undergo insecticide selection):

$$F_{z_{I Int \left( G \right)}^{Int \text{♀}}}= \left( F_{z_{I Ref \left( G-1 \right)}^{Int \text{♀}}}\theta\rho\right)+\left( \left( \left( F_{z_{I Int\left( G-1 \right)}^{Int\text{♀}}}xc_{i}K_{i}^{F} \right)+ \left( F_{z_{I Int\left( G-1 \right)}^{Int\text{♀}}}xc_{j}\bar{K}_{j}^{F} \right)+ \left( F_{z_{I Int\left( G-1 \right)}^{Int\text{♀}}}xc_{ij}\Lambda_{i|ij}^{\text{♀}}K_{i}^{F} \right)+ \left( F_{z_{I Int\left( G-1 \right)}^{Int\text{♀}}}xc_{ij}\Lambda_{j|ij}^{\text{♀}}\bar{K}_{j}^{F} \right)+ \left( F_{z_{I Int\left( G-1 \right)}^{Int\text{♀}}}xc_{ij}\Lambda_{ij|ij}^{\text{♀}}K_{i}^{F}\bar{K}_{j}^{F} \right)+\left( F_{z_{I Int\left( G-1 \right)}^{Int\text{♀}}}\left( 1-x \right) \right) \right)*\left( 1-\theta\right)*\rho\right)$$

Equation 14b(ii) (Int)

$F_{z_{I Int \left( G \right)}^{Ref\text{♀}}}$ is transferred to Equation 14c(i) (Int) and 14e(i) (Int), and $F_{z_{I Int \left( G \right)}^{Int \text{♀}}}$is transferred to equations 14d(i) (Int) and 14f(i) (Int).

**Calculating the Overall Selection Response for Multiple Gonotrophic Cycles with Dispersal**

Responses (i.e., inherited changes in the PRS) need to be weighted by the number of eggs laid in each cycle; obviously the total number of eggs laid each cycle will decline due to a reduction in female population size due by mortality caused by insecticides and natural mortality. The total number of oviposition events (and therefore proportional to the total number of eggs laid) in the Refugia is therefore:

$$N_{o Ref}^{Total\text{♀}}= N_{o Ref}^{Ref\text{♀}}+N_{o Ref}^{Int\text{♀}}$$

 Equation 14k(Ref)

$N_{o Ref}^{Ref\text{♀}}$ is the total contribution from females born (and mated) in the refugia:

$$N_{o Ref}^{Ref\text{♀}}= \sum_{G=1}^{G_{max}} N_{Ref \left( G \right)}^{Ref\text{♀}}$$

Equation 14l(Ref)

$N_{o Ref}^{Int\text{♀}}$ is the total contribution from females born (and mated) in the intervention site:

$$N_{o Ref}^{Int\text{♀}}= \sum_{G=1}^{G_{max}} N_{Ref \left( G \right)}^{Int \text{♀}}$$

Equation 14m(Ref)

And correspondingly the total number of oviposition events in the intervention site:

$$N_{o Int}^{Total\text{♀}}= N_{o Int}^{Int\text{♀}}+N_{o Int}^{Ref \text{♀}}$$

Equation 14k(Int)

$N_{o Int}^{Int\text{♀}}$ is the total contribution from females born (and mated) in the intervention site:

$$N_{o Int}^{Int\text{♀}}= N_{Int\left( G=1 \right)}^{Int\text{♀}}+ N_{Int\left( G=2 \right)}^{Int\text{♀}}+ N_{Int\left( G=3 \right)}^{Int\text{♀}} \ldots$$

Equation 14(Int)

$N_{o Int}^{Ref \text{♀}}$ is the total contribution from females born (and mated) in the refugia:

$$N_{o Int}^{Ref \text{♀}}= N_{Int\left( G=1 \right)}^{Ref \text{♀}}+ N_{Int\left( G=2 \right)}^{Ref \text{♀}}+ N_{Int\left( G=3 \right)}^{Ref \text{♀}} \ldots$$

Equation 14m(Int)

The responses for each cycle are weighted to calculate the overall responses. In the intervention site this is for eggs laid by females originally emerged (and mated) in the intervention site:

$$R_{I Int}^{T Int}= \sum_{G=1}^{G_{max}} \left( R_{I Int\left( G \right)}^{Int}+{{(\alpha}_{JI}R}_{Int \left( G \right)}^{Int}) \right)\frac{N_{Int\left( G \right)}^{Int\text{♀}}}{N_{o Int}^{Int\text{♀}}}$$

Equation 14n(Int)

And for females originally emerged (and mated) in the refugia:

$$R_{I Int}^{T Ref}= \sum_{G=1}^{G_{max}} \left( R_{I Int\left( G \right)}^{Ref}+{{(\alpha}_{JI}R}_{Int \left( G \right)}^{Ref}) \right)\frac{N_{Int\left( G \right)}^{Ref\text{♀}}}{N_{o Int}^{Ref\text{♀}}}$$

Equation 14o(Int)

The mean PRS of the eggs (in the next generation) in the intervention site is:

$$\bar{z}_{I}^{Int^{''}}=\frac{\left( N_{o Int}^{Int\text{♀}}\left( \bar{z}_{I}^{Int}+ R_{I Int}^{T Int} \right) \right)+\left( N_{o Int}^{Ref\text{♀}}\left( \bar{z}_{I}^{Ref}+ R_{I Int}^{T Ref} \right) \right)}{N_{o Int}^{Total\text{♀}}}$$

Equation 14p(Int)

In the refugia this is as follows: Weighting the responses in the refugia is for eggs laid by females originally emerged (and mated) in the refugia (Equation 15n(Ref))

$$R_{I Ref}^{T Ref}= \sum_{G=1}^{G_{max}} \left( R_{I Ref\left( G \right)}^{Ref}+{{(\alpha}_{JI}R}_{Ref \left( G \right)}^{Ref}) \right)\frac{N_{Ref\left( G \right)}^{Ref\text{♀}}}{N_{o Ref}^{Ref\text{♀}}}$$

Equation 14n(Ref)

And for females originally emerged (and mated) in the intervention site:

$$R_{I Ref}^{T Int}= \sum_{G=1}^{G_{max}} \left( R_{I Ref\left( G \right)}^{Int}+{{(\alpha}_{JI}R}_{Ref \left( G \right)}^{Int}) \right)\frac{N_{Ref\left( G \right)}^{Int\text{♀}}}{N_{o Ref}^{Int\text{♀}}}$$

Equation 14o(Ref)

The mean of the eggs (the next generation) in the refugia is:

$$\bar{z}_{I}^{Ref^{''}}=\frac{\left( N_{o Ref}^{Ref\text{♀}}\left( \bar{z}_{I}^{Ref}+ R_{I Ref}^{T Ref} \right) \right)+\left( N_{o Ref}^{Int\text{♀}}\left( \bar{z}_{I}^{Int}+ R_{I Ref}^{T Int} \right) \right)}{N_{o Ref}^{Total\text{♀}}}$$

Equation 14p(Ref)
