# Supplementary material for: Simulating dynamic insecticide selection pressures for resistance management in mosquitoes assuming polygenic resistance
Source: PLoS Comput Biol. 2025 Apr 28;21(4):e1012944. doi: 10.1371/journal.pcbi.1012944 (PMC12058183; doi:10.1371/journal.pcbi.1012944)
Supplement: S7 File — (DOCX) [file pcbi.1012944.s007.docx]

**S7 File: Examples of Insecticide Resistance Management Strategies**

Here we detail the IRM strategies and decision-making rules used in the models. We make a distinction between how insecticides are deployed (e.g., monotherapies, mixtures, micro-mosaics, and combinations) and how insecticides are switched (e.g., rotations, sequences). With more complex IRM deployments there can be more complex deployment decisions. Much of the language around IRM strategies is based on two-insecticides being available. We generalize this to descriptions of IRM strategies which are extendable to multiple insecticides (Table A in S7 File) and provide visual examples (Fig A to Fig D in S7 File). The example simulations used parameter values which best visually illustrated the strategies and should not be compared against one another. These examples highlight the capability of the “polysmooth” and “polytruncate” models.

| **Table A: Describing Insecticide Resistance Management Strategies: Deployments, Switches and Thresholds.** | |
| --- | --- |
| **Insecticide Deployment Strategy** | **Insecticide Switching Strategies available within each deployment strategy** |
| MONOTHERAPIES: Only one insecticide is deployed at any time. Example: Fig A in S7 File. | SEQUENCES: Insecticide$i$ is deployed continuously until reaching the withdrawal threshold. Insecticide $i$ is then withdrawn and is replaced by the next insecticide ($j$). Insecticide $j$ is deployed continuously, until it too reaches the withdrawal threshold. If fitness costs have brought resistance to insecticide $i$ below the “return” threshold insecticide $i$ is re-available for deployment. |
|  | ROTATIONS: Insecticides are switched at each opportunity (e.g., every year for IRS, every 3 years for ITNs). The rotation strategy fails when either (1) no insecticides are available to deployed (all withdrawn due to resistance), or (2) only a single insecticide is available, in this case a rotation is not possible and the simulation terminates. |
|  | ADAPTIVE ROTATIONS: Same as rotations except, can deploy insecticides in sequence if required. Defaults to rotations when possible. |
| MIXTURES: Two different insecticides are deployed in the same formulation. Consequently any mosquito contacting the mixture formulation encounters both insecticides simultaneously.  Examples: Fig B and Fig C in S7 File. | ROTATE NOVEL PARTNERS: The “common” insecticide partner is used in all mixtures. The “novel” insecticide is changed each deployment interval. Deployment decisions are made only on extent of resistance to the novel insecticide partners. |
|  | SEQUENCE NOVEL PARTERS: As for “rotate novel partners” described above, except the novel insecticide is deployed in sequence. |
|  | ROTATE MIXTURE FORMULATION: The mixture formulation is rotated at each opportunity. If any insecticide in the mixture reaches the withdrawal threshold the insecticide is no longer available for deployment and therefore the mixture is also not available for deployment. |
|  | SEQUENCE MIXTURE FORMULATION: The mixture formulation is deployed in sequence. This differs from the “rotate novel partners” strategy because if any insecticide in the mixture exceeds the withdrawal threshold the insecticide is no longer available for deployment and therefore the mixture is also not available for deployment. |
| MICRO-MOSAICS:  Two insecticides in the same village, but each household receives only one insecticide.  Example: Fig D in S7 File | INDIVIDUAL SEQUENCE: The deployment decisions are made separately on each insecticide separately. If $i$ reaches the withdrawal threshold, it is replaced with $k$; this does not affect insecticide $j$ which continues to be deployed. |
|  | FULL ROTATION: Both insecticides deployed in the micro-mosaic are rotated at each opportunity. |
|  | PARTIAL ROTATION: Only one of the insecticides in the micro-mosaic is rotated. One insecticide is always deployed. Deployment decisions are made on resistance levels to the rotating insecticides. |
| COMBINATIONS:  Insecticides are deployed as ITN and IRS. Households may receive ITN, IRS or both. Example: Fig E in S7 File. | ROTATE IRS: The insecticide on the ITN is fixed. The IRS insecticides are rotated at each opportunity. Deployment decisions are made only on the level of resistance to the insecticides used in the IRS. The insecticide used in the ITN cannot be used as an IRS. |
|  | SEQUENCE IRS: The insecticide on the ITN is fixed. The IRS insecticides are deployed in sequence. Deployment decisions are made only on the level of resistance to the IRS. The insecticide used in the ITN cannot be used as an IRS. |


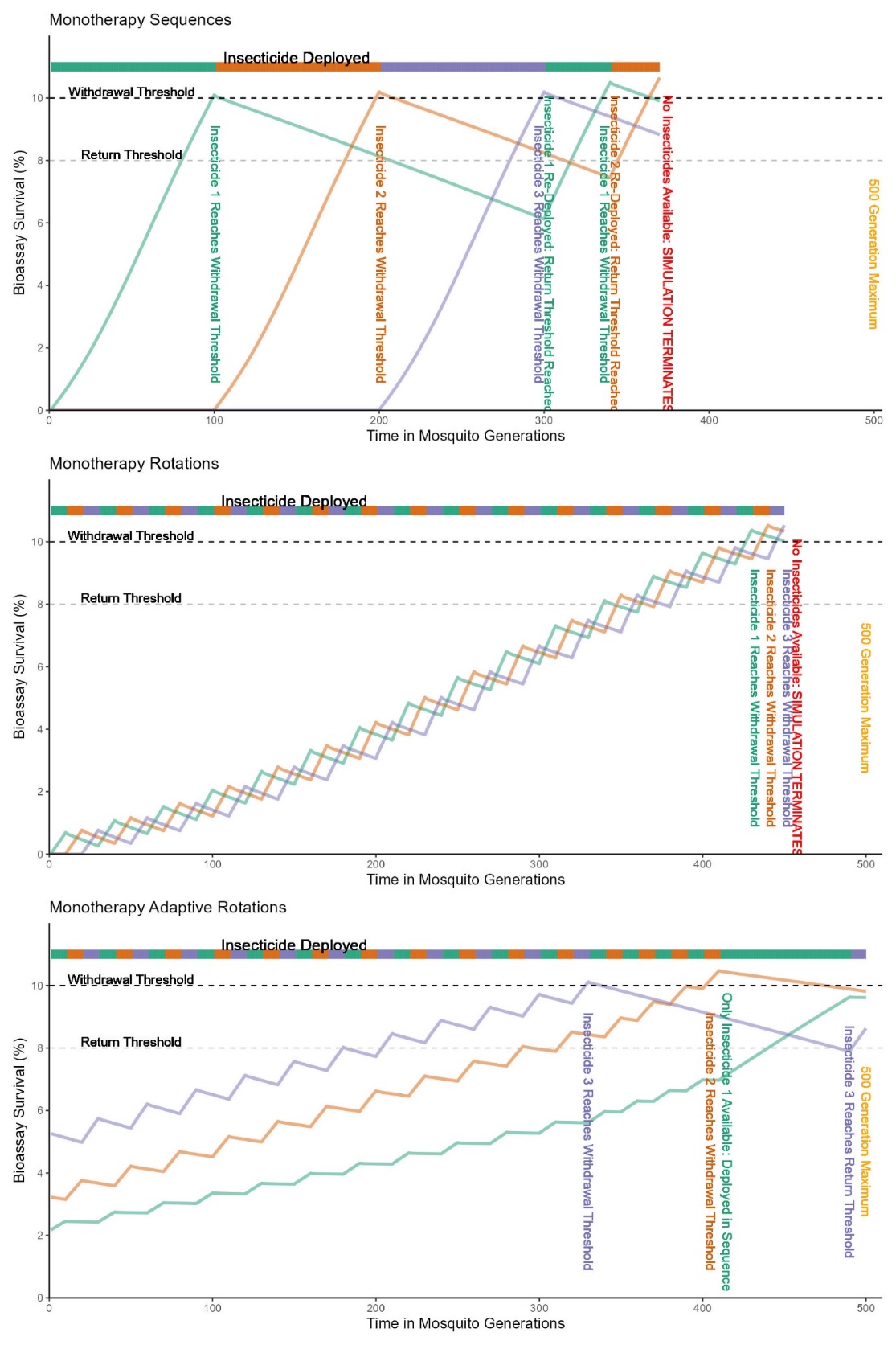


**Fig A: Illustrative examples of the Monotherapies deployment strategy.** Three insecticides are available which are distinguished by different colours. **Top: Sequences:** Insecticides are switched by sequence rules. **Middle: Rotations:** Insecticides are switched by rotation rules. **Bottom: Adaptive Rotations:** Insecticides are switched by adaptive rotation rules. These rules are given in Table A in S7 File.


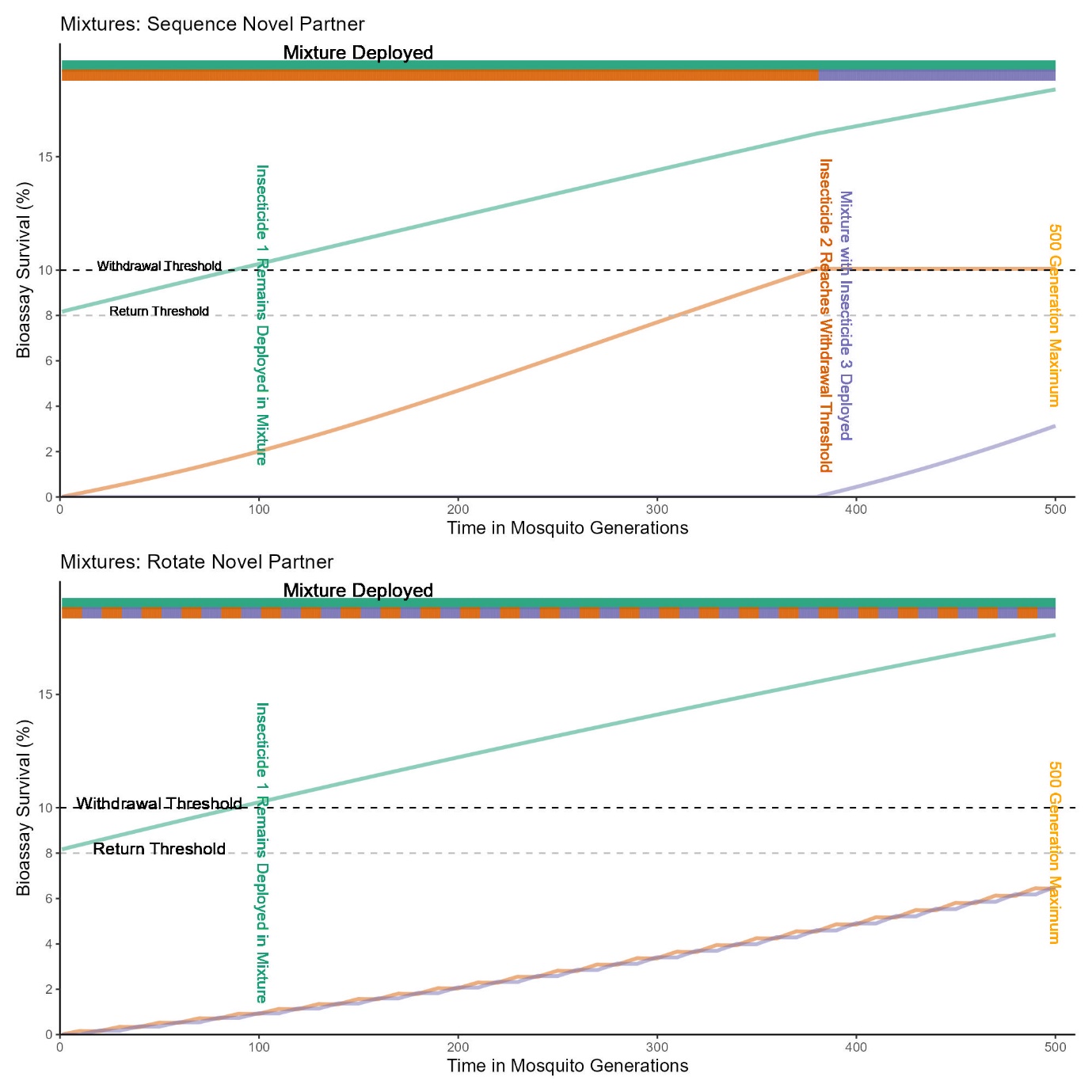


**Fig B: Illustrative example of the Mixtures deployment strategy with switching decisions on the novel partner.** Three insecticides are available which are distinguished by the three colours. **Top: Sequence Novel Partners:** Assuming all mixtures share a common partner (i.e., a pyrethroid, shown in green). The “pyrethroid” insecticide is deployed even after it has exceeded the withdrawal threshold. Deployment decisions are made on the novel partner. **Bottom: Rotations Novel Partners:** As with Monotherapy Rotations, if the mixture of insecticides $i$ &$j$ was the only mixture available and would be immediately redeployed (in sequence) the Rotation Novel Partners strategy is regarded as “failed” and the simulation terminates. These rules are given in Table A in S7 File.


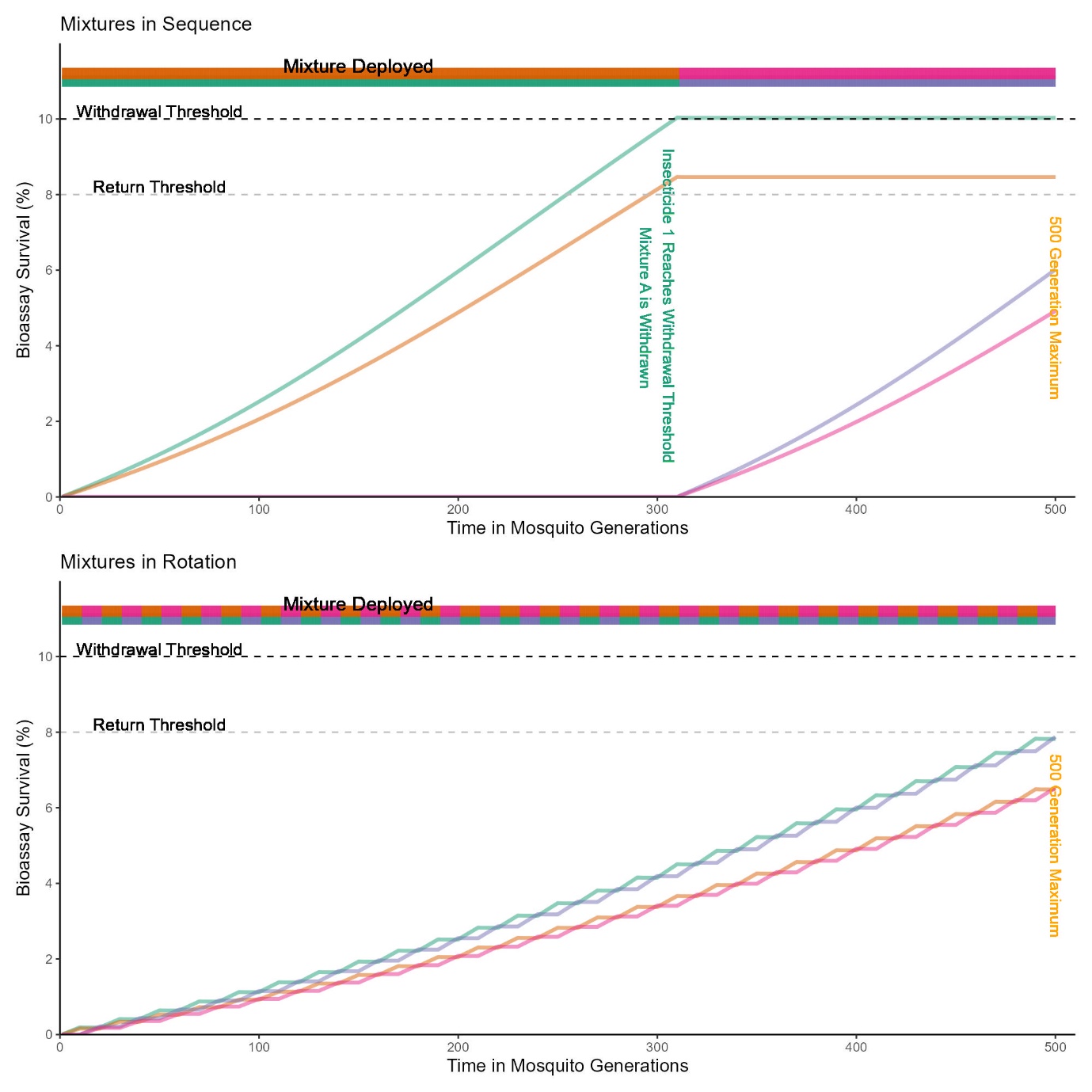


**Fig C: Illustrative example of the mixture deployment strategy with switching decisions on the mixture.** Four insecticides are available which results in two different mixture formulations. The four constituent insecticides are distinguished by different colours. **Top: Sequence Mixture Formulation.**  Each mixture formulation is deployed in sequence. Therefore, if either of the insecticides in the mixture reaches the pre-defined withdrawal threshold, then this means the mixture itself is withdrawn. The next mixture formulation in the sequence is then deployed. If a failed insecticide is in multiple mixtures, all those mixture formulations are withdrawn. **Bottom: Rotate Mixture Formulation.** The mixture formulation is rotated at each opportunity. For example, mixture “A” of insecticides $i$ and $j$ is rotated with mixture “B” of insecticides $k$ and $m$. Here, the mixture formulation is rotated at each available opportunity. Note, if one of the insecticides in the mixture reaches the withdrawal threshold the mixture is withdrawn from being available for deployment. These rules are given in Table A in S7 File.


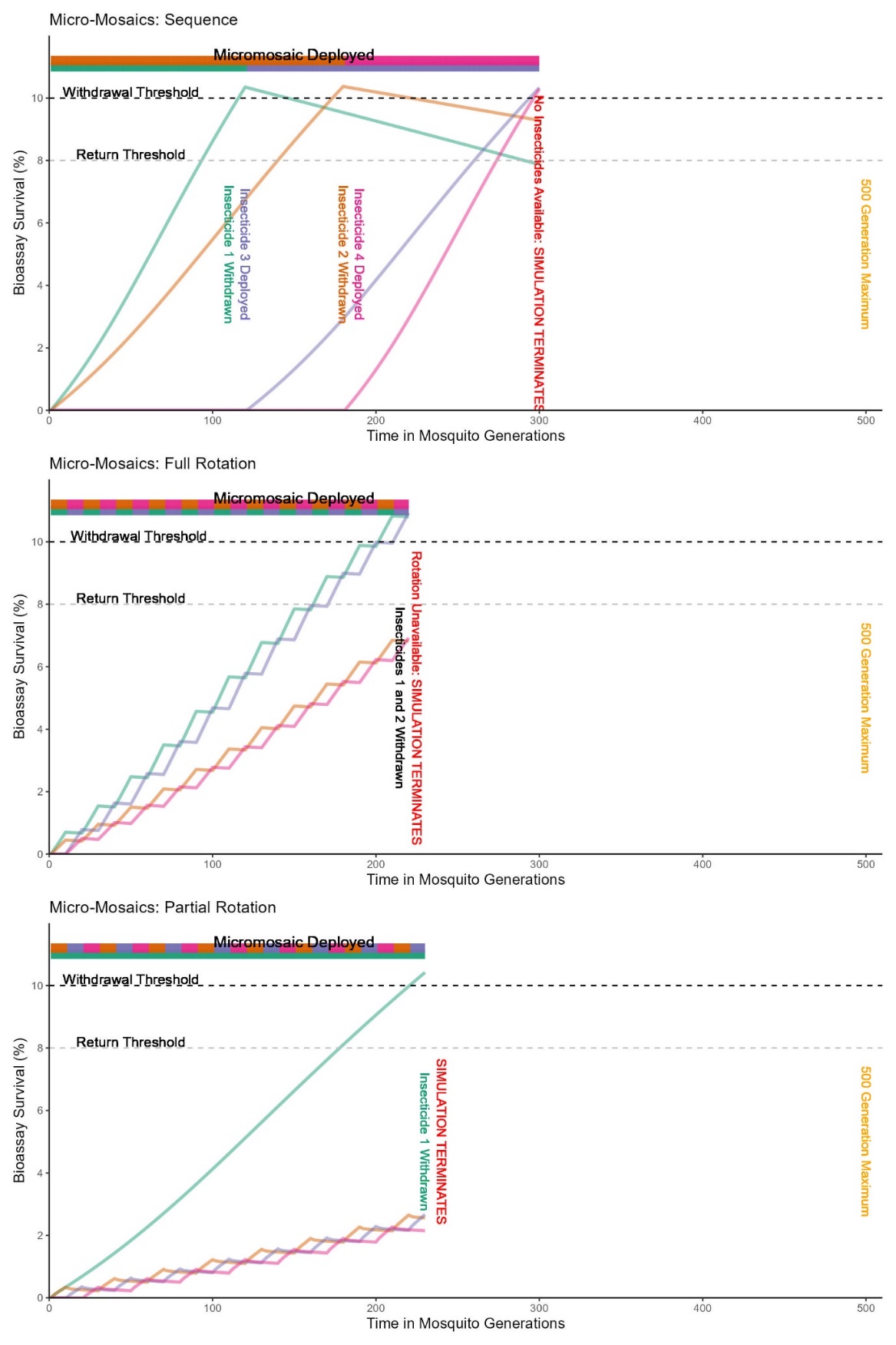


**Fig D: Illustrative example of the Micro-Mosaic deployment strategy.** Four insecticides are available which are distinguished by different colours. **Top: Individual Sequences:** The Micro-Mosaic consists of individual insecticides, each of which can be independently assessed for resistance and can therefore be withdrawn independently of each other. **Middle: Full Rotation.** Here, both insecticides are rotated out at each deployment opportunity. **Bottom: Partial Rotation.** Here, one of the insecticides remained deployed throughout (e.g., due to being the cheaper insecticide) with the other (potentially more expensive) insecticides are rotated.


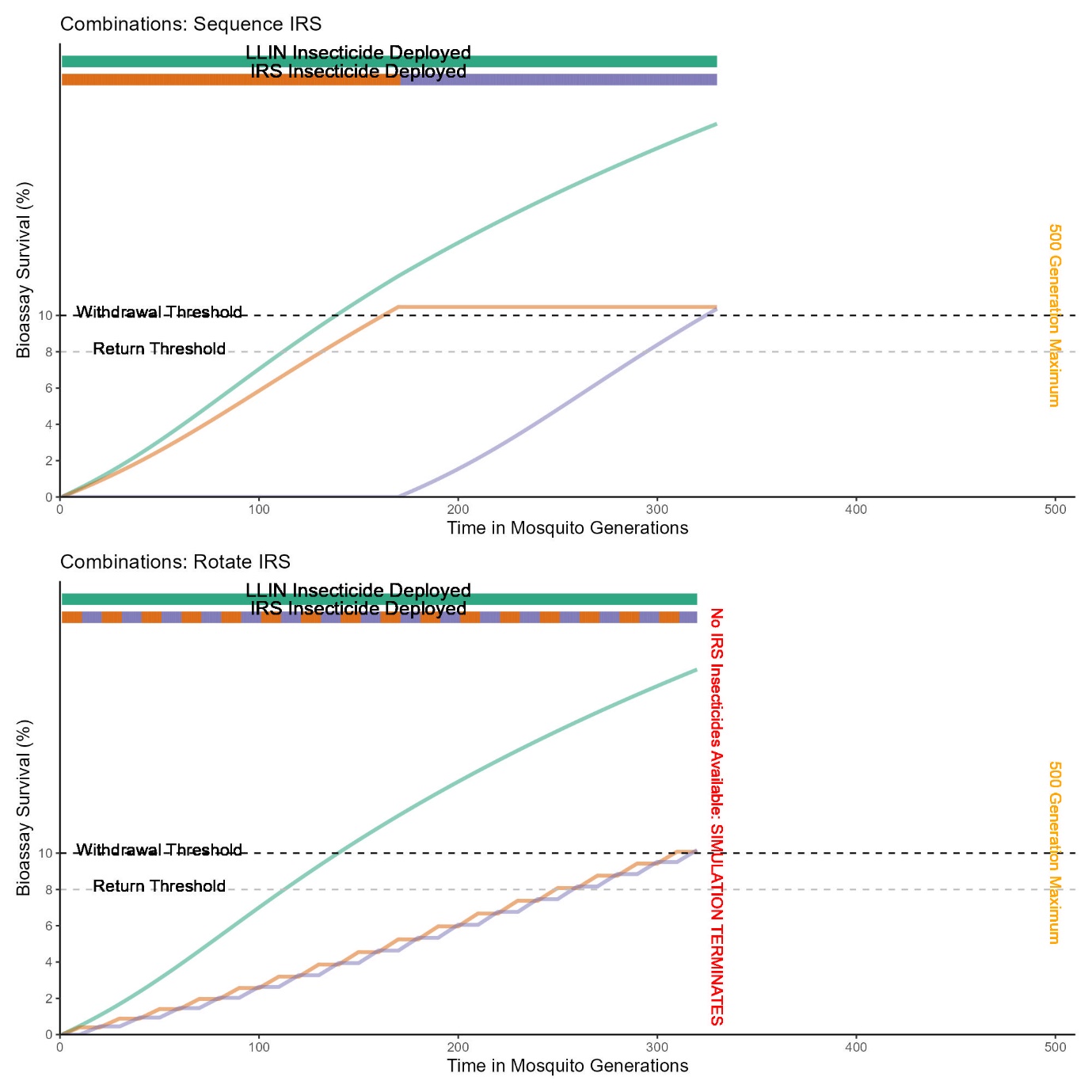


**Fig E: Illustrative examples of the Combinations deployment strategy with switching decision based on the IRS insecticides.** Three insecticides are available which are distinguished by different colours. **Top: Sequence IRS:** Each IRS insecticide is deployed in sequence until it reaches the withdrawal threshold, at which point it is withdrawn and replaced with the next available IRS insecticide in the sequence. The ITN and IRS can have different deployment intervals (ITNs 3 years, ~30 generations and IRS yearly, ~10 generations). Deployment decisions are only made on the levels of resistance to the IRS only. **Bottom: Rotate IRS:** The IRS insecticides are rotated at each opportunity, for example yearly (as in this example).
